# *β*-sheet Topology Prediction with High Precision and Recall for *β* and Mixed *α/β* Proteins

**DOI:** 10.1371/journal.pone.0032461

**Published:** 2012-03-09

**Authors:** Ashwin Subramani, Christodoulos A. Floudas

**Affiliations:** Department of Chemical and Biological Engineering, Princeton University, Princeton, New Jersey, United States of America; University of South Florida College of Medicine, United States of America

## Abstract

The prediction of the correct 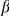-sheet topology for pure 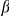 and mixed 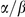 proteins is a critical intermediate step toward the three dimensional protein structure prediction. The predicted beta sheet topology provides distance constraints between sequentially separated residues, which reduces the three dimensional search space for a protein structure prediction algorithm. Here, we present a novel mixed integer linear optimization based framework for the prediction of 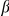-sheet topology in 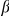 and mixed 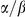 proteins. The objective is to maximize the total strand-to-strand contact potential of the protein. A large number of physical constraints are applied to provide biologically meaningful topology results. The formulation permits the creation of a rank-ordered list of preferred 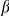-sheet arrangements. Finally, the generated topologies are re-ranked using a fully atomistic approach involving torsion angle dynamics and clustering. For a large, non-redundant data set of 2102 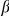 and mixed 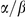 proteins with at least 3 strands taken from the PDB, the proposed approach provides the top 5 solutions with average precision and recall greater than 78%. Consistent results are obtained in the 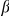-sheet topology prediction for blind targets provided during the CASP8 and CASP9 experiments, as well as for actual and predicted secondary structures. The 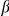-sheet topology prediction algorithm, BeST, is available to the scientific community at http://selene.princeton.edu/BeST/.

## Introduction

Many approaches have been introduced to address the three dimensional protein structure prediction problem, and can be divided broadly into homology modeling, fold recognition and first principles based methods. Recent reviews provide detailed accounts of each of these classes of protein structure prediction techniques [1]–[4].

The hierarchical theory of protein folding has gained a lot of support over the last few decades [5]–[9]. A number of first principles based structure prediction algorithms use the hierarchical theory of protein folding to divide the extremely complex protein structure prediction problem into a number of subproblems tackling local and tertiary structural features of the protein [10]–[15]. An important intermediate step is the prediction of the arrangement of 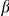-strands in a protein, that is the 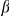-sheet topology prediction problem. Given that the knowledge gained at each intermediate step of a hierarchical algorithm is used to narrow the three dimensional search space of the protein, the 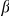-sheet prediction stage provides invaluable information with respect to spatial proximity of non-consecutive amino acids along the sequence of the protein chain. Further, the importance of the 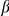-sheet topology is reflected in the fact that an isolated 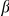-strand can be stabilized only in the presence of a hydrogen bonding ladder with another 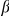-strand in the protein. The main challenge with the prediction of 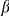-sheets is the presence of non-local hydrogen bonds. It is noteworthy that the 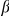-sheet topology prediction is regarded as the primary bottleneck towards the three dimensional structure prediction, as evidenced through all CASP blind predictions. This is also evidenced from Table 1 and Table S1 which show the number of possible 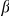-sheets for a given number of 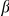-strands.

**Table 1:** The number of motifs possible for a protein with 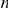 strands (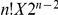).

Strands	Number of Motifs	Strands	Number of Motifs
2	2	3	12
4	96	5	960
6	11520	7	161280
8	2580480	9	46448640
10	928972800	11	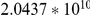

In order to determine rules based on conformational and biological observations of proteins, 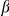-sheet topologies observed in nature have been categorized into a broad set of categories. Some of the earliest work in this direction classified proteins based on tertiary structure patterns [16], [17]. Subsequently, protein structures have been classified in large databases like SCOP and CATH, based on the structural family that they belong to [18]–[21].

Considerable work has been carried out over the years, aiming to determine conformational and structural restrictions in 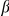 and mixed 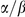 proteins. Orengo and Thornton [22] classified mixed 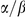 proteins into broad categories: the 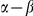 sandwich where 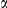 helices and 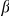-strands form unique layers like a sandwich, and the 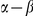 rolls where the 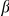-sheet forms folds or rolls, thus creating a cradle for the 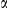-helices. Similarly, extensive analysis on the extraction and classification of the greek key motif in 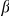-sheets has been presented by Hutchinson and Thornton [23]. Research has also aimed to eliminate certain 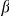-sheet arrangements based on topological arguments. It has been seen that crossover arrangements (i.e. connections between consecutive parallel strands in a given sheet, irrespective of whether they are actually contacting each other) are right handed in nature [24], [25]. Aside from elaborate topological studies which present generic rules for the elimination of strand arrangements, pointers were provided towards elimination of topologies under specific conditions or preferences towards specific arrangements of 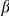-strands. One of the most significant reductions in the allowed topologies comes from the contribution by Richardson [26], who presented a series of simple rules which eliminate a large number of topologies of proteins depending on handedness of connections and the elimination of “knots”, or crossing loops, in the structure. An exhaustive analysis of 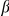-sheets with upto 6 strands was presented [27]. A detailed analysis of the small 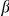-sheets displayed preference of 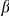-sheets with the same type of contact between pairs of 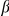-strands, along with a strong rejection of 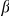-strand arrangements which caused the formation of knots or pretzel-like structures.

A number of approaches have been used to combine the secondary structure prediction, and the 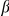-sheet topology prediction problems. These algorithms take as input the primary sequence of the protein, and provide the locations of the 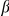-strands in addition to the arrangement of these strands in the three dimensional space. Klepeis and Floudas [28] presented an integer linear optimization based framework, which produces a rank-ordered list of 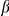-strand arrangements, along with the locations of cysteine-cysteine disulphide bridges. Starting from an amino acid sequence, and following the separation of all 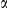-helical residues, their approach creates a superset of possible 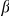-strand regions. Using binary variables to represent residue-to-residue and strand-to-strand contacts, the algorithm predicts the locations and arrangements of the 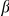-strands by maximize the hydrophobic contact potential of contacting amino acids. Other methods have used database driven algorithms like conditional random fields [29] for the simultaneous prediction of 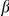-strands and 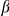-sheets.

A number of methods have employed data mining based methods to derive contact potentials for pairs of residues which are present in 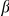 strands [30]–[32]. Initial work in this direction aimed to use residue pair potentials to determine the alignment of strands [33]. The authors used a combination of neural network based secondary structure prediction, a pair potential, and hidden markov models for fold recognition. Other researchers presented work where tripeptides were used to derive potentials for the prediction of 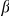-sheets [34]. Similarly, stochastic tree grammar was used for the identification of 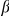-sheets [35], although the test set for this algorithm was very limited. Steward and Thornton [31] used an information theoretic approach to develop sets of tables with pair information values. Similarly, residue pairwise potentials have been derived for residue pairs in contact, as well as offset by up to two amino acids [30]. These pairwise potentials were used to derive a weighted contact potential between 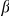-strands, and to derive a rank-ordered list of predicted 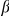-sheet topologies. Cheng and Baldi [32] presented an algorithm BetaPro, which predicts the arrangement of 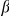-strands in a three stage approach. 2D recursive neural networks were trained to predict the contact potential between amino acid pairs. These pseudo contact potentials are used in a dynamic programming framework to determine the best alignment between pairs of strands. Finally, a greedy algorithm is used to predict the arrangement of 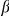-strands, while keeping basic biological constraints. Two approaches were further presented which combined the BetaPro approach with integer optimization and an enhanced greedy approach to accommodate folding cooperativity [36]. Any contact formed between pairs of 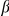-strands resulted in an increase in the strand-to-strand contact potentials of neighboring strands, thus mirroring a zipper-like cooperativity in the formation of contacts between strands that are not sequentially continuous.

Bayesian approaches were introduced for the prediction of 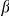-sheet topologies [37]. Separate algorithms were presented for proteins upto six strands, and for proteins with more than six strands. Given the larger amount of available training data, proteins with up to six strands have been modeled using a probabilistic framework by combining residue pairing potentials derived out of apriori knowledge of known 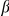-sheet architectures. For proteins with more than six strands, a modified approach to that of Cheng and Baldi [32] was proposed, by introducing penalties for gaps in strand alignments, and by accounting for the formation of 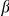-bulges.

In this paper, we propose a framework, BeST, based on mixed-integer linear optimization for the prediction of 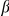-sheet tooplogies in 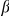 and mixed 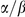 proteins. The algorithm addresses the problem of 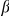-sheet topology prdiction in all non-barrel 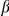 and mixed 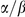 proteins. While a number of theoretical studies have presented the general principles and driving forces in the formation of 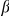-barrels [38], [39], the algorithm presented in this article targets the wide variety of non 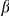-barrel proteins, as it was estimated that almost 95.3% of proteins with extended conformations in the database of sequentially dissimilar proteins used in this study do not have a barrel like formation. The proposed approach is shown in Figure S1 and the algorithm BeST is available at http://selene.princeton.edu/BeST. The only inputs required are the protein sequence and its secondary structure elements. The output is a rank-ordered list of the best predicted 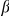-sheet topologies. Large-scale testing on 2102 proteins reveals greater than 78% average precision and recall within the top five predictions.

## Results

The 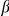-sheet topology prediction approach requires as input only the sequence and secondary structure of a target 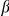 or mixed 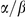 protein. For the assignment of secondary structure for this work, we use the dictionary of secondary structure of proteins, DSSP [40]. Based on the DSSP algorithm, PROMOTIF [41] was used to determine the native arrangement of the 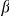-strands of the protein. A number of metrics have been used for the evaluation of the accuracy in prediction of the 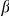-sheet topology. These include Precision, Recall and Matthews Correlation Coefficient, which are described by the following equations, respectively.
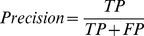
(1)

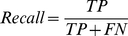
(2)

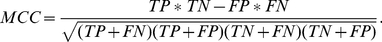
(3)


In the expressions shown, the terms 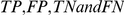 refer to true positive, false positive, true negative and false negative contacts, respectively.

### PDBSelect25 Data Set

In order to extensively test the accuracy of the proposed algorithm, we have used the current PDBSelect25 dataset, where the pairwise sequence similarity between any pair of proteins is less than 25%. The dataset consists of 2102 proteins with at least three strands, with 595 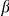 and 1417 mixed 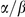 proteins. A graph showing the distribution of the number of proteins with number of strands is provided in Figure S2.

**Figure 1:**
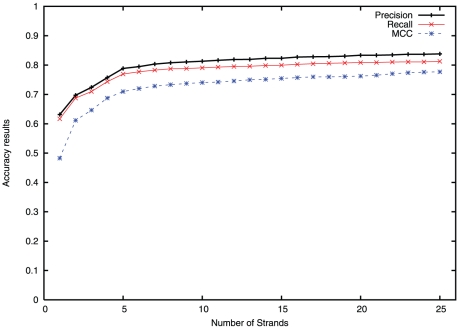
Changes in the average precision, recall and mcc values over the number of solutions.

The weighted average precision, recall, and MCC results for the entire data set, for the top 25 generated solutions are presented in Figure 1. The weighted average precision for any given number of solutions is given by:
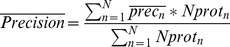
(4)Here, 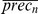 is the average precision observed among all proteins with 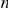 strands, while 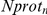 is the number of proteins with 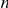 strands. Similar expressions were used for the evaluation of the weighted average recall and correlation coefficient.

As can be seen from Figure 1, we achieve for the top solution precision, recall and MCC of about 63%, 62% and 0.48 respectively. When the top five solutions from the model are considered, the average precision, recall and MCC increase to about 79%, 78% and 0.71, respectively. As the number of solutions considered is increased to 25, the average precision and recall values increase gradually, and take up a value close to 84% and 81%, respectively. Table 1 shows that the number of arrangements of 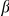-strands increase significantly with the number of strands in the protein. Even with the large number of arrangements of strands possible in proteins, we observe a very large degree of accuracy in average precision and recall values in the top 25 generated solutions over the entire data set.

Figure S3 shows the distribution of the average precision and recall results for varying number of strands, when the number of solutions considered are the top 1, 5, 10, 15, 20 and 25, respectively. It is observed that proteins with smaller number of strands (i.e., less than or equal to 7) reach high values of precision and recall within the top five solutions. As expected, proteins with three strands reach almost 100% precision and recall within the top five solutions. While a small degree of fluctuation is seen with respect to the precision and recall values for proteins with large number of strands, these could be classified as outlier points, given that the number of proteins that these bars represent are very few. Further, as would be expected, we see an almost monotonic change in average precision and recall percentage values as the number of 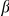-strands (upto 20 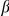-strands) in the proteins increase.

In order to analyze the effectiveness of the dynamic programming algorithm in assigning the right amino acid pairs for any correct strand alignment, the fraction of correctly assigned amino acid pairs for each pair of strands was evaluated for the PDBSelect25 data set. Backbone hydrogen bonds between pairs of amino acids in 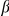-strands are identified. If an amino acid is observed to form backbone hydrogen bonds with more than one partner, the nearest partner is identified as the correct contact. Among the correctly predicted pairs of 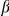-strands in any topology prediction [41], 67.3% of amino acid pairs were correctly aligned to each other.

In a different classification of the results, Figure S4 shows the accuracy of results in 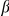 and mixed 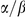 proteins. It is observed that the performance of precision and recall is superior in the case of the mixed 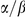 proteins. The explanation is that more local contacts were observed in the case of the mixed 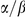 proteins in the PDBSelect25 data set, when compared to the pure 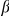 proteins. This could be due to the presence of 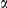-helices in these proteins, which would cause a certain degree of compartmentalization in the 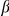-strand register, thus encouraging the formation of local contacts. A second explanation can be postulated based on the derivation of the pseudo-contact potential. The number of mixed 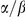 proteins exceed the number of pure 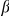 proteins significantly, and the pseudo-contact model may be biased towards the mixed 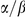 set. Out of the 916 proteins used in the model by Cheng and Baldi [32], only 187 could be considered pure 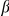 proteins. Finally, it is seen that the mixed 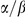 proteins formed a smaller number of sheets than the pure 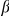 proteins, when the same number of strands were considered. Given that our model aims to maximize contacts between strands, it is expected that indirectly, the model would aim at minimizing the number of 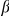-sheets formed. This could potentially be a contributing factor to the improved performance in the mixed 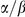 proteins.

### CASP8 and CASP9 Targets

**Figure 2:**
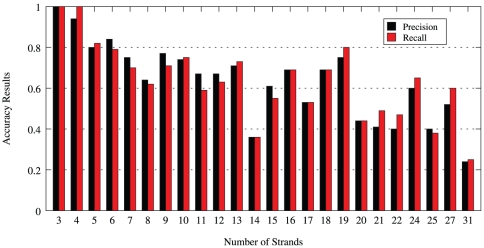
Top five results for proteins in the blind target test set from CASP8 and CASP9.

The model has also been tested on a set of blind targets, provided during recently concluded critical assessment of structure prediction techniques (CASP8 and CASP9) experiments. Table S2 provides a distribution of the number of proteins over the number of strands observed in CASP8 and CASP9 proteins. The precision and recall observed in the top five solutions are presented in Figure 2. As can be seen from the results, the top solution is seen to have an average precision and recall of 66.1% and 65.8%, while the top five solutions have the corresponding values of 75.1% and 74.4%. This shows that the approach produces similar results when tested on a set of blind targets.

The aforementioned results were based on the actual secondary structure assignments, generated out of DSSP [40]. However, in a blind target structure prediction experiment, the true secondary structure assignments are unavailable. To address this problem, we carried out secondary structure prediction using CONCORD [42] (http://helios.princeton.edu/CONCORD), an integer linear optimization based consensus secondary structure prediction approach. The predicted secondary structure for any target protein can contain more, less or the same number of 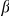-strands as the native secondary structure assignment. In order to evaluate the accuracy of the 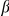-sheet topology prediction algorithm, a map between predicted and actual 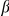-strands is established. All strands which were seen to have a mapped partner are included in the evaluation of results. Based on the predicted secondary structure, the top solution is seen to have an average precision and recall of 62.4% and 61.7%, respectively. The best solution among the top five solutions predicted have precision and recall values of 72.8% and 71.3%, respectively.

## Discussion

We have presented a novel integer linear optimization based algorithm for the prediction of 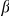-sheet topologies in globular 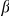 and mixed 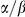 proteins. The algorithm uses strand pairing potentials derived previously [32], and modifies these values to account for any bias to local contacts. The model consists of constraints to enforce structural, physical and biological plausibility on all the topologies that are predicted. Further, a number of constraints have been introduced to restrict the number and types of non-local contacts, thus ensuring a hierarchical nature to the sheet formation process.

The set of constraints that have been introduced are vital to eludicating biologically and structurally meaningful topologies for any given protein. A number of these constraints are based on literature study of existing 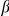 and mixed 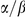 proteins, and can be explained on the basis of steric, entropic or energetic considerations. A significant improvement was seen in the prediction of non-local contacts. This was brought about in part by restricting the total number of local contacts, as well as the introduction of hierarchical constraints defining the possible superset of non-local contacts. This idea of co-operation between the set of strand contacts is consistent with the idea of the zipping and assembly model of protein folding [43]. Dill and co-workers presented this approach to protein folding, wherein the presence of a given set of non-local contacts restricts the movement of the remainder of the chain, thus bringing other non-sequential parts of the primary sequence into spatial proximity [44].

One of the key advantages of the proposed approach is its ability to produce a rank-ordered list of 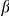-sheet topologies for any target protein. Hence, one would be able to analyze a small set of potential topological solutions. For blind target proteins where the 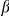-sheet topology is unknown, the knowledge of the top set of solutions, would be helpful in narrowing down the possible set of topology solutions drastically.

The 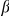-sheet topology prediction algorithm, BeST, is available to the scientific community at http://selene.princeton.edu/BeST.

## Methods

This section presents the 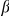-sheet topology prediction model in detail.

The first step for the prediction of the 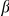-sheet topology of the protein is the identification of the 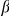-strand regions in the protein. We used the Dictionary of secondary structure of proteins (DSSP) for the identification of 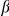 strands [40]. This secondary structure information (including positions of helices in the protein) was used for the generation of residue-residue contact potential generation from the method of Cheng and Baldi [32]. For any pair of strands the best alignment score was determined by sliding one strand across the second in parallel and antiparallel fashion. Let us denote the strand-strand contact potentials as 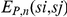 and 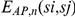, where 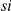 and 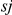 are indices representing strands. Given that the pseudo contact potential is derived from database driven methods, it is expected to have a bias towards local contacts. This can be attributed to the asymmetric distribution of training data available for local and non-local contacts. To correct for the bias towards local contacts, all strand-to-strand contacts were corrected using the following weighting scheme:
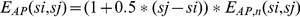
(5)


Similar corrections were carried out for parallel contacts between pairs of strands. We define three sets of binary variables: 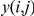 for any residue pair 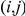 denoting a contact between them; and 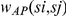 and 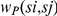 denoting antiparallel and parallel contacts between strands 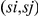, respectively.

Since all contacts are commutative, all binary variables are set up such that the second index is greater than the first. The objective of the model is to maximize the contact potential of the predicted 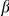-sheet topology, and takes the form:
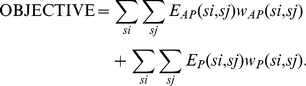
(6)


Several constraints are included to ensure that we obtain physically realistic 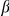-sheet topologies. The first set of constraints link the binary variables for residue-residue contacts (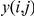) to the binary variables for strand-strand contacts(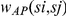 and 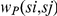). By evaluating the strand-strand contact potentials 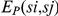 and 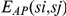, we know the best alignment of any strand pair. We hence define two binary matrices 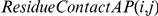 and 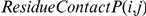, wherein entries are 1 if 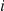 and 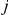 can form a contact at all. In addition, we define parameters 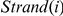 which represent the strand to which residue 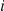 belongs. Of course, this contact would depend on whether the strands they belong to are in contact. This condition can be expressed as:
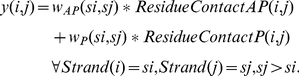
(7)


The constraint expresses the relation between the sets of binary variables by enforcing that the binary variable 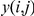 is active if the amino acids can form a contact (represented by 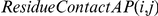 and 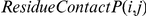) and the corresponding strands are in contact (represented by 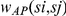 and 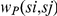). Any two strands 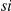 and 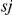 can at most form one type of contact with each other. which becomes:
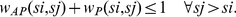
(8)


A strand residue can have a maximum of two contacts. However, this does not mean that the strand itself can only have two contacts. It is possible for a long strand to pair up with more than one strand on one side. Hence, the maximum number of contacts a strand can make is taken as 3. In the entire set of proteins, only four proteins had one strand with four contacts and none had more than four contacts. At the same time, it is required that each strand have atleast one contact. These constraints can be represented as:
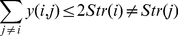
(9)

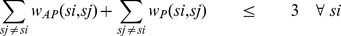
(10)

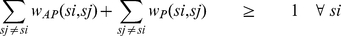
(11)


For a non barrel protein structure, the total number of contacts does not exceed 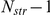, where 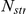 is the total number of strands in the protein. This is expressed as:
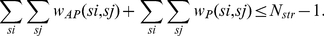
(12)


Since hydrogen bonding and hydrophobic collapse are believed to be the driving force for 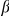-strands to form sheets, the strands aim to minimize exposed area [25], [45]. Moreover, since 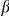 sheets typically form the core of the protein, the possibility of unsatisfied side chains forming hydrogen bonds with the solvent reduces. This exposed area comes about when two unequal strands form a contact, or when a contact is off-centre. In order to ensure that strands with similar lengths form contacts, and that the hydrogen bonding requirements of the strand are satisfied, we enforce that the total residues contacting a given strand should lie between 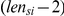 and 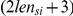, where 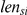 is the length of the strand 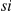. We introduce parameters 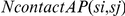 and 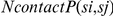, defined as:
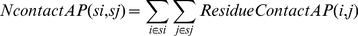
(13)

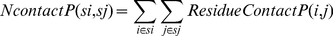
(14)


The constraint can hence be written out such that the total contacts made by any strand 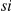, which would be a product of the above mentioned parameter with their respective binary variable, should lie between 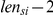 and 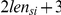. In a number of instances, it is seen that a longer strand pairs with more than one smaller strand on one side. While Equation 9 ensures that any strand residue does not have more than 2 contacts, there could still be a possibility wherein the third contacting strand is predicted to wrap around the first strand, thus satisfying criteria for maximum number of strand and residue contacts. In order to avoid this, we introduce parameters 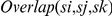, which measure the overlap in contacting residues of strands 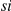 and 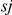, when both contact strand 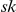. Thus, for any triplet of strands 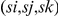 contacting a fourth strand 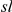, we impose that the overlap of atleast one pair be zero. This is written as:
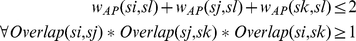
(15)


Similar constraints can be written involving parallel contacts. Further, it was observed that for strands making three antiparallel contacts, at least one contact was made with its neighbors, or one of the edge strands. A number of strands forming 3 contacts made their third contact with a very small strand, which was typically either its own neighbor (by merely proving to be a small extended region following a 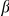-turn) or at either end of the protein sequence, thus resulting in a much smaller impact on entropy loss. This constraint can be written as
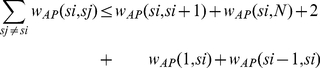
(16)


Based on the idea presented by Przytycka *et al.* recently [46], non-local contacts can be classified into specific classes. In this article, the authors are able to re-create 80% of existing topologies using a small set of rules for bringing sequentially distant strands together. At each implementation of a rule, strands ended up forming new neighbors (i.e. a new set of strands could potentially come together to form a contact). Hence, for any non local contact to form (here, we define a non-local contact to be a contact between strands 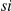 and 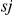 such that 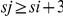), the constraint is expressed as:
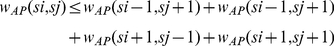
(17)


A few qualifiers for the validity of Equation 17 have been put in place. A circular definition of neighbors has been employed, (i.e. the strand preceding the first strand is taken as the last strand). Similarly, the strand following the last strand is the first one in the sequence. A similar approach was used previously while determining the rules of formation of 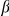-sandwich topologies in pure 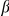 proteins [47], [48]. Further, if a neighbor of a given strand is of length two or three, we move further along in the sequence in the same direction till we identify a valid neighbor to the current strand. The rationale behind this idea is that a very small strand is not influential enough to actually bring sequentially separated parts of the protein together in space. For strands 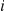 and 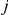 such that 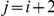, we add two additional terms to the equation, representing the contact of strand 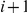 with strands 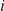 and 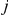. A similar set of equations is written out for parallel contacts.

Driven by hydrophobic collapse, it is expected that the most hydrophobic strands would form the core of the 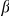 sheet, while the less hydrophobic and shorter strands would form the terminals on both sides [49]. This would mean that the less hydrophobic and shorter strands are likely to have one contact, while the more hydrophobic or longer strands are likely to have more than one contact. The strands are first sorted by length. Within a given length, the strands are sorted by the number of hydrophobic residues. Starting from the smallest strand, we postulate claim that atleast one of the first two would have just one contact. We continue to grow this set in a similar manner, (i.e. atleast 2 of the first four would have one contact each, and so on). The number of such sets created depends on the total number of strands, and one such set is added for every five strands in the entire protein.

Past and recent work in literature have aimed to predict the total number of hydrogen bonds in a protein, given the number of amino acids of the protein. Stickle *et al.* [50] used a small set of 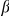 proteins to derive a linear expression for the total number of hydrogen bonds, 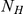, given as:
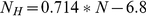
(18)where 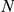 is the number of amino acids of the protein. More recently [51], a much larger data set of proteins was used to derive a modified linear expression of the form:
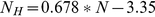
(19)


Both of these equations predict the total number of hydrogen bonds in a globular protein. For the 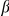-sheet prediction algorithm presented in this article, primary interest lies among the backbone hydrogen bonds formed between amino acids in the 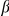-strands of the protein. Past studies presented the total number of hydrogen bonds (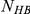) as a function of the fraction of secondary structure elements in the protein [50]:
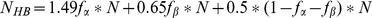
(20)where 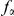 and 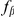 are the fractions of 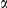-helical and 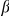-strand residues in the protein, respectively. From Equation 20, we can see that the three terms on the right hand side represent the expected contributions of the helical, extended and coil regions, respectively, to the total number of hydrogen bonds in the protein. In a manner similar to the derivation of linear equations relating the number of hydrogen bonds to the protein length and the fractions of secondary structure elements presented in literature [50], [51], the number of hydrogen bonds associated with the 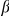 regions of a protein was evaluated. By solving a least squares fit for the total number of hydrogen bonds as a function of the fraction of each secondary structure type, the corrected value of the coefficient for the second term (i.e. the term associated with the 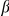 strand regions of the protein) on the right hand side of Equation 20 is 0.638. Since we aim to identify the arrangement of the 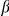-strands of a target protein, the only expression used for the prediction of total number of residue-residue contacts in a protein is the second term of the right hand side of Equation 20 (i.e. 0.638*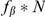). Using this expression, restrictions are introduced on the total number of hydrogen bonds (or “contacts”) between amino acids in 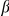-strands, by allowing a 15% error range around the predicted value. Mathematically, this is written as:
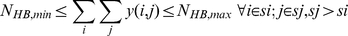
(21)


One of the arrangements of 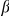-strands conspicuous by its absence is commonly referred to as the “pretzel” [52], and were used recently [53]. For any quartet of 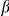-strands (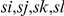) which lie in the same 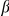-sheet, this constraint prevents the possibility of arrangements which result in the four strands lining up as 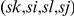 or 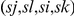. This restriction is written as:
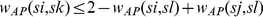
(22)


Recent work has shown specific patterns that have emerged out of the analysis of 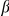-sandwich proteins. These proteins are characterized by a pair of 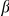-sheets packed against each other like a sandwich [54], [55]. The first observation was the absence of parallel contacts between strands. Further, it was observed that for any non-local strand pairing (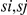) in one sheet, a counter-balancing non-local contact between 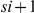 and 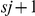 is observed in the opposite sheet, thus forming an “interlock”. These constraints cannot be directly applied to our model, since the aim is to able to develop a prediction algorithm for any kind of 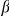 or mixed 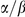 protein. Hence, we generalize this condition to include any quartet of strands (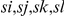) such that 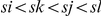 and postulate that an interlock is formed between strand pairs (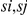) and (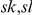), given by the following constraint:
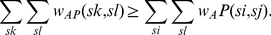
(23)


This constraint also encompasses the additional requirement of each non-local contact to be a part of exactly one “interlock”, also observed previously in literature [55].

The advantage of creating an integer linear optimization based model is the facility to create of a rank-ordered list of solutions. We aim to predict a small subset of topologies for each protein. In a number of cases, the objective function value of two topologies are highly similar to each other. By enlisting a small subset of top solutions, it enables us to differentiate between the topologies using a more detailed force field at the final stage. This can be achieved through the introduction of integer cuts. Since we are fixing the anchor points for contacts between two strands, the integer cuts would not involve the residue specific binary variables 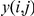. At each iteration, the addition of an integer cut eliminates the current top solution from the feasible set, thus forcing the model to look for the next best solution. We divide the set of strand-strand binary variables into two subsets: 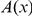 defines the subset of variables 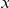 which are assigned value 1, while 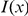 comprises of all contacts which were not active. Let 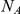 be the cardinality of the subset 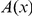. The index 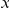 runs over all antiparallel and parallel contacts between strands. The integer cut constraint can be written as:
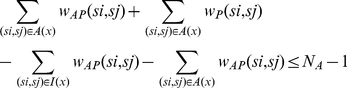
(24)


Since the objective is to maximize the contact potential between strands, most solutions would be cyclic in nature. Given that the fraction of proteins which form 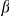 barrels is much smaller than proteins which do not, we choose to eliminate the possibility of all barrel-like structures (since about 4.7% of all proteins with subsequences in extended conformations have a barrel-like structure). Given the exponentially large number of cyclic, or sub-cyclic, solutions that are possible for a fixed number of strands, we do not add constraints to eliminate all of them up front. Instead, we check each solution for circular tours and sub-tours, and eliminate them from the feasible space using integer cuts added at each iteration. The algorithmic details of the implementation and detailed results have been presented in the Text S1. Furthermore, a detailed analysis of the constraints which provide statistical evidence of the validity of each set of constraints is provided in the Text S1.

Since the prediction of strand pairings forms a set of unordered pairs of integers, the verification of a set of basic biological consistencies is rendered difficult. One of the primary features of observed 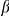-sheet topologies is the consistency of contact type along any given face of a 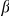-strand (i.e. all contacts of a given 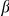-strand along one of its two faces are either antiparallel or parallel) [36]. In order to ensure that a consistent assignment is possible for a given topological prediction, each predicted topology is checked for two-colorability (i.e. we check if the predicted 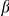-sheet topology can be re-drawn as a two-colorable graph) [36]. To do this, all contacts between strands in the predicted topology are re-cast as nodes of a graph. Two “nodes” are connected if the corresponding contacts share a 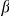-strand. In addition, the two contacts should either be of opposing natures (i.e. one should be parallel, and the second antiparallel) or they should share at least one amino acid of the common strand. The two-colorability of a graph is a well established problem, and can be solved by a breadth-first search algorithm. At the end of the algorithm, a large number of predicted sheet topologies for the target protein are received, which are ranked by the total strand-to-strand contact potential defined previously. However, in a number of cases, it was observed that the difference between the objective function values of the top few solutions was extremely low, perhaps falling into error tolerance limits. Hence, it becomes important to provide an improved ranking of the predicted sheet topologies using a detailed, atomistic level approach. Hence, we have developed a re-ranking strategy based on torsion angle dynamics and clustering, which would identify the top set of predicted topologies.

While a number of algorithms for the prediction of feasible structures satisfying a sparse set of distance and dihedral angle constraints have been presented in the literature [56], [57], torsion angle dynamics provide a very attractive alternative. Unlike classical molecular dynamics simulations, torsion angle dynamics algorithms combine steric-based energy terms with constraint violation based penalty expressions, thus allowing for faster calculations. Moreover, the primary idea moves from energy minimization to identification of feasible structures. For our algorithm, the CYANA package [58] proves to be a very useful tool for carrying out torsion angle dynamics simulations. For each predicted sheet topology, the predicted residue-to-residue contacts are converted into lower and upper bounding distance constraints, by using a small error tolerance on the hydrogen bond that would be formed between contacting amino acids. These sets of bounds, along with dihedral angle bounds on the amino acids in the 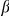-strands restricting them to the correct region of the Ramachandran plot, are provided as input to the torsion angle dynamics package. Using CYANA, we generate 200 feasible structures for each predicted sheet topology.

In order to separate out the topologies from each other, we need to assemble a small subset of representative structures from each predicted topology. To this end, we use a traveling salesman problem based clustering algorithm, ICON [59]–[62]. Here, each feasible structure generated by CYANA is considered as a node on a traveling salesman path. The problem is then reduced to one of identifying the globally optimal path to navigate through each of the “nodes”. Once such a path is established, it is partitioned into clusters such that the resulting clusters minimize the global sum of intra-cluster errors.

The computational time for the algorithm depends on the number of strands in a protein, and on the number of amino acids in the 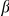-strands of the protein. For a typical eight strand protein, the mixed-integer linear optimization formulation for the prediction of 100 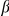-sheet topologies takes 5 minutes. The re-ranking algorithm involving torsion angle dynamics and clustering requires 10 minutes per topology to generate 200 structures. When implemented in parallel on a cluster of nodes, the entire set of topologies can be handled faster, depending on the number of processors available.

## Supporting Information

Figure S1
**Complete flowsheet of the** 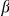 **-sheet topology prediction algorithm.**
(PDF)

Figure S2 **Graph showing the distribution of proteins in the PDBSelect25 data set versus the number of strands.**
(PDF)

Figure S3 **PDBSelect25 Data set results, classified by number of strands.**
(PDF)

Figure S4
**PDBSelect25 Data set results, differentiated between** 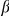 **and mixed** 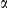 **/** 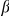 **proteins.**
(PDF)

Table S1 The number of motifs possible for a protein with n strands.
(PDF)

Table S2 The distribution of the number of proteins in the blind target set of CASP8 and CASP9 with strands.
(PDF)

Text S1
**Analysis of number of possible** 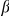 **-sheets, elimination of circular paths and constraint statistics.**
(PDF)
