# Supplementary figures and images for: β-sheet Topology Prediction with High Precision and Recall for β and Mixed α/β Proteins
Source: PLoS One. 2012 Mar 9;7(3):e32461. doi: 10.1371/journal.pone.0032461 (PMC3302896; doi:10.1371/journal.pone.0032461)

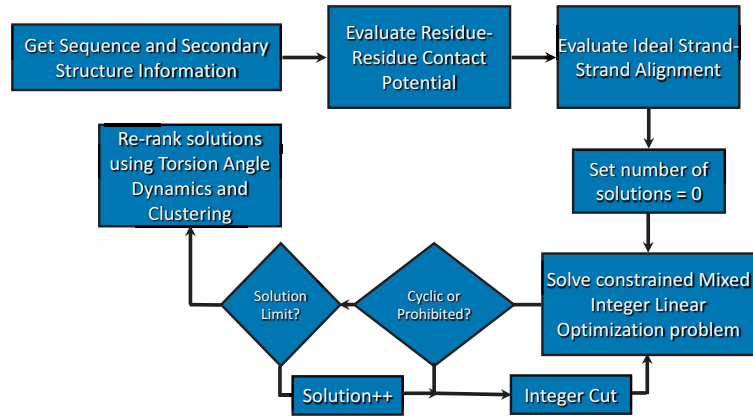

Supplement: Figure S1 — Complete flowsheet of the -sheet topology prediction algorithm. (PDF) [file pone.0032461.s001.pdf]

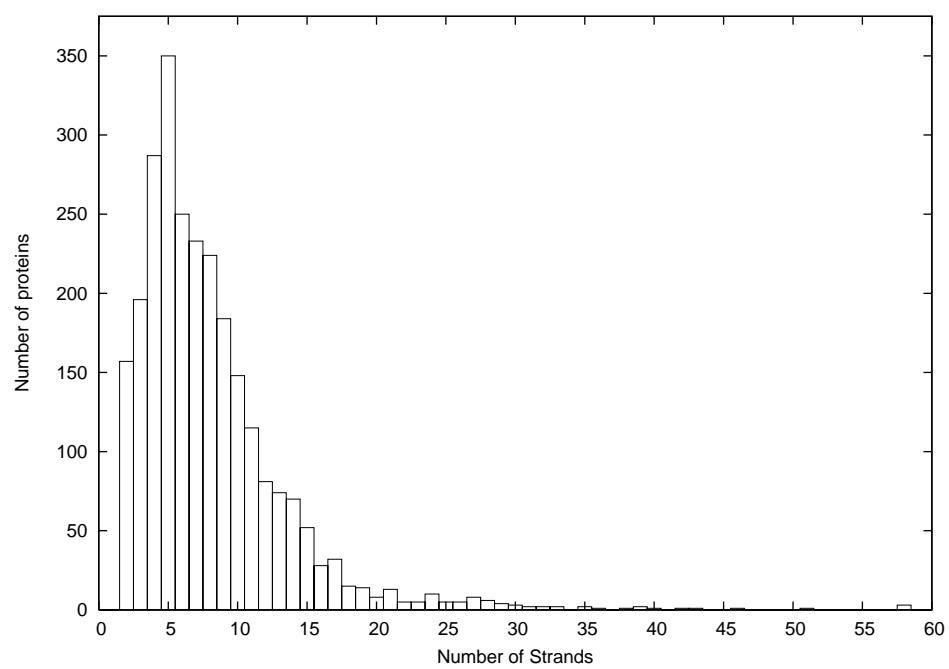

Supplement: Figure S2 — Graph showing the distribution of proteins in the PDBSelect25 data set versus the number of strands. (PDF) [file pone.0032461.s002.pdf]

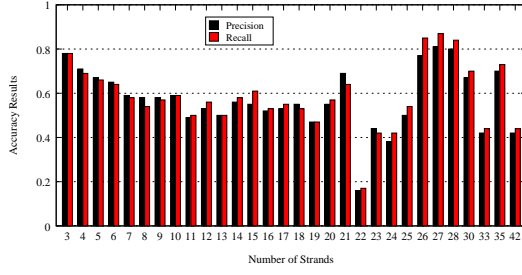

(a) Top 1 Solution

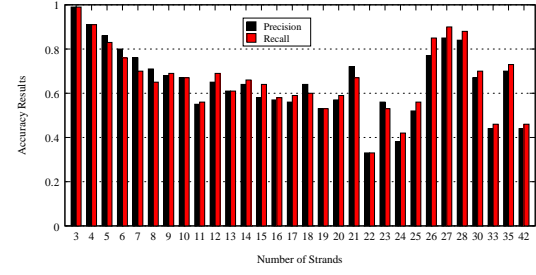

(b) Top 5 Solutions

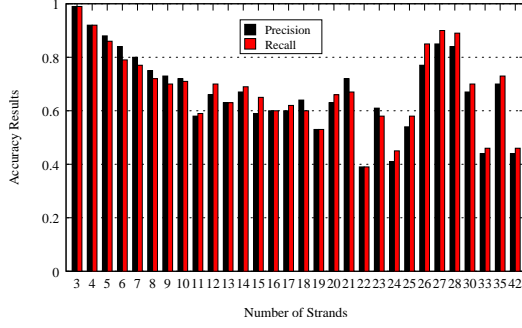

(c) Top 10 solutions

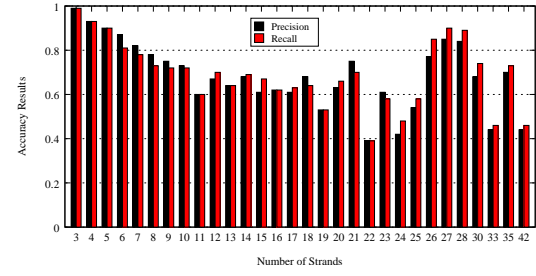

(d) Top 15 solutions

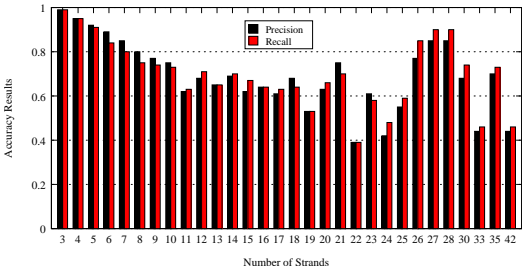

(e) Top 20 solutions

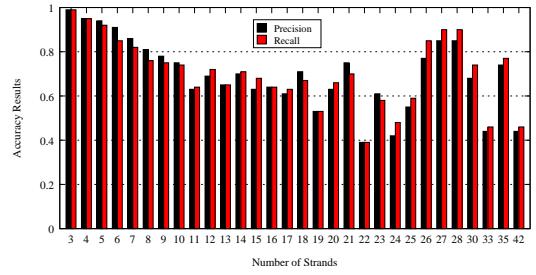

(f) Top 25 solutions

Supplement: Figure S3 — PDBSelect25 Data set results, classified by number of strands. (PDF) [file pone.0032461.s003.pdf]

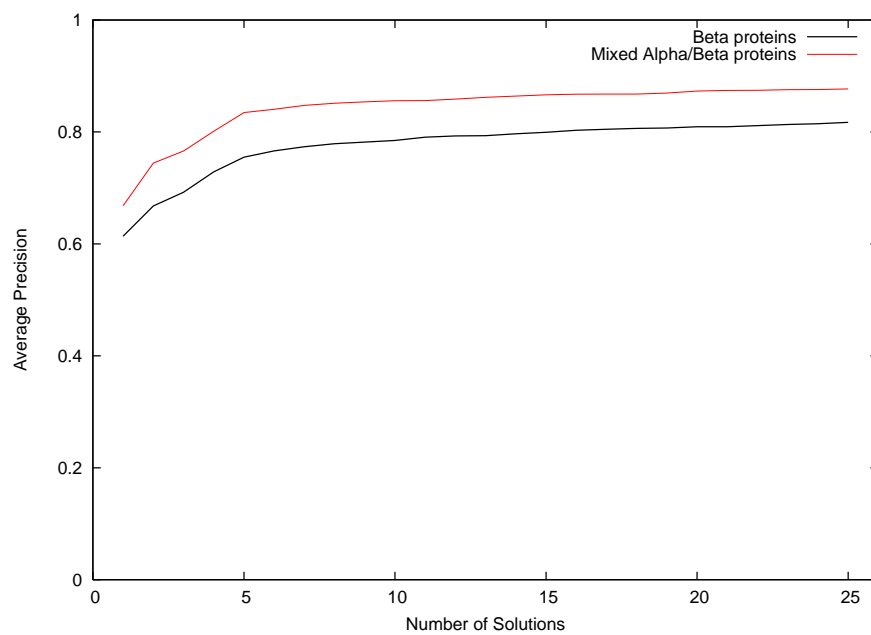

(a) Average Precision Performance

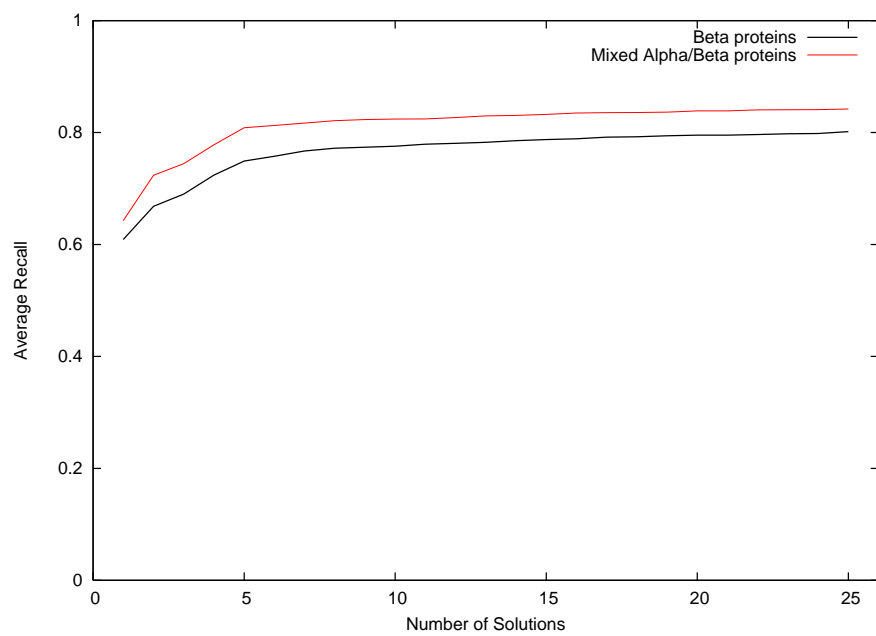

(b) Average Recall Performance

Supplement: Figure S4 — PDBSelect25 Data set results, differentiated between and mixed / proteins. (PDF) [file pone.0032461.s004.pdf]
