# Supplementary material for: β-sheet Topology Prediction with High Precision and Recall for β and Mixed α/β Proteins
Source: PLoS One. 2012 Mar 9;7(3):e32461. doi: 10.1371/journal.pone.0032461 (PMC3302896; doi:10.1371/journal.pone.0032461)
Supplement: Table S1 — The number of motifs possible for a protein with n strands. (PDF) [file pone.0032461.s005.pdf]

**Strands**	**Number of Motifs**	**Strands**	**Number of Motifs**
2	2	3	12
4	96	5	960
6	11520	7	161280
8	2580480	9	46448640
10	928972800	11	$2.0437X10^{10}$
