# Supplementary material for: β-sheet Topology Prediction with High Precision and Recall for β and Mixed α/β Proteins
Source: PLoS One. 2012 Mar 9;7(3):e32461. doi: 10.1371/journal.pone.0032461 (PMC3302896; doi:10.1371/journal.pone.0032461)
Supplement: Table S2 — The distribution of the number of proteins in the blind target set of CASP8 and CASP9 with strands. (PDF) [file pone.0032461.s006.pdf]

**Protein**	**Strands**	**Top Solution**			**Top 5 solutions**		
		Precision	Recall	MCC	Precision	Recall	MCC
3gcc	3	1.0	1.0	1.0	1.0	1.0	1.0
3ctr	3	1.0	1.0	1.0	1.0	1.0	1.0
3cjs	3	1.0	1.0	1.0	1.0	1.0	1.0
3cjs	3	1.0	1.0	1.0	1.0	1.0	1.0
2yx5	3	1.0	1.0	1.0	1.0	1.0	1.0
2yvr	3	1.0	1.0	1.0	1.0	1.0	1.0
2vb1	3	1.0	1.0	1.0	1.0	1.0	1.0
2ra4	3	1.0	1.0	1.0	1.0	1.0	1.0
2pxg	3	1.0	1.0	1.0	1.0	1.0	1.0
2p09	3	1.0	1.0	1.0	1.0	1.0	1.0
2ovg	3	1.0	1.0	1.0	1.0	1.0	1.0
2ooy	3	1.0	1.0	1.0	1.0	1.0	1.0
2odk	3	1.0	1.0	1.0	1.0	1.0	1.0
2ob9	3	1.0	1.0	1.0	1.0	1.0	1.0
2ns0	3	1.0	1.0	1.0	1.0	1.0	1.0
2k7i	3	1.0	1.0	1.0	1.0	1.0	1.0
2k4x	3	1.0	1.0	1.0	1.0	1.0	1.0
2k3i	3	1.0	1.0	1.0	1.0	1.0	1.0
2jrb	3	1.0	1.0	1.0	1.0	1.0	1.0
2jra	3	1.0	1.0	1.0	1.0	1.0	1.0
2jmp	3	1.0	1.0	1.0	1.0	1.0	1.0
2j7z	3	1.0	1.0	1.0	1.0	1.0	1.0
2iwl	3	1.0	1.0	1.0	1.0	1.0	1.0
2ho2	3	1.0	1.0	1.0	1.0	1.0	1.0

2hjj	3	1.0	1.0	1.0	1.0	1.0	1.0
2hdl	3	1.0	1.0	1.0	1.0	1.0	1.0
2hc5	3	1.0	1.0	1.0	1.0	1.0	1.0
2jtb	3	1.0	1.0	1.0	1.0	1.0	1.0
2jyv	3	1.0	1.0	1.0	1.0	1.0	1.0
2fv4	3	1.0	1.0	1.0	1.0	1.0	1.0
2fmr	3	1.0	1.0	1.0	1.0	1.0	1.0
2fgg	3	1.0	1.0	1.0	1.0	1.0	1.0
2f91	3	1.0	1.0	1.0	1.0	1.0	1.0
2el8	3	1.0	1.0	1.0	1.0	1.0	1.0
2e5r	3	1.0	1.0	1.0	1.0	1.0	1.0
2dy8	3	1.0	1.0	1.0	1.0	1.0	1.0
2dsx	3	1.0	1.0	1.0	1.0	1.0	1.0
2db2	3	1.0	1.0	1.0	1.0	1.0	1.0
2csk	3	1.0	1.0	1.0	1.0	1.0	1.0
2cs7	3	1.0	1.0	1.0	1.0	1.0	1.0
2cou	3	1.0	1.0	1.0	1.0	1.0	1.0
2cc6	3	1.0	1.0	1.0	1.0	1.0	1.0
2bh1	3	1.0	1.0	1.0	1.0	1.0	1.0
2b7t	3	1.0	1.0	1.0	1.0	1.0	1.0
2ayj	3	1.0	1.0	1.0	1.0	1.0	1.0
2aj6	3	1.0	1.0	1.0	1.0	1.0	1.0
1zzk	3	1.0	1.0	1.0	1.0	1.0	1.0
1zmq	3	1.0	1.0	1.0	1.0	1.0	1.0
1zmm	3	1.0	1.0	1.0	1.0	1.0	1.0
1yyv	3	1.0	1.0	1.0	1.0	1.0	1.0

1ywl	3	1.0	1.0	1.0	1.0	1.0	1.0
1ytf	3	1.0	1.0	1.0	1.0	1.0	1.0
1y0n	3	1.0	1.0	1.0	1.0	1.0	1.0
1xs3	3	1.0	1.0	1.0	1.0	1.0	1.0
1xjs	3	1.0	1.0	1.0	1.0	1.0	1.0
1xdx	3	1.0	1.0	1.0	1.0	1.0	1.0
1x4m	3	1.0	1.0	1.0	1.0	1.0	1.0
1x49	3	1.0	1.0	1.0	1.0	1.0	1.0
1x48	3	1.0	1.0	1.0	1.0	1.0	1.0
1wmv	3	1.0	1.0	1.0	1.0	1.0	1.0
1wlo	3	1.0	1.0	1.0	1.0	1.0	1.0
1wjw	3	1.0	1.0	1.0	1.0	1.0	1.0
1whq	3	1.0	1.0	1.0	1.0	1.0	1.0
1vqo	3	1.0	1.0	1.0	1.0	1.0	1.0
1uvg	3	1.0	1.0	1.0	1.0	1.0	1.0
1ul4	3	1.0	1.0	1.0	1.0	1.0	1.0
1uhm	3	1.0	1.0	1.0	1.0	1.0	1.0
1ub1	3	1.0	1.0	1.0	1.0	1.0	1.0
1th5	3	1.0	1.0	1.0	1.0	1.0	1.0
1t92	3	1.0	1.0	1.0	1.0	1.0	1.0
1t4n	3	1.0	1.0	1.0	1.0	1.0	1.0
1srq	3	1.0	1.0	1.0	1.0	1.0	1.0
1rod	3	1.0	1.0	1.0	1.0	1.0	1.0
1ri9	3	1.0	1.0	1.0	1.0	1.0	1.0
1r0r	3	1.0	1.0	1.0	1.0	1.0	1.0
1qkl	3	1.0	1.0	1.0	1.0	1.0	1.0

1pug	3	1.0	1.0	1.0	1.0	1.0	1.0
1ptq	3	1.0	1.0	1.0	1.0	1.0	1.0
1pij	3	1.0	1.0	1.0	1.0	1.0	1.0
1ovy	3	1.0	1.0	1.0	1.0	1.0	1.0
1nh2	3	1.0	1.0	1.0	1.0	1.0	1.0
1ncs	3	1.0	1.0	1.0	1.0	1.0	1.0
1mkn	3	1.0	1.0	1.0	1.0	1.0	1.0
1ln0	3	1.0	1.0	1.0	1.0	1.0	1.0
1kjk	3	1.0	1.0	1.0	1.0	1.0	1.0
1kbe	3	1.0	1.0	1.0	1.0	1.0	1.0
1jkz	3	1.0	1.0	1.0	1.0	1.0	1.0
1j27	3	1.0	1.0	1.0	1.0	1.0	1.0
1hy9	3	1.0	1.0	1.0	1.0	1.0	1.0
1gyx	3	1.0	1.0	1.0	1.0	1.0	1.0
1g6z	3	1.0	1.0	1.0	1.0	1.0	1.0
1g2r	3	1.0	1.0	1.0	1.0	1.0	1.0
1g1z	3	1.0	1.0	1.0	1.0	1.0	1.0
1dtj	3	1.0	1.0	1.0	1.0	1.0	1.0
1dp7	3	1.0	1.0	1.0	1.0	1.0	1.0
1dfn	3	1.0	1.0	1.0	1.0	1.0	1.0
1dfe	3	1.0	1.0	1.0	1.0	1.0	1.0
1co4	3	1.0	1.0	1.0	1.0	1.0	1.0
1c6w	3	1.0	1.0	1.0	1.0	1.0	1.0
1bb8	3	1.0	1.0	1.0	1.0	1.0	1.0
1apf	3	1.0	1.0	1.0	1.0	1.0	1.0
1ab7	3	1.0	1.0	1.0	1.0	1.0	1.0

4sgb	3	0.5	0.5	-0.5	1.0	1.0	1.0
2p09	3	1.0	1.0	1.0	1.0	1.0	1.0
3egr	3	0.5	0.5	-0.5	1.0	1.0	1.0
3dpl	3	0.5	0.5	-0.5	1.0	1.0	1.0
3dom	3	0.5	0.5	-0.5	1.0	1.0	1.0
3dgp	3	0.5	0.5	-0.5	1.0	1.0	1.0
3ddt	3	0.5	0.5	-0.5	1.0	1.0	1.0
3bsu	3	0.5	0.5	0	1.0	1.0	1.0
2vqc	3	0.5	0.5	-0.5	1.0	1.0	1.0
2v1n	3	0.5	0.5	-0.5	1.0	1.0	1.0
2qvo	3	0.5	0.5	-0.5	1.0	1.0	1.0
2pst	3	0.5	0.5	0	1.0	1.0	1.0
2ppi	3	0.5	0.5	-0.5	1.0	1.0	1.0
2od5	3	0.5	0.5	-0.5	1.0	1.0	1.0
2k2d	3	0.5	0.5	-0.5	1.0	1.0	1.0
2jtv	3	0.5	0.5	-0.5	1.0	1.0	1.0
2jt1	3	0.5	0.5	-0.5	1.0	1.0	1.0
2jp6	3	0.5	0.5	-0.5	1.0	1.0	1.0
2jnj	3	0.5	0.5	-0.5	1.0	1.0	1.0
2ikd	3	0.5	0.5	0	1.0	1.0	1.0
2hvf	3	0.5	0.5	-0.5	1.0	1.0	1.0
2hh3	3	0.5	0.5	0	1.0	1.0	1.0
2hdp	3	0.5	0.5	-0.5	1.0	1.0	1.0
2guk	3	0.5	0.5	-0.5	1.0	1.0	1.0
2fqh	3	0.5	0.5	0	1.0	1.0	1.0
2fma	3	0.5	0.5	-0.5	1.0	1.0	1.0

2dy7	3	0.5	0.5	0	1.0	1.0	1.0
2dk5	3	0.5	0.5	-0.5	1.0	1.0	1.0
2did	3	0.5	0.5	0	1.0	1.0	1.0
2d8v	3	0.5	0.5	-0.5	1.0	1.0	1.0
2d7h	3	0.5	0.5	-0.5	1.0	1.0	1.0
2d28	3	0.5	0.5	0	1.0	1.0	1.0
2cpm	3	0.5	0.5	-0.5	1.0	1.0	1.0
2cka	3	0.5	0.5	0	1.0	1.0	1.0
2bay	3	0.5	0.5	-0.5	1.0	1.0	1.0
1yd6	3	0.5	0.5	-0.5	1.0	1.0	1.0
1xmk	3	0.5	0.5	-0.5	1.0	1.0	1.0
1xma	3	0.5	0.5	-0.5	1.0	1.0	1.0
1xhj	3	0.5	0.5	0	1.0	1.0	1.0
1x5v	3	0.5	0.5	-0.5	1.0	1.0	1.0
1wi9	3	0.5	0.5	-0.5	1.0	1.0	1.0
1wgm	3	0.5	0.5	-0.5	1.0	1.0	1.0
1wf9	3	0.5	0.5	0	1.0	1.0	1.0
1w6v	3	0.5	0.5	-0.5	1.0	1.0	1.0
1w1w	3	0.5	0.5	-0.5	1.0	1.0	1.0
1vnb	3	0.5	0.5	-0.5	1.0	1.0	1.0
1vbw	3	0.5	0.5	0	1.0	1.0	1.0
1ufm	3	0.5	0.5	-0.5	1.0	1.0	1.0
1tv0	3	0.5	0.5	-0.5	1.0	1.0	1.0
1tsk	3	0.5	0.5	-0.5	1.0	1.0	1.0
1ti5	3	0.5	0.5	-0.5	1.0	1.0	1.0
1t2t	3	0.5	0.5	-0.5	1.0	1.0	1.0

1t1h	3	0.5	0.5	-0.5	1.0	1.0	1.0
1smb	3	0.5	0.5	-0.5	1.0	1.0	1.0
1sis	3	0.5	0.5	-0.5	1.0	1.0	1.0
1sen	3	0.5	0.5	-0.5	1.0	1.0	1.0
1sb6	3	0.5	0.5	-0.5	1.0	1.0	1.0
1r29	3	0.5	0.5	-0.5	1.0	1.0	1.0
1pba	3	0.5	0.5	-0.5	1.0	1.0	1.0
1pav	3	0.5	0.5	-0.5	1.0	1.0	1.0
1p9g	3	0.5	0.5	-0.5	1.0	1.0	1.0
1p8b	3	0.5	0.5	-0.5	1.0	1.0	1.0
1p0a	3	0.5	0.5	-0.5	1.0	1.0	1.0
1ocy	3	0.5	0.5	-0.5	1.0	1.0	1.0
1ny8	3	0.5	0.5	0	1.0	1.0	1.0
1n4n	3	0.5	0.5	-0.5	1.0	1.0	1.0
1msz	3	0.5	0.5	-0.5	1.0	1.0	1.0
1lr0	3	0.5	0.5	-0.5	1.0	1.0	1.0
1kvd	3	0.5	0.5	-0.5	1.0	1.0	1.0
1kqh	3	0.5	0.5	-0.5	1.0	1.0	1.0
1kj5	3	0.5	0.5	-0.5	1.0	1.0	1.0
1jxs	3	0.5	0.5	-0.5	1.0	1.0	1.0
1jid	3	0.5	0.5	-0.5	1.0	1.0	1.0
1j75	3	0.5	0.5	-0.5	1.0	1.0	1.0
1ix9	3	0.5	0.5	-0.5	1.0	1.0	1.0
1iw4	3	0.5	0.5	-0.5	1.0	1.0	1.0
1iuy	3	0.5	0.5	-0.5	1.0	1.0	1.0
1iua	3	0.5	0.5	-0.5	1.0	1.0	1.0

1hx7	3	0.5	0.5	-0.5	1.0	1.0	1.0
1g25	3	0.5	0.5	0	1.0	1.0	1.0
1ews	3	0.5	0.5	-0.5	1.0	1.0	1.0
1eod	3	0.5	0.5	-0.5	1.0	1.0	1.0
1dkc	3	0.5	0.5	-0.5	1.0	1.0	1.0
1cf7	3	0.5	0.5	-0.5	1.0	1.0	1.0
1brz	3	0.5	0.5	-0.5	1.0	1.0	1.0
1boe	3	0.5	0.5	-0.5	1.0	1.0	1.0
3grx	4	1.0	1.0	1.0	1.0	1.0	1.0
3ci0	4	1.0	1.0	1.0	1.0	1.0	1.0
3ca7	4	1.0	1.0	1.0	1.0	1.0	1.0
3c9b	4	1.0	1.0	1.0	1.0	1.0	1.0
3b47	4	1.0	1.0	1.0	1.0	1.0	1.0
2z6o	4	1.0	1.0	1.0	1.0	1.0	1.0
2yxy	4	1.0	1.0	1.0	1.0	1.0	1.0
2ywq	4	1.0	1.0	1.0	1.0	0.67	0.71
2s0y	4	1.0	1.0	1.0	1.0	1.0	1.0
2s04	4	1.0	1.0	1.0	1.0	1.0	1.0
2qsk	4	1.0	1.0	1.0	1.0	1.0	1.0
2qkh	4	1.0	1.0	1.0	1.0	1.0	1.0
2k5c	4	1.0	1.0	1.0	1.0	1.0	1.0
2k2w	4	1.0	1.0	1.0	1.0	1.0	1.0
2k1h	4	1.0	1.0	1.0	1.0	1.0	1.0
2jv8	4	1.0	1.0	1.0	1.0	1.0	1.0
2jrj	4	1.0	1.0	1.0	1.0	1.0	1.0
2jor	4	1.0	1.0	1.0	1.0	1.0	1.0

2jod	4	1.0	1.0	1.0	1.0	1.0	1.0
2j3r	4	1.0	1.0	1.0	1.0	1.0	1.0
2j03	4	1.0	1.0	1.0	1.0	1.0	1.0
2iy2	4	1.0	1.0	1.0	1.0	1.0	1.0
2hx2	4	1.0	1.0	1.0	1.0	1.0	1.0
2hgc	4	1.0	1.0	1.0	1.0	1.0	1.0
2hbp	4	1.0	1.0	1.0	1.0	1.0	1.0
2ftx	4	1.0	1.0	1.0	1.0	1.0	1.0
2fm7	4	1.0	1.0	1.0	1.0	1.0	1.0
2ffg	4	1.0	1.0	1.0	1.0	1.0	1.0
2fdn	4	1.0	1.0	1.0	1.0	1.0	1.0
2f4w	4	1.0	1.0	1.0	1.0	1.0	1.0
2ex5	4	1.0	1.0	1.0	1.0	1.0	1.0
2eky	4	1.0	1.0	1.0	1.0	1.0	1.0
2dko	4	1.0	1.0	1.0	1.0	1.0	1.0
2dko	4	1.0	1.0	1.0	1.0	1.0	1.0
2d5r	4	1.0	1.0	1.0	1.0	1.0	1.0
2cw1	4	1.0	1.0	1.0	1.0	1.0	1.0
2ct7	4	1.0	1.0	1.0	1.0	1.0	1.0
2cjs	4	1.0	1.0	1.0	1.0	1.0	1.0
2bx9	4	1.0	1.0	1.0	1.0	1.0	1.0
2bhm	4	1.0	1.0	1.0	1.0	1.0	1.0
2ask	4	1.0	1.0	1.0	1.0	1.0	1.0
1zk6	4	1.0	1.0	1.0	1.0	1.0	1.0
1zgu	4	1.0	1.0	1.0	1.0	1.0	1.0
1z9w	4	1.0	1.0	1.0	1.0	1.0	1.0

1z8m	4	1.0	1.0	1.0	1.0	1.0	1.0
1z2u	4	1.0	1.0	1.0	1.0	1.0	1.0
1xpv	4	1.0	0.67	0.71	1.0	1.0	1.0
1xee	4	1.0	1.0	1.0	1.0	1.0	1.0
1x6e	4	1.0	1.0	1.0	1.0	1.0	1.0
1x4s	4	1.0	1.0	1.0	1.0	1.0	1.0
1x4l	4	1.0	1.0	1.0	1.0	1.0	1.0
1wpu	4	1.0	1.0	1.0	1.0	1.0	1.0
1wh2	4	1.0	1.0	1.0	1.0	1.0	1.0
1vfy	4	1.0	1.0	1.0	1.0	1.0	1.0
1vaz	4	1.0	1.0	1.0	1.0	1.0	1.0
1v5i	4	1.0	1.0	1.0	1.0	1.0	1.0
1v14	4	1.0	1.0	1.0	1.0	1.0	1.0
1usm	4	1.0	1.0	1.0	1.0	1.0	1.0
1twc	4	1.0	1.0	1.0	1.0	1.0	1.0
1tfi	4	1.0	1.0	1.0	1.0	1.0	1.0
1t9i	4	1.0	1.0	1.0	1.0	1.0	1.0
1t6r	4	1.0	1.0	1.0	1.0	1.0	1.0
1sro	4	1.0	1.0	1.0	1.0	1.0	1.0
1shi	4	1.0	1.0	1.0	1.0	1.0	1.0
1s0y	4	1.0	1.0	1.0	1.0	1.0	1.0
1s04	4	1.0	1.0	1.0	1.0	1.0	1.0
1pyo	4	1.0	1.0	1.0	1.0	1.0	1.0
1pcf	4	1.0	1.0	1.0	1.0	1.0	1.0
1lmm	4	1.0	1.0	1.0	1.0	1.0	1.0
1lkk	4	1.0	1.0	1.0	1.0	1.0	1.0

1kwi	4	1.0	1.0	1.0	1.0	1.0	1.0
1kp6	4	1.0	1.0	1.0	1.0	1.0	1.0
1jrm	4	1.0	1.0	1.0	1.0	1.0	1.0
1j2l	4	1.0	1.0	1.0	1.0	1.0	1.0
1j27	4	1.0	1.0	1.0	1.0	1.0	1.0
1j0p	4	1.0	1.0	1.0	1.0	1.0	1.0
1guo	4	1.0	1.0	1.0	1.0	1.0	1.0
1g5v	4	1.0	1.0	1.0	1.0	1.0	1.0
1fsh	4	1.0	1.0	1.0	1.0	1.0	1.0
1fsb	4	1.0	1.0	1.0	1.0	1.0	1.0
1faq	4	1.0	1.0	1.0	1.0	1.0	1.0
1dec	4	1.0	1.0	1.0	1.0	1.0	1.0
1bf9	4	1.0	1.0	1.0	1.0	1.0	1.0
1auu	4	1.0	1.0	1.0	1.0	1.0	1.0
1x4l	4	1.0	1.0	1.0	1.0	1.0	1.0
1apq	4	1.0	1.0	1.0	1.0	1.0	1.0
3ci0	4	1.0	1.0	1.0	1.0	1.0	1.0
3grx	4	1.0	1.0	1.0	1.0	1.0	1.0
1hx2	4	1.0	1.0	1.0	1.0	1.0	1.0
3dwv	4	0.67	0.67	0.33	1.0	1.0	1.0
3dfe	4	0.67	0.67	0.33	1.0	1.0	1.0
3df8	4	0.67	1.0	0.71	1.0	1.0	1.0
3ct6	4	0.67	0.67	0.33	1.0	1.0	1.0
3ccd	4	0.67	0.67	0.33	1.0	1.0	1.0
3bv8	4	0.67	0.67	0.33	1.0	1.0	1.0
3b8o	4	0.67	0.67	0.33	1.0	1.0	1.0

2yz0	4	0.67	0.67	0.42	1.0	1.0	1.0
2yre	4	0.67	1.0	0.71	1.0	1.0	1.0
2vkc	4	0.67	0.67	0.33	1.0	1.0	1.0
2vgh	4	0.67	1.0	0.71	1.0	1.0	1.0
2va0	4	0.67	0.67	0.33	1.0	1.0	1.0
2v6v	4	0.67	0.67	0.33	1.0	1.0	1.0
2rfk	4	0.67	0.67	0.33	1.0	1.0	1.0
2qmt	4	0.67	0.67	0.42	1.0	1.0	1.0
2qif	4	0.67	0.67	0.33	1.0	1.0	1.0
2pv1	4	0.67	0.67	0.33	1.0	1.0	1.0
2pr3	4	0.67	1.0	0.71	1.0	1.0	1.0
2p6t	4	0.67	0.67	0.33	1.0	1.0	1.0
2ov6	4	0.67	0.67	0.42	1.0	1.0	1.0
2osd	4	0.67	0.67	0.33	1.0	1.0	1.0
2o1r	4	0.67	0.67	0.33	1.0	1.0	1.0
2nuh	4	0.67	0.67	0.33	0.67	0.67	0.33
2nto	4	0.67	0.67	0.33	1.0	0.67	0.71
2jro	4	0.67	0.67	0.42	1.0	1.0	1.0
2jq5	4	0.67	0.67	0.33	1.0	1.0	1.0
2iyb	4	0.67	1.0	0.71	1.0	1.0	1.0
2ida	4	0.67	0.67	0.42	1.0	1.0	1.0
2hx6	4	0.67	0.67	0.33	1.0	1.0	1.0
2ht9	4	0.67	0.67	0.33	1.0	1.0	1.0
2hj8	4	0.67	0.67	0.42	1.0	1.0	1.0
2hd0	4	0.67	0.67	0.33	0.67	0.67	0.33
2h0u	4	0.67	0.67	0.42	1.0	1.0	1.0

2gi4	4	0.67	0.67	0.33	1.0	0.67	0.71
2g2q	4	0.67	0.67	0.33	1.0	1.0	1.0
2fqm	4	0.67	1.0	0.71	0.67	1.0	0.71
2fgx	4	0.67	0.67	0.33	1.0	0.67	0.71
2fcw	4	0.67	1.0	0.71	1.0	1.0	1.0
2ec4	4	0.67	0.67	0.33	1.0	0.67	0.71
2e0g	4	0.67	0.67	0.33	0.67	0.67	0.33
2dmm	4	0.67	0.67	0.42	0.67	0.67	0.33
2dao	4	0.67	0.67	0.42	1.0	1.0	1.0
2d8m	4	0.67	0.67	0.33	0.67	0.67	0.33
2cfx	4	0.67	0.67	0.33	1.0	1.0	1.0
2bkn	4	0.67	0.67	0.33	1.0	1.0	1.0
2b9k	4	0.67	0.67	0.33	1.0	1.0	1.0
2b3w	4	0.67	1.0	0.71	0.67	1.0	0.71
1z3e	4	0.67	0.67	0.33	1.0	0.67	0.71
1xww	4	0.67	0.67	0.33	1.0	0.67	0.71
1xpp	4	0.67	0.67	0.33	1.0	0.67	0.71
1xi7	4	0.67	0.67	0.33	1.0	1.0	1.0
1xg8	4	0.67	0.67	0.33	1.0	0.67	0.71
1x9a	4	0.67	0.67	0.42	0.67	0.67	0.33
1x8d	4	0.67	0.67	0.33	1.0	1.0	1.0
1x7v	4	0.67	0.67	0.33	1.0	0.67	0.71
1x60	4	0.67	0.67	0.33	1.0	1.0	1.0
1wxn	4	0.67	0.67	0.33	1.0	1.0	1.0
1wwq	4	0.67	0.67	0.33	1.0	1.0	1.0
1wpi	4	0.67	0.67	0.33	1.0	0.67	0.71

1wjv	4	0.67	0.67	0.33	1.0	1.0	1.0
1wik	4	0.67	0.67	0.33	1.0	0.67	0.71
1whx	4	0.67	0.67	0.33	1.0	1.0	1.0
1v9x	4	0.67	0.67	0.33	0.67	0.67	0.42
1uzx	4	0.67	0.67	0.33	0.67	0.67	0.33
1unn	4	0.67	0.67	0.33	1.0	0.67	0.71
1ul7	4	0.67	0.67	0.33	1.0	0.67	0.71
1u2f	4	0.67	0.67	0.33	0.67	0.67	0.33
1txe	4	0.67	0.67	0.33	1.0	1.0	1.0
1ti8	4	0.67	1.0	0.71	1.0	1.0	1.0
1te7	4	0.67	0.67	0.33	1.0	1.0	1.0
1t4z	4	0.67	0.67	0.33	1.0	0.67	0.71
1t1v	4	0.67	0.67	0.33	1.0	0.67	0.71
1t0h	4	0.67	0.67	0.42	1.0	1.0	1.0
1sj1	4	0.67	1.0	0.71	0.67	1.0	0.71
1s3a	4	0.67	0.67	0.42	0.67	0.67	0.33
1rw1	4	0.67	0.67	0.33	1.0	0.67	0.71
1rmd	4	0.67	1.0	0.71	0.67	1.0	0.71
1rjj	4	0.67	0.67	0.33	1.0	1.0	1.0
1r4w	4	0.67	0.67	0.33	1.0	0.67	0.71
1qyn	4	0.67	0.67	0.33	1.0	0.67	0.71
1qld	4	0.67	1.0	0.71	1.0	1.0	1.0
1q5y	4	0.67	0.67	0.33	1.0	0.67	0.71
1p9k	4	0.67	0.67	0.42	0.67	0.67	0.42
1o2f	4	0.67	0.67	0.33	1.0	1.0	1.0
1n88	4	0.67	0.67	0.42	1.0	1.0	1.0

1n32	4	0.67	1.0	0.71	0.67	1.0	0.71
1n32	4	0.67	1.0	0.71	0.67	1.0	0.71
1n32	4	0.67	1.0	0.71	0.67	1.0	0.71
1n32	4	0.67	1.0	0.71	0.67	1.0	0.71
1mvf	4	0.67	1.0	0.71	0.67	1.0	0.71
1lxj	4	0.67	0.67	0.33	1.0	1.0	1.0
1ka8	4	0.67	1.0	0.71	0.67	1.0	0.71
1k4u	4	0.67	0.67	0.42	1.0	1.0	1.0
1jo0	4	0.67	0.67	0.33	1.0	1.0	1.0
1jf8	4	0.67	0.67	0.33	1.0	0.67	0.71
1j98	4	0.67	0.67	0.33	1.0	0.67	0.71
1ism	4	0.67	0.67	0.42	0.67	0.67	0.33
1iqz	4	0.67	1.0	0.71	0.67	1.0	0.71
1hz6	4	0.67	0.67	0.33	0.67	0.67	0.33
1h75	4	0.67	0.67	0.42	0.67	0.67	0.42
1gh9	4	0.67	0.67	0.33	1.0	1.0	1.0
1g7e	4	0.67	0.67	0.33	0.67	0.67	0.42
1g47	4	0.67	1.0	0.71	1.0	1.0	1.0
1fxk	4	0.67	1.0	0.71	0.67	1.0	0.71
1fo5	4	0.67	0.67	0.33	1.0	0.67	0.71
1emw	4	0.67	0.67	0.33	1.0	1.0	1.0
1ed7	4	0.67	1.0	0.71	0.67	1.0	0.71
1eai	4	0.67	1.0	0.71	0.67	1.0	0.71
1e8p	4	0.67	0.67	0.33	1.0	1.0	1.0
1e44	4	0.67	0.67	0.33	1.0	1.0	1.0
1de1	4	0.67	0.67	0.33	1.0	0.67	0.71

1dcj	4	0.67	0.67	0.33	0.67	0.67	0.42
1cxw	4	0.67	1.0	0.71	1.0	1.0	1.0
1ccv	4	0.67	1.0	0.71	1.0	1.0	1.0
1cc8	4	0.67	0.67	0.33	1.0	1.0	1.0
1bcr	4	0.67	0.67	0.33	0.67	0.67	0.33
2ibl	4	0.5	0.5	0.3	0.5	0.5	0.3
1cou	4	0.5	0.5	0.3	1.0	1.0	1.0
3e8o	4	0.33	0.33	-0.33	1.0	1.0	1.0
3dex	4	0.33	0.33	-0.17	0.5	0.33	0.091
3d32	4	0.33	0.33	-0.067	0.67	0.67	0.42
3bm7	4	0.33	0.33	-0.33	1.0	1.0	1.0
2zbc	4	0.33	0.33	-0.33	1.0	1.0	1.0
2z30	4	0.33	0.33	-0.33	1.0	1.0	1.0
2rgv	4	0.33	0.5	0	0.5	0.5	0.3
2qt7	4	0.33	0.33	-0.33	0.67	0.67	0.33
2qsw	4	0.33	0.33	-0.33	0.67	0.67	0.33
2obb	4	0.33	0.33	-0.33	0.67	0.67	0.42
2ibo	4	0.33	0.33	-0.33	0.67	0.67	0.33
2hyi	4	0.33	0.33	-0.33	0.67	0.67	0.33
2hfq	4	0.33	0.33	-0.33	0.67	0.67	0.33
2g1e	4	0.33	0.33	-0.17	0.67	0.67	0.33
2g0j	4	0.33	0.33	-0.33	0.67	0.67	0.42
2g0c	4	0.33	0.33	-0.33	0.67	0.67	0.33
2f3j	4	0.33	0.33	-0.33	0.67	0.67	0.33
2eh1	4	0.33	0.33	-0.33	0.67	0.67	0.33
2djk	4	0.33	0.33	-0.33	0.67	0.67	0.33

2cfa	4	0.33	0.33	-0.067	1.0	1.0	1.0
2caj	4	0.33	0.33	-0.33	0.67	0.67	0.33
2b0g	4	0.33	0.33	-0.33	0.67	0.67	0.33
2aze	4	0.33	0.5	0	0.67	1.0	0.71
2aiz	4	0.33	0.33	-0.33	1.0	1.0	1.0
2a3j	4	0.33	0.33	-0.33	0.67	0.67	0.33
1yo3	4	0.33	0.33	-0.33	0.67	0.67	0.42
1y9x	4	0.33	0.33	-0.33	0.67	0.67	0.33
1xbw	4	0.33	0.33	-0.33	0.67	0.67	0.33
1xax	4	0.33	0.33	-0.17	0.5	0.33	0.091
1x65	4	0.33	0.33	-0.33	1.0	1.0	1.0
1x4f	4	0.33	0.33	-0.33	0.67	0.67	0.33
1x4e	4	0.33	0.33	-0.33	0.67	0.67	0.33
1wot	4	0.33	0.33	-0.17	1.0	0.67	0.71
1why	4	0.33	0.33	-0.33	0.67	0.67	0.33
1wg5	4	0.33	0.33	-0.33	1.0	1.0	1.0
1wf6	4	0.33	0.33	-0.067	0.33	0.33	-0.33
1wex	4	0.33	0.33	-0.33	0.67	0.67	0.33
1vrv	4	0.33	0.33	-0.17	0.67	0.67	0.42
1vr4	4	0.33	0.33	-0.33	0.67	0.67	0.33
1vqs	4	0.33	0.33	-0.33	1.0	1.0	1.0
1vmb	4	0.33	0.33	-0.33	0.67	0.67	0.33
1u3o	4	0.33	0.5	0.091	0.5	0.5	0.3
1tz1	4	0.33	0.5	0	1.0	1.0	1.0
1owx	4	0.33	0.33	-0.33	0.67	0.67	0.33
1otg	4	0.33	0.33	-0.17	0.67	0.67	0.42

1oq6	4	0.33	0.33	-0.33	1.0	1.0	1.0
1nz0	4	0.33	0.33	-0.33	1.0	1.0	1.0
1nsk	4	0.33	0.33	-0.33	1.0	1.0	1.0
1n6z	4	0.33	0.33	-0.17	1.0	1.0	1.0
1mg4	4	0.33	0.33	-0.17	0.67	0.67	0.33
1lq9	4	0.33	0.33	-0.33	1.0	1.0	1.0
1jru	4	0.33	0.33	-0.067	0.67	0.67	0.33
1jj4	4	0.33	0.33	-0.33	0.67	0.67	0.33
1iuj	4	0.33	0.33	-0.33	0.67	0.67	0.33
1ip9	4	0.33	0.5	0.091	1.0	1.0	1.0
1d9a	4	0.33	0.33	-0.33	0.67	0.67	0.33
1cse	4	0.33	0.33	-0.17	0.67	0.67	0.42
1afp	4	0.33	0.33	-0.33	1.0	1.0	1.0
3d0f	5	1.0	1.0	1.0	1.0	1.0	1.0
3b7c	5	1.0	1.0	1.0	1.0	1.0	1.0
2v0u	5	1.0	0.75	0.8	1.0	0.75	0.8
2p1j	5	1.0	0.75	0.8	1.0	0.75	0.8
2p0q	5	1.0	1.0	1.0	1.0	1.0	1.0
2ot2	5	1.0	0.8	0.82	1.0	0.8	0.82
2oik	5	1.0	1.0	1.0	1.0	0.75	0.8
2oct	5	1.0	1.0	1.0	1.0	1.0	1.0
2o9u	5	1.0	1.0	1.0	1.0	1.0	1.0
2jvr	5	1.0	1.0	1.0	1.0	1.0	1.0
2juf	5	1.0	1.0	1.0	1.0	1.0	1.0
2j3t	5	1.0	1.0	1.0	1.0	1.0	1.0
2j3t	5	1.0	1.0	1.0	1.0	1.0	1.0

2ia1	5	1.0	1.0	1.0	1.0	1.0	1.0
2hna	5	1.0	1.0	1.0	1.0	1.0	1.0
2gw6	5	1.0	1.0	1.0	1.0	1.0	1.0
2glz	5	1.0	1.0	1.0	1.0	1.0	1.0
2gjv	5	1.0	1.0	1.0	1.0	1.0	1.0
2gj3	5	1.0	1.0	1.0	1.0	0.75	0.8
2fm8	5	1.0	1.0	1.0	1.0	0.75	0.8
2drp	5	1.0	1.0	1.0	1.0	1.0	1.0
2aoa	5	1.0	1.0	1.0	1.0	1.0	1.0
1zno	5	1.0	1.0	1.0	1.0	0.75	0.8
1y4o	5	1.0	1.0	1.0	1.0	1.0	1.0
1xmt	5	1.0	1.0	1.0	1.0	1.0	1.0
1x0o	5	1.0	1.0	1.0	1.0	1.0	1.0
1v5r	5	1.0	1.0	1.0	1.0	0.75	0.8
1v1j	5	1.0	1.0	1.0	1.0	1.0	1.0
1uw0	5	1.0	1.0	1.0	1.0	1.0	1.0
1ug1	5	1.0	1.0	1.0	1.0	0.75	0.8
1u5m	5	1.0	1.0	1.0	1.0	1.0	1.0
1tot	5	1.0	1.0	1.0	1.0	1.0	1.0
1t3g	5	1.0	1.0	1.0	1.0	1.0	1.0
1sbx	5	1.0	1.0	1.0	1.0	1.0	1.0
1s28	5	1.0	1.0	1.0	1.0	0.75	0.8
1rk8	5	1.0	1.0	1.0	1.0	1.0	1.0
1nwz	5	1.0	1.0	1.0	1.0	0.75	0.8
1l8r	5	1.0	1.0	1.0	1.0	1.0	1.0
1kq1	5	1.0	1.0	1.0	1.0	1.0	1.0

1jyo	5	1.0	1.0	1.0	1.0	1.0	1.0
1jrm	5	1.0	1.0	1.0	1.0	1.0	1.0
1jc7	5	1.0	0.6	0.65	1.0	0.6	0.65
1fgp	5	1.0	1.0	1.0	1.0	1.0	1.0
1dqc	5	1.0	1.0	1.0	1.0	1.0	1.0
1dch	5	1.0	1.0	1.0	1.0	1.0	1.0
1d0q	5	1.0	1.0	1.0	1.0	0.75	0.8
1c9o	5	1.0	1.0	1.0	1.0	1.0	1.0
1bvy	5	1.0	1.0	1.0	1.0	0.75	0.8
3eoi	5	0.75	0.75	0.58	1.0	0.75	0.8
3dwm	5	0.75	0.75	0.61	1.0	0.75	0.8
3do9	5	0.75	0.75	0.61	1.0	0.75	0.8
3dnx	5	0.75	0.75	0.61	1.0	1.0	1.0
3c5k	5	0.75	0.75	0.58	1.0	1.0	1.0
3c4s	5	0.75	0.75	0.58	1.0	0.75	0.8
3bvp	5	0.75	0.75	0.58	1.0	1.0	1.0
3bn8	5	0.75	0.75	0.58	1.0	1.0	1.0
3bjk	5	0.75	0.75	0.58	1.0	1.0	1.0
3bdu	5	0.75	1.0	0.8	1.0	1.0	1.0
2zej	5	0.75	1.0	0.8	0.75	1.0	0.8
2z5v	5	0.75	0.75	0.61	1.0	1.0	1.0
2vy8	5	0.75	1.0	0.8	1.0	1.0	1.0
2vxf	5	0.75	0.75	0.58	0.75	0.75	0.58
2vlg	5	0.75	0.75	0.58	1.0	1.0	1.0
2rjz	5	0.75	0.75	0.58	1.0	1.0	1.0
2r2z	5	0.75	0.75	0.58	1.0	1.0	1.0

2qv0	5	0.75	0.75	0.61	1.0	1.0	1.0
2qtx	5	0.75	0.75	0.58	1.0	1.0	1.0
2qlv	5	0.75	0.75	0.58	1.0	1.0	1.0
2qhk	5	0.75	0.75	0.58	1.0	0.75	0.8
2qdy	5	0.75	0.75	0.61	1.0	1.0	1.0
2q3v	5	0.75	0.75	0.58	0.75	0.75	0.58
2pls	5	0.75	0.75	0.58	1.0	1.0	1.0
2pfu	5	0.75	0.75	0.61	1.0	0.75	0.8
2p92	5	0.75	0.75	0.58	0.75	0.75	0.58
2o9s	5	0.75	0.75	0.58	1.0	0.75	0.8
2o90	5	0.75	0.75	0.61	0.75	0.75	0.61
2ns5	5	0.75	0.75	0.58	1.0	1.0	1.0
2nqw	5	0.75	0.75	0.58	1.0	1.0	1.0
2k4v	5	0.75	0.75	0.58	1.0	1.0	1.0
2k3d	5	0.75	0.75	0.58	1.0	1.0	1.0
2jtm	5	0.75	1.0	0.8	1.0	1.0	1.0
2jtf	5	0.75	0.75	0.58	1.0	1.0	1.0
2jny	5	0.75	0.75	0.58	1.0	0.75	0.8
2i5h	5	0.75	0.75	0.58	1.0	1.0	1.0
2hvv	5	0.75	0.75	0.58	1.0	0.75	0.8
2gyb	5	0.75	0.75	0.61	1.0	0.75	0.8
2gkg	5	0.75	0.75	0.61	0.75	0.61	0.75
2gcx	5	0.75	0.75	0.58	1.0	1.0	1.0
2fz0	5	0.75	0.75	0.61	1.0	1.0	1.0
2fr5	5	0.75	0.75	0.61	1.0	0.75	0.8
2fpe	5	0.75	0.75	0.58	1.0	0.75	0.8

2fio	5	0.75	0.75	0.58	1.0	1.0	1.0
2fhz	5	0.75	0.75	0.58	1.0	1.0	1.0
2fhz	5	0.75	0.75	0.58	1.0	1.0	1.0
2f5k	5	0.75	0.75	0.58	1.0	1.0	1.0
2equ	5	0.75	0.75	0.58	1.0	1.0	1.0
2ejy	5	0.75	0.75	0.58	1.0	1.0	1.0
2e70	5	0.75	0.75	0.58	1.0	1.0	1.0
2dtt	5	0.75	0.75	0.58	1.0	1.0	1.0
2r2z	5	0.75	0.75	0.58	1.0	1.0	1.0
2dig	5	0.75	0.75	0.58	1.0	1.0	1.0
2d0p	5	0.75	0.75	0.58	1.0	1.0	1.0
2czv	5	0.75	0.75	0.58	1.0	1.0	1.0
2cw9	5	0.75	0.75	0.58	1.0	0.75	0.8
2c60	5	0.75	0.75	0.58	1.0	1.0	1.0
2bwf	5	0.75	0.75	0.58	1.0	0.75	0.8
2bud	5	0.75	0.75	0.58	1.0	1.0	1.0
2be3	5	0.75	0.75	0.61	1.0	0.75	0.8
2b8a	5	0.75	0.75	0.58	1.0	1.0	1.0
2b7l	5	0.75	0.75	0.58	1.0	0.75	0.8
2b4a	5	0.75	0.75	0.61	1.0	1.0	1.0
2a7y	5	0.75	0.75	0.58	1.0	1.0	1.0
1yop	5	0.75	1.0	0.8	1.0	1.0	1.0
1y96	5	0.75	0.75	0.58	1.0	1.0	1.0
1y71	5	0.75	0.75	0.58	1.0	1.0	1.0
1xyi	5	0.75	1.0	0.8	1.0	1.0	1.0
1ouz	5	0.75	1.0	0.8	1.0	1.0	1.0

1x69	5	0.75	0.75	0.61	1.0	1.0	1.0
1wmi	5	0.75	0.75	0.61	1.0	1.0	1.0
1wm3	5	0.75	0.75	0.58	1.0	1.0	1.0
1wjr	5	0.75	1.0	0.8	1.0	1.0	1.0
1vcc	5	0.75	0.75	0.61	1.0	1.0	1.0
1v9y	5	0.75	0.75	0.61	1.0	0.75	0.8
1v95	5	0.75	0.75	0.58	1.0	1.0	1.0
1uoy	5	0.75	1.0	0.8	1.0	1.0	1.0
1tpn	5	0.75	1.0	0.8	1.0	1.0	1.0
1th7	5	0.75	0.75	0.61	1.0	1.0	1.0
1t6s	5	0.75	1.0	0.8	0.75	1.0	0.8
1t62	5	0.75	0.75	0.58	1.0	1.0	1.0
1szv	5	0.75	0.75	0.58	1.0	0.75	0.8
1sjw	5	0.75	0.75	0.58	1.0	1.0	1.0
1s5u	5	0.75	0.75	0.58	0.75	0.75	0.58
1qmc	5	0.75	0.75	0.58	1.0	1.0	1.0
1ouz	5	0.75	1.0	0.8	1.0	1.0	1.0
1o50	5	0.75	1.0	0.8	1.0	1.0	1.0
1o26	5	0.75	0.75	0.61	1.0	1.0	1.0
1nz9	5	0.75	0.75	0.58	1.0	1.0	1.0
1nrv	5	0.75	0.75	0.58	0.75	0.75	0.61
1mwj	5	0.75	0.75	0.58	1.0	1.0	1.0
1muz	5	0.75	0.75	0.58	1.0	1.0	1.0
1m4j	5	0.75	0.75	0.61	1.0	1.0	1.0
1m2f	5	0.75	0.75	0.61	0.75	0.75	0.58
1m2d	5	0.75	0.75	0.61	0.75	0.75	0.61

1jwa	5	0.75	0.75	0.61	0.75	0.75	0.58
1jl1	5	0.75	0.75	0.58	1.0	0.75	0.8
1jeo	5	0.75	0.75	0.61	0.75	0.75	0.61
2gyb	5	0.75	0.75	0.61	1.0	0.75	0.8
1jbe	5	0.75	0.75	0.61	0.75	1.0	0.8
1iou	5	0.75	0.75	0.58	1.0	0.75	0.8
1hxv	5	0.75	0.75	0.58	1.0	1.0	1.0
1hkq	5	0.75	1.0	0.8	0.75	1.0	0.8
1h4x	5	0.75	0.75	0.61	0.75	0.75	0.58
1h3z	5	0.75	0.75	0.58	0.75	0.75	0.58
1h3q	5	0.75	0.75	0.58	1.0	0.75	0.8
1gxu	5	0.75	0.75	0.58	0.75	0.75	0.58
1gh8	5	0.75	0.75	0.61	1.0	1.0	1.0
1g4f	5	0.75	1.0	0.8	1.0	1.0	1.0
1f51	5	0.75	0.75	0.61	0.75	0.75	0.61
1dcf	5	0.75	0.75	0.61	0.75	0.75	0.61
1cxq	5	0.75	0.75	0.61	1.0	0.75	0.8
1bj8	5	0.75	1.0	0.8	1.0	1.0	1.0
1b1a	5	0.75	0.75	0.58	1.0	1.0	1.0
1aps	5	0.75	0.75	0.58	1.0	1.0	1.0
2w0n	5	0.67	0.67	0.54	1.0	1.0	1.0
1vd4	5	0.67	0.67	0.52	1.0	1.0	1.0
1pco	5	0.67	0.67	0.52	1.0	1.0	1.0
1oey	5	0.67	0.5	0.39	1.0	1.0	1.0
1f7l	5	0.67	0.5	0.36	1.0	0.75	0.8
3dkm	5	0.5	0.5	0.17	1.0	1.0	1.0

3dbo	5	0.5	0.5	0.17	1.0	1.0	1.0
3cfy	5	0.5	0.5	0.25	0.5	0.5	0.17
3bps	5	0.5	0.5	0.17	0.75	0.75	0.58
2ze8	5	0.5	0.5	0.17	0.75	0.75	0.58
2yx6	5	0.5	0.5	0.21	0.67	0.5	0.39
2vil	5	0.5	0.5	0.25	0.75	0.75	0.61
2uwq	5	0.5	0.5	0.17	0.5	0.5	0.21
2qyb	5	0.5	0.5	0.17	0.5	0.5	0.17
2qr3	5	0.5	0.5	0.25	0.5	0.5	0.21
2ql8	5	0.5	0.67	0.39	0.67	0.67	0.54
2ql6	5	0.5	0.5	0.17	0.75	0.75	0.58
2pg3	5	0.5	0.5	0.21	0.75	0.75	0.58
2oq3	5	0.5	0.5	0.17	0.67	0.5	0.39
2ooy	5	0.5	0.5	0.21	1.0	1.0	1.0
2ogk	5	0.5	0.5	0.17	1.0	1.0	1.0
2ogh	5	0.5	0.5	0.17	1.0	0.75	0.8
2nlw	5	0.5	0.5	0.17	0.5	0.5	0.21
2jyw	5	0.5	0.5	0.25	0.5	0.5	0.21
2jvn	5	0.5	0.67	0.36	1.0	1.0	1.0
2ju5	5	0.5	0.5	0.17	0.75	0.75	0.61
2jmk	5	0.5	0.5	0.21	0.75	0.75	0.58
2jev	5	0.5	0.5	0.25	0.75	0.75	0.61
2j8b	5	0.5	0.67	0.36	0.75	1.0	0.8
2j8a	5	0.5	0.5	0.17	1.0	1.0	1.0
2j6b	5	0.5	0.5	0.17	0.5	0.5	0.21
2ipq	5	0.5	0.5	0.17	1.0	1.0	1.0

2inn	5	0.5	0.5	0.21	0.75	0.75	0.61
2iim	5	0.5	0.5	0.21	1.0	1.0	1.0
2i9s	5	0.5	0.5	0.17	0.75	0.75	0.58
2i4a	5	0.5	0.5	0.17	1.0	1.0	1.0
2hqo	5	0.5	0.5	0.25	0.75	0.75	0.58
2hlr	5	0.5	0.67	0.36	0.67	0.67	0.52
2hjq	5	0.5	0.67	0.36	0.5	0.67	0.36
2hgv	5	0.5	0.5	0.17	1.0	1.0	1.0
2hfn	5	0.5	0.5	0.17	1.0	1.0	1.0
2hfd	5	0.5	0.5	0.17	0.5	0.5	0.17
2heq	5	0.5	0.5	0.21	0.75	0.75	0.61
2hd7	5	0.5	0.5	0.25	0.75	0.75	0.61
2grj	5	0.5	0.5	0.17	0.75	0.75	0.58
2fps	5	0.5	0.5	0.21	0.75	0.75	0.58
2fb6	5	0.5	0.5	0.17	0.75	0.75	0.61
2ers	5	0.5	0.67	0.36	0.5	0.67	0.36
2ekh	5	0.5	0.5	0.21	1.0	1.0	1.0
2egi	5	0.5	0.5	0.17	1.0	1.0	1.0
2ege	5	0.5	0.5	0.21	1.0	1.0	1.0
2e79	5	0.5	0.5	0.17	0.75	0.75	0.58
1mkk	5	0.5	0.5	0.17	0.75	0.75	0.58
2e2z	5	0.5	0.67	0.36	0.75	0.75	0.58
2dum	5	0.5	0.5	0.21	0.75	0.75	0.58
2dgx	5	0.5	0.5	0.17	1.0	1.0	1.0
2dbc	5	0.5	0.5	0.25	0.5	0.5	0.17
2csq	5	0.5	0.5	0.21	1.0	1.0	1.0

2byf	5	0.5	0.5	0.17	0.5	0.5	0.21
2bye	5	0.5	0.67	0.36	0.5	0.67	0.36
2btt	5	0.5	0.5	0.21	1.0	1.0	1.0
2bsq	5	0.5	0.5	0.25	0.67	0.5	0.39
2ayy	5	0.5	0.5	0.25	0.5	0.5	0.25
2ayd	5	0.5	0.5	0.21	0.75	0.75	0.58
2al3	5	0.5	0.5	0.25	1.0	1.0	1.0
2a6q	5	0.5	0.5	0.17	1.0	1.0	1.0
2a6q	5	0.5	0.5	0.17	1.0	1.0	1.0
1zuu	5	0.5	0.67	0.36	1.0	1.0	1.0
1zma	5	0.5	0.5	0.17	0.5	0.5	0.21
1zlh	5	0.5	0.67	0.36	0.75	1.0	0.8
1z1d	5	0.5	0.5	0.21	0.5	0.5	0.21
1z1d	5	0.5	0.5	0.21	0.75	0.75	0.58
1x5p	5	0.5	0.5	0.17	0.75	0.75	0.58
1wxv	5	0.5	0.5	0.17	0.75	0.75	0.58
1wx7	5	0.5	0.5	0.25	0.75	0.75	0.58
1wwr	5	0.5	0.5	0.21	0.67	0.5	0.39
1wmh	5	0.5	0.5	0.21	0.75	0.75	0.61
1wmh	5	0.5	0.5	0.21	0.75	0.75	0.61
1wjj	5	0.5	0.4	0.069	0.75	0.6	0.45
1wif	5	0.5	0.67	0.39	0.75	0.75	0.58
1wg1	5	0.5	0.5	0.17	0.75	0.75	0.58
1we7	5	0.5	0.5	0.21	1.0	0.75	0.8
1vyc	5	0.5	0.67	0.36	0.5	0.67	0.36
1v9w	5	0.5	0.5	0.17	1.0	1.0	1.0

1v6p	5	0.5	0.67	0.36	1.0	1.0	1.0
1v1c	5	0.5	0.5	0.21	1.0	1.0	1.0
1uhc	5	0.5	0.5	0.21	1.0	1.0	1.0
1u5s	5	0.5	0.5	0.21	1.0	1.0	1.0
1u07	5	0.5	0.67	0.36	0.5	0.67	0.36
1ttn	5	0.5	0.5	0.25	0.75	0.75	0.58
1to0	5	0.5	0.5	0.17	0.5	0.5	0.17
1t3o	5	0.5	0.67	0.39	0.75	1.0	0.8
1t0g	5	0.5	0.5	0.17	0.5	0.5	0.17
1spk	5	0.5	0.5	0.21	1.0	1.0	1.0
1sn8	5	0.5	0.4	0	1.0	0.8	0.82
1s60	5	0.5	0.5	0.25	1.0	1.0	1.0
1ryl	5	0.5	0.5	0.25	1.0	1.0	1.0
1rdu	5	0.5	0.5	0.21	0.67	0.5	0.39
1qp3	5	0.5	0.5	0.21	1.0	1.0	1.0
1tev	5	0.5	0.5	0.21	0.67	0.5	0.39
1qmp	5	0.5	0.5	0.17	0.67	0.5	0.39
1qgv	5	0.5	0.5	0.21	0.5	0.5	0.17
1pz4	5	0.5	0.5	0.25	0.75	0.75	0.58
1p6o	5	0.5	0.5	0.21	0.67	0.5	0.39
1p5z	5	0.5	0.5	0.17	0.5	0.5	0.17
1ovq	5	0.5	0.5	0.21	0.5	0.5	0.21
1oft	5	0.5	0.5	0.21	0.75	0.75	0.61
1o6b	5	0.5	0.5	0.21	0.75	0.75	0.61
1n62	5	0.5	0.5	0.17	0.75	0.75	0.58
1n62	5	0.5	0.5	0.17	1.0	1.0	1.0

1n5l	5	0.5	0.5	0.17	0.5	0.5	0.17
1kuf	5	0.5	0.5	0.17	0.75	0.75	0.58
1ktu	5	0.5	0.5	0.17	0.75	0.75	0.58
1kq6	5	0.5	0.5	0.17	1.0	1.0	1.0
1kg1	5	0.5	0.67	0.36	0.67	0.67	0.52
1jx7	5	0.5	0.5	0.21	0.67	0.5	0.36
1jt8	5	0.5	0.4	0	1.0	1.0	1.0
1imt	5	0.5	0.67	0.36	0.5	0.67	0.36
1igu	5	0.5	0.5	0.21	0.75	0.75	0.61
1i35	5	0.5	0.5	0.17	0.67	0.5	0.36
1h8c	5	0.5	0.5	0.21	0.75	0.75	0.61
1gh2	5	0.5	0.5	0.17	0.75	0.75	0.58
1fq1	5	0.5	0.5	0.17	0.75	0.75	0.58
1ewi	5	0.5	0.67	0.36	1.0	1.0	1.0
1es9	5	0.5	0.5	0.21	0.75	0.75	0.58
1es7	5	0.5	0.67	0.36	0.67	0.67	0.52
1e9m	5	0.5	0.5	0.17	0.5	0.5	0.21
1dtd	5	0.5	0.5	0.17	1.0	1.0	1.0
1def	5	0.5	0.67	0.36	1.0	1.0	1.0
1d4b	5	0.5	0.67	0.36	0.5	0.67	0.39
1cuj	5	0.5	0.5	0.21	0.75	0.75	0.58
1c7k	5	0.5	0.5	0.17	1.0	1.0	1.0
1c05	5	0.5	0.5	0.21	1.0	1.0	1.0
1bym	5	0.5	0.5	0.21	1.0	1.0	1.0
1aiw	5	0.5	0.67	0.36	1.0	1.0	1.0
1bhu	5	0.33	0.33	0.048	0.67	0.67	0.52

3cg6	5	0.25	0.25	-0.25	0.5	0.5	0.17
2yra	5	0.25	0.33	-0.089	0.5	0.67	0.36
2jx9	5	0.25	0.33	-0.089	0.5	0.67	0.36
2hw0	5	0.25	0.25	-0.25	1.0	1.0	1.0
2fc7	5	0.25	0.33	-0.089	1.0	1.0	1.0
2dmz	5	0.25	0.33	-0.039	0.75	1.0	0.8
2ccg	5	0.25	0.25	-0.18	0.5	0.5	0.17
2bop	5	0.25	0.25	-0.18	0.5	0.5	0.17
2b3n	5	0.25	0.25	-0.25	0.5	0.5	0.17
2a8c	5	0.25	0.25	-0.18	0.67	0.5	0.39
1yzs	5	0.25	0.25	-0.25	0.5	0.5	0.21
1wrm	5	0.25	0.25	-0.18	0.5	0.5	0.21
1wn2	5	0.25	0.25	-0.18	0.5	0.5	0.21
1tz0	5	0.25	0.25	-0.25	0.5	0.5	0.17
1tq1	5	0.25	0.25	-0.18	0.5	0.5	0.21
1ssk	5	0.25	0.25	-0.25	0.5	0.5	0.17
1rh6	5	0.25	0.33	-0.089	0.5	0.67	0.36
1q7l	5	0.25	0.25	-0.18	0.67	0.67	0.54
1pux	5	0.25	0.33	-0.089	0.67	0.67	0.54
1prt	5	0.25	0.2	-0.41	0.67	0.4	0.26
1n13	5	0.25	0.33	-0.089	0.5	0.67	0.36
1l1s	5	0.25	0.25	-0.18	0.33	0.25	-0.039
1fzt	5	0.25	0.25	-0.25	0.5	0.5	0.17
1f08	5	0.25	0.25	-0.25	0.5	0.5	0.17
1a0r	5	0.25	0.25	-0.18	0.5	0.5	0.25
3edo	6	1.0	1.0	1.0	1.0	1.0	1.0

2row	6	1.0	1.0	1.0	1.0	1.0	1.0
2rng	6	1.0	1.0	1.0	1.0	1.0	1.0
2qzf	6	1.0	1.0	1.0	1.0	1.0	1.0
2qgx	6	1.0	1.0	1.0	1.0	1.0	1.0
3ddv	6	1.0	0.8	0.85	1.0	0.8	0.85
2p19	6	1.0	0.8	0.85	1.0	0.8	0.85
2ogg	6	1.0	0.8	0.85	1.0	0.8	0.85
2ijr	6	1.0	0.8	0.85	1.0	0.8	0.85
2ia9	6	1.0	1.0	1.0	1.0	1.0	1.0
2ht6	6	1.0	0.8	0.85	1.0	0.8	0.85
2hh8	6	1.0	0.8	0.85	1.0	0.8	0.85
2ewc	6	1.0	0.8	0.85	1.0	0.8	0.85
2bho	6	1.0	1.0	1.0	1.0	1.0	1.0
1ywu	6	1.0	0.83	0.87	1.0	0.67	0.74
1xn5	6	1.0	1.0	1.0	1.0	1.0	1.0
1v61	6	1.0	1.0	1.0	1.0	1.0	1.0
1tt8	6	1.0	0.8	0.85	1.0	1.0	1.0
1qw2	6	1.0	1.0	1.0	1.0	1.0	1.0
1of5	6	1.0	0.8	0.85	1.0	0.8	0.85
1of5	6	1.0	0.8	0.85	1.0	0.8	0.85
1ly7	6	1.0	1.0	1.0	1.0	0.8	0.85
1kaf	6	1.0	1.0	1.0	1.0	1.0	1.0
1hl6	6	1.0	0.8	0.85	1.0	0.8	0.85
1h5p	6	1.0	0.8	0.85	1.0	0.6	0.71
1ew4	6	1.0	0.8	0.85	1.0	0.8	0.85
1d4o	6	1.0	0.8	0.85	1.0	0.8	0.85

1bxn	6	1.0	1.0	1.0	1.0	1.0	1.0
1buj	6	1.0	1.0	1.0	1.0	1.0	1.0
3dxo	6	0.8	0.8	0.7	1.0	1.0	1.0
3chb	6	0.8	0.8	0.7	1.0	0.8	0.85
2zpl	6	0.8	1.0	0.85	1.0	1.0	1.0
2z13	6	0.8	0.8	0.7	0.8	0.8	0.7
2vc8	6	0.8	0.8	0.7	0.8	0.8	0.7
2qs9	6	0.8	0.8	0.71	0.8	0.8	0.7
2ppn	6	0.8	0.8	0.7	0.8	0.8	0.7
2p04	6	0.8	0.8	0.7	1.0	0.8	0.85
2ozx	6	0.8	0.8	0.7	0.8	0.8	0.7
2nwi	6	0.8	0.8	0.7	1.0	0.8	0.85
2nup	6	0.8	0.8	0.7	0.8	0.8	0.7
2k31	6	0.8	0.8	0.71	1.0	1.0	1.0
2jso	6	0.8	0.8	0.7	1.0	1.0	1.0
2i6v	6	0.8	0.8	0.7	1.0	0.8	0.85
1q6w	6	0.8	0.8	0.7	1.0	0.8	0.85
2h28	6	0.8	0.8	0.7	1.0	1.0	1.0
2eey	6	0.8	0.8	0.7	0.8	0.8	0.7
2dog	6	0.8	0.67	0.58	0.8	0.67	0.58
2czq	6	0.8	0.8	0.71	0.8	0.8	0.7
2bny	6	0.8	0.8	0.7	0.8	0.8	0.7
2bmo	6	0.8	0.8	0.7	0.8	0.8	0.7
2axi	6	0.8	1.0	0.85	0.8	1.0	0.85
1yua	6	0.8	1.0	0.85	0.8	1.0	0.85
1yr1	6	0.8	0.8	0.7	1.0	1.0	1.0

1xs0	6	0.8	0.8	0.71	1.0	1.0	1.0
1wxc	6	0.8	1.0	0.85	1.0	1.0	1.0
1vhm	6	0.8	0.8	0.71	1.0	0.8	0.85
1tii	6	0.8	0.8	0.7	0.8	0.8	0.7
1pfs	6	0.8	0.8	0.7	1.0	1.0	1.0
1nso	6	0.8	1.0	0.85	1.0	1.0	1.0
1nrj	6	0.8	0.8	0.71	1.0	0.8	0.85
1hkx	6	0.8	0.8	0.7	1.0	1.0	1.0
1ghj	6	0.8	0.8	0.7	0.8	0.8	0.7
1mf7	6	0.8	0.8	0.71	1.0	1.0	1.0
1ll8	6	0.8	0.8	0.7	1.0	1.0	1.0
1l3g	6	0.8	1.0	0.85	0.8	1.0	0.85
1is7	6	0.8	0.8	0.7	1.0	0.8	0.85
1fe8	6	0.8	0.8	0.71	1.0	1.0	1.0
3dkz	6	0.75	0.6	0.53	1.0	0.8	0.85
3b64	6	0.75	0.75	0.66	1.0	1.0	1.0
2qiy	6	0.75	0.6	0.53	1.0	0.8	0.85
2p11	6	0.6	0.75	0.53	1.0	0.8	0.85
2j5x	6	0.75	0.6	0.53	1.0	0.8	0.85
2imj	6	0.75	0.6	0.53	0.75	0.6	0.53
2grg	6	0.75	0.6	0.53	1.0	0.8	0.85
2ghf	6	0.75	0.75	0.66	1.0	1.0	1.0
1x5r	6	0.75	1.0	0.83	0.75	1.0	0.83
1wql	6	0.75	0.6	0.54	0.75	0.6	0.54
1vrq	6	0.75	0.75	0.66	1.0	1.0	1.0
1vh9	6	0.75	0.6	0.53	1.0	0.8	0.85

1tsf	6	0.75	0.6	0.53	1.0	0.8	0.85
1psu	6	0.75	0.6	0.53	1.0	0.8	0.85
1oqj	6	0.75	0.75	0.66	1.0	0.75	0.83
1mby	6	0.75	0.75	0.66	1.0	1.0	1.0
1m6k	6	0.75	0.6	0.53	1.0	1.0	1.0
1hfo	6	0.75	0.75	0.66	1.0	0.75	0.83
1fl9	6	0.75	0.6	0.53	1.0	0.8	0.85
1a7i	6	0.75	1.0	0.83	1.0	1.0	1.0
3er7	6	0.6	0.6	0.4	1.0	1.0	1.0
3ef8	6	0.6	0.6	0.4	0.6	0.6	0.4
3e4g	6	0.6	0.6	0.42	0.75	0.6	0.54
3e29	6	0.6	0.6	0.4	1.0	1.0	1.0
2re2	6	0.6	0.6	0.42	1.0	1.0	1.0
2q3g	6	0.6	0.75	0.54	1.0	1.0	1.0
2pqj	6	0.6	0.6	0.43	0.75	0.6	0.53
2pfi	6	0.6	0.75	0.54	0.6	0.75	0.53
2os6	6	0.6	0.75	0.54	0.8	1.0	0.85
2okh	6	0.6	0.6	0.4	0.75	0.6	0.53
2ocs	6	0.6	0.75	0.53	0.8	1.0	0.85
2k2o	6	0.6	0.6	0.4	0.8	0.8	0.7
2k0m	6	0.6	0.75	0.53	0.8	1.0	0.85
2iec	6	0.6	0.75	0.53	1.0	1.0	1.0
2i9y	6	0.6	0.6	0.42	1.0	1.0	1.0
2h41	6	0.6	0.75	0.53	0.6	0.75	0.53
2h3l	6	0.6	0.6	0.42	0.8	0.8	0.7
2gzv	6	0.6	0.6	0.42	0.8	0.8	0.7

2g1u	6	0.6	0.6	0.4	0.6	0.6	0.4
2emq	6	0.6	0.75	0.53	0.8	1.0	0.85
2ehg	6	0.6	0.6	0.4	1.0	1.0	1.0
1t3y	6	0.6	0.6	0.4	1.0	1.0	1.0
2ehb	6	0.6	0.6	0.4	1.0	1.0	1.0
2e12	6	0.6	0.6	0.42	0.8	0.8	0.7
2dtc	6	0.6	0.6	0.42	1.0	1.0	1.0
2daz	6	0.6	0.75	0.53	1.0	1.0	1.0
2bw2	6	0.6	0.75	0.53	0.6	0.75	0.53
2bjo	6	0.6	0.75	0.54	0.6	0.75	0.53
2ahm	6	0.6	0.6	0.43	1.0	1.0	1.0
2ab5	6	0.6	0.75	0.53	0.6	0.75	0.53
1zbf	6	0.6	0.6	0.42	0.75	0.6	0.53
1xqs	6	0.6	0.75	0.53	0.6	0.75	0.53
1xo1	6	0.6	0.6	0.4	0.75	0.6	0.53
1xd3	6	0.6	0.6	0.4	0.6	0.6	0.4
1x45	6	0.6	0.75	0.53	0.75	0.75	0.66
1vr7	6	0.6	0.6	0.4	1.0	0.8	0.85
1ujv	6	0.6	0.75	0.54	0.8	1.0	0.85
1sqr	6	0.6	0.6	0.4	0.8	0.8	0.7
1sgo	6	0.6	0.6	0.4	0.8	0.8	0.7
1s3c	6	0.6	0.75	0.53	0.75	0.75	0.66
1ry9	6	0.6	0.6	0.4	0.6	0.6	0.4
1rgx	6	0.6	0.75	0.53	0.75	0.75	0.66
1r6j	6	0.6	0.75	0.54	0.8	1.0	0.85
1qu9	6	0.6	0.6	0.4	0.8	0.8	0.71

1qst	6	0.6	0.6	0.43	0.8	0.8	0.7
1o7c	6	0.6	0.6	0.4	0.6	0.6	0.4
1o08	6	0.6	0.6	0.43	0.6	0.6	0.43
1m5z	6	0.6	0.75	0.54	0.8	1.0	0.85
1l0o	6	0.6	0.6	0.4	0.8	0.8	0.7
1kpt	6	0.6	0.75	0.53	1.0	1.0	1.0
1kld	6	0.6	1.0	0.71	1.0	1.0	1.0
1k3e	6	0.6	0.75	0.53	0.8	1.0	0.85
1i18	6	0.6	0.5	0.29	1.0	0.83	0.87
1g66	6	0.6	0.6	0.43	0.6	0.6	0.4
1fl7	6	0.6	1.0	0.71	0.6	1.0	0.71
1dkg	6	0.6	0.75	0.53	0.6	0.75	0.53
1aje	6	0.6	0.6	0.4	1.0	0.8	0.85
3d9r	6	0.5	0.4	0.21	0.75	0.75	0.66
2ywk	6	0.5	0.5	0.32	0.75	0.75	0.66
2prx	6	0.5	0.4	0.21	0.8	0.8	0.7
2peh	6	0.5	0.5	0.32	0.75	0.75	0.67
2o16	6	0.5	0.5	0.32	0.75	0.75	0.67
2j9c	6	0.5	0.5	0.32	1.0	1.0	1.0
2iwr	6	0.5	0.4	0.23	0.75	0.6	0.54
2h8e	6	0.5	0.5	0.32	0.75	0.75	0.67
2ec2	6	0.5	0.5	0.32	1.0	1.0	1.0
2dmc	6	0.5	0.5	0.32	0.5	0.5	0.32
2div	6	0.5	0.5	0.32	1.0	1.0	1.0
2dit	6	0.5	0.5	0.32	1.0	1.0	1.0
2aua	6	0.5	0.5	0.32	1.0	1.0	1.0

2arf	6	0.5	0.4	0.23	0.75	0.6	0.54
1wwh	6	0.5	0.5	0.32	1.0	1.0	1.0
1wey	6	0.5	0.5	0.32	0.75	0.75	0.66
1v30	6	0.5	0.5	0.32	0.75	0.75	0.66
1tp6	6	0.5	0.4	0.21	0.6	0.6	0.4
1q0p	6	0.5	0.4	0.25	0.75	0.75	0.66
2k87	6	0.5	0.5	0.32	0.5	0.5	0.32
1pdg	6	0.5	0.67	0.45	0.75	1.0	0.83
1gy7	6	0.5	0.4	0.21	1.0	0.8	0.85
1f53	6	0.5	0.5	0.32	0.75	0.75	0.66
4ull	6	0.4	0.5	0.21	0.6	0.75	0.53
3vtk	6	0.4	0.4	0.1	0.6	0.6	0.4
3eze	6	0.4	0.4	0.1	0.6	0.75	0.54
3edh	6	0.4	0.5	0.23	0.6	0.75	0.54
3da5	6	0.4	0.4	0.13	0.8	0.8	0.7
3ctu	6	0.4	0.5	0.21	0.8	1.0	0.85
3by7	6	0.4	0.4	0.1	0.6	0.6	0.4
3b8f	6	0.4	0.4	0.13	0.6	0.6	0.42
2zef	6	0.4	0.4	0.13	0.5	0.4	0.23
2z8o	6	0.4	0.4	0.1	0.6	0.6	0.4
2v9v	6	0.4	0.5	0.21	0.6	0.75	0.53
2uv4	6	0.4	0.5	0.23	0.5	0.5	0.32
2rnk	6	0.4	0.5	0.21	0.6	0.75	0.53
2qwz	6	0.4	0.4	0.1	0.8	0.8	0.7
2pqx	6	0.4	0.5	0.21	0.8	0.8	0.7
2p4v	6	0.4	0.5	0.21	0.4	0.5	0.21

2oya	6	0.4	0.5	0.21	0.4	0.5	0.21
2o03	6	0.4	0.5	0.21	0.4	0.5	0.21
2k5q	6	0.4	0.5	0.21	0.6	0.75	0.53
2jzr	6	0.4	0.4	0.13	0.4	0.4	0.1
2hxm	6	0.4	0.5	0.21	1.0	1.0	1.0
2hpa	6	0.4	0.4	0.13	0.4	0.4	0.13
2hp7	6	0.4	0.4	0.1	1.0	0.8	0.85
2e5o	6	0.4	0.4	0.13	0.6	0.6	0.42
2dyt	6	0.4	0.4	0.15	0.6	0.6	0.43
2bfw	6	0.4	0.4	0.15	0.4	0.4	0.13
2b5x	6	0.4	0.4	0.13	0.6	0.6	0.4
2b3d	6	0.4	0.4	0.13	0.5	0.4	0.21
1z6i	6	0.4	0.4	0.1	0.6	0.6	0.4
1z5f	6	0.4	0.5	0.21	0.5	0.5	0.32
1yzv	6	0.4	0.4	0.13	0.6	0.6	0.43
1yb0	6	0.4	0.4	0.13	0.4	0.4	0.1
1y5h	6	0.4	0.5	0.25	0.5	0.5	0.32
1win	6	0.4	0.5	0.21	0.4	0.5	0.21
1ujx	6	0.4	0.5	0.23	0.6	0.75	0.53
1t82	6	0.4	0.4	0.1	0.8	0.8	0.7
1s7i	6	0.4	0.5	0.21	0.8	1.0	0.85
1pqw	6	0.4	0.4	0.13	0.4	0.4	0.15
1ok0	6	0.4	0.5	0.21	0.4	0.5	0.21
1nww	6	0.4	0.4	0.1	0.4	0.4	0.13
1nus	6	0.4	0.4	0.17	0.4	0.4	0.13
1nf9	6	0.4	0.4	0.15	0.4	0.4	0.13

1mxi	6	0.4	0.4	0.13	0.5	0.4	0.21
1ml8	6	0.4	0.5	0.23	0.5	0.5	0.33
1lkn	6	0.4	0.4	0.1	0.8	0.8	0.7
1k8h	6	0.4	0.5	0.21	0.6	0.75	0.53
1j22	6	0.4	0.4	0.13	0.6	0.6	0.4
1g10	6	0.4	0.5	0.21	0.4	0.5	0.21
1cjm	6	0.4	0.5	0.23	0.5	0.5	0.33
3eli	6	0.25	0.25	-0.023	0.5	0.5	0.32
2ctt	6	0.25	0.33	0.075	0.5	0.67	0.45
1wgp	6	0.25	0.25	-0.023	0.75	0.75	0.66
1pbu	6	0.25	0.2	-0.078	0.5	0.4	0.21
1o51	6	0.25	0.25	0	0.75	0.75	0.67
1mhd	6	0.25	0.33	0.075	0.5	0.67	0.45
1f94	6	0.25	0.25	-0.023	0.5	0.5	0.32
2jvb	6	0.2	0.25	-0.11	0.4	0.5	0.21
2ii7	6	0.2	0.2	-0.13	0.6	0.6	0.4
3d00	7	1.0	1.0	1.0	1.0	1.0	1.0
2vgl	7	1.0	1.0	1.0	1.0	0.8	0.87
2v76	7	1.0	0.83	0.88	1.0	0.83	0.88
2rov	7	1.0	1.0	1.0	1.0	1.0	1.0
2rnr	7	1.0	0.83	0.88	1.0	0.83	0.88
2r9a	7	1.0	1.0	1.0	1.0	1.0	1.0
2j97	7	1.0	0.83	0.88	1.0	0.83	0.88
2ivw	7	1.0	1.0	1.0	1.0	1.0	1.0
2hti	7	1.0	0.83	0.88	1.0	1.0	1.0
2htd	7	1.0	0.83	0.88	1.0	0.83	0.88

2ffs	7	1.0	0.83	0.88	1.0	0.83	0.88
2cy9	7	1.0	1.0	1.0	1.0	1.0	1.0
2cof	7	1.0	0.83	0.88	1.0	0.83	0.88
2bog	7	1.0	0.83	0.88	1.0	0.83	0.88
2b79	7	1.0	0.83	0.88	1.0	0.83	0.88
2asf	7	1.0	1.0	1.0	1.0	1.0	1.0
1zyi	7	1.0	0.83	0.88	1.0	0.83	0.88
1y5o	7	1.0	1.0	1.0	1.0	1.0	1.0
1vmh	7	1.0	1.0	1.0	1.0	1.0	1.0
1rfe	7	1.0	0.83	0.88	1.0	0.83	0.88
1pls	7	1.0	0.83	0.88	1.0	0.83	0.88
1ox9	7	1.0	1.0	1.0	1.0	1.0	1.0
1jfm	7	1.0	0.83	0.88	1.0	0.83	0.88
1i0v	7	1.0	1.0	1.0	1.0	1.0	1.0
1g6e	7	1.0	0.83	0.88	1.0	0.83	0.88
1euv	7	1.0	1.0	1.0	1.0	1.0	1.0
1ae9	7	1.0	1.0	1.0	1.0	1.0	1.0
2k0q	7	0.83	1.0	0.88	1.0	1.0	1.0
2d9r	7	0.83	0.83	0.77	1.0	1.0	1.0
2a15	7	0.83	0.83	0.77	0.83	0.83	0.77
1twc	7	0.83	1.0	0.88	1.0	1.0	1.0
3dfz	7	0.8	0.8	0.74	0.8	0.8	0.74
3db0	7	0.8	0.67	0.64	1.0	1.0	1.0
2z33	7	0.8	0.8	0.74	1.0	1.0	1.0
2vgr	7	0.8	0.67	0.64	1.0	0.83	0.88
2v7s	7	0.8	0.8	0.74	1.0	1.0	1.0

2qkm	7	0.8	0.67	0.64	1.0	1.0	1.0
2q7b	7	0.8	0.67	0.64	0.8	0.67	0.64
2pth	7	0.8	0.67	0.64	0.8	0.67	0.64
2prf	7	0.8	0.8	0.74	1.0	0.8	0.87
2plw	7	0.8	0.67	0.64	1.0	0.83	0.88
2pkp	7	0.8	0.67	0.64	1.0	0.83	0.88
2p14	7	0.8	0.8	0.74	0.8	0.8	0.74
2oqb	7	0.8	0.67	0.64	1.0	0.83	0.88
2o99	7	0.8	0.8	0.74	1.0	0.83	0.88
2o30	7	0.8	0.8	0.74	0.8	0.8	0.74
2k49	7	0.8	0.8	0.74	0.8	0.8	0.74
2jpe	7	0.8	1.0	0.87	1.0	1.0	1.0
2jop	7	0.8	1.0	0.87	0.8	1.0	0.87
2jn9	7	0.8	0.8	0.74	0.8	0.8	0.74
2j8m	7	0.8	0.67	0.64	0.8	0.67	0.64
2i5f	7	0.8	0.67	0.64	0.8	0.67	0.64
2hpu	7	0.8	0.8	0.74	0.8	0.8	0.74
2g35	7	0.8	0.8	0.74	1.0	1.0	1.0
2g0b	7	0.8	0.67	0.64	1.0	1.0	1.0
2fhi	7	0.8	0.8	0.74	1.0	1.0	1.0
2ela	7	0.8	0.67	0.64	1.0	1.0	1.0
2ec1	7	0.8	0.67	0.64	0.8	0.67	0.64
2e6u	7	0.8	0.8	0.74	1.0	0.8	0.87
2dkq	7	0.8	0.67	0.64	1.0	0.8	0.87
2dg2	7	0.8	0.67	0.64	0.8	0.67	0.64
2da0	7	0.8	0.67	0.64	0.8	0.67	0.64

2bse	7	0.8	0.67	0.64	0.8	0.67	0.64
2apn	7	0.8	0.8	0.74	1.0	1.0	1.0
2akk	7	0.8	0.8	0.74	1.0	1.0	1.0
2aiv	7	0.8	0.8	0.74	1.0	1.0	1.0
2adz	7	0.8	0.8	0.74	1.0	1.0	1.0
1zr5	7	0.8	0.67	0.64	1.0	1.0	1.0
1yub	7	0.8	0.67	0.64	1.0	0.83	0.88
1xke	7	0.8	0.67	0.64	1.0	1.0	1.0
1wgv	7	0.8	0.8	0.74	1.0	1.0	1.0
1wgu	7	0.8	0.67	0.64	1.0	0.83	0.88
1wft	7	0.8	0.8	0.74	1.0	0.83	0.88
1upq	7	0.8	0.67	0.64	1.0	0.83	0.88
1unq	7	0.8	0.67	0.64	0.8	0.67	0.64
1u6l	7	0.8	0.8	0.74	1.0	0.83	0.88
1tqz	7	0.8	0.67	0.64	1.0	0.83	0.88
1t23	7	0.8	1.0	0.87	1.0	1.0	1.0
1t17	7	0.8	0.67	0.64	1.0	0.83	0.88
1qle	7	0.8	1.0	0.87	0.8	1.0	0.87
1q67	7	0.8	0.67	0.64	1.0	0.83	0.88
1m7e	7	0.8	0.67	0.64	1.0	0.83	0.88
1ivz	7	0.8	0.8	0.74	1.0	1.0	1.0
1idp	7	0.8	0.67	0.64	1.0	0.83	0.88
1gme	7	0.8	0.8	0.74	1.0	1.0	1.0
1fwq	7	0.8	0.67	0.64	1.0	0.83	0.88
1bsh	7	0.8	0.8	0.74	0.8	0.8	0.74
3ci6	7	0.67	0.67	0.54	0.83	0.83	0.77

3bnw	7	0.67	0.67	0.53	1.0	0.83	0.88
2v1l	7	0.67	0.67	0.53	1.0	0.83	0.88
2jne	7	0.67	0.8	0.64	1.0	1.0	1.0
2ge9	7	0.67	0.8	0.64	0.8	0.8	0.74
2car	7	0.67	0.67	0.54	0.83	0.83	0.77
2c6u	7	0.67	0.67	0.53	0.83	0.83	0.77
1z1s	7	0.67	0.67	0.54	0.8	0.67	0.64
1xzq	7	0.67	0.67	0.53	1.0	0.83	0.88
1wgq	7	0.67	0.67	0.53	1.0	0.83	0.88
1vk0	7	0.67	0.67	0.53	0.8	0.67	0.64
1k85	7	0.67	0.8	0.64	0.8	0.8	0.74
1ipi	7	0.67	0.8	0.64	0.8	0.8	0.74
1exc	7	0.67	0.67	0.54	0.8	0.67	0.64
3vub	7	0.6	0.5	0.39	0.6	0.5	0.39
3e0x	7	0.6	0.5	0.39	0.6	0.5	0.39
3dsb	7	0.6	0.6	0.47	0.8	0.67	0.64
3dh0	7	0.6	0.5	0.39	0.8	0.67	0.64
3d3s	7	0.6	0.5	0.39	0.8	0.67	0.64
3cgy	7	0.6	0.6	0.47	0.8	0.8	0.74
3bq8	7	0.6	0.6	0.48	0.8	0.8	0.74
3bf2	7	0.6	0.6	0.47	0.8	0.8	0.74
2ys3	7	0.6	0.6	0.47	0.75	0.6	0.58
2vq8	7	0.6	0.6	0.47	0.8	0.8	0.74
2ron	7	0.6	0.5	0.39	0.8	0.67	0.64
2qjl	7	0.6	0.6	0.49	0.75	0.6	0.59
2qim	7	0.6	0.5	0.39	0.8	0.67	0.64

2pc1	7	0.6	0.5	0.4	0.8	0.67	0.64
2p7h	7	0.6	0.5	0.4	0.8	0.67	0.64
2ore	7	0.6	0.5	0.39	0.8	0.67	0.64
2jdc	7	0.6	0.5	0.4	0.8	0.67	0.64
2i8g	7	0.6	0.5	0.39	1.0	1.0	1.0
2i02	7	0.6	0.6	0.48	1.0	1.0	1.0
2h1d	7	0.6	0.5	0.4	0.6	0.5	0.39
2h00	7	0.6	0.5	0.4	0.8	0.67	0.64
2g7j	7	0.6	0.6	0.47	0.8	0.8	0.74
2flh	7	0.6	0.5	0.39	0.8	0.67	0.64
2fjl	7	0.6	0.6	0.47	0.6	0.6	0.47
2ee3	7	0.6	0.6	0.47	0.75	0.6	0.58
2di7	7	0.6	0.75	0.58	0.8	1.0	0.87
2d2a	7	0.6	0.6	0.47	0.8	0.8	0.74
2ccv	7	0.6	0.6	0.47	0.8	0.8	0.74
2ae6	7	0.6	0.5	0.4	0.6	0.5	0.4
1zvp	7	0.6	0.6	0.47	0.8	0.8	0.74
1ys5	7	0.6	0.5	0.39	0.67	0.67	0.53
1wqu	7	0.6	0.6	0.48	0.75	0.6	0.58
1whm	7	0.6	0.6	0.48	0.8	0.8	0.74
1wek	7	0.6	0.5	0.4	0.8	0.8	0.74
1usv	7	0.6	0.6	0.47	0.67	0.8	0.64
1ub4	7	0.6	0.5	0.39	0.8	0.8	0.74
1sg5	7	0.6	0.6	0.47	0.8	0.8	0.74
1o5f	7	0.6	0.6	0.47	0.6	0.6	0.47
1nub	7	0.6	0.75	0.58	0.8	1.0	0.87

1l1e	7	0.6	0.5	0.39	0.8	0.67	0.64
1k7c	7	0.6	0.6	0.48	0.6	0.6	0.48
1jnz	7	0.6	0.75	0.58	0.6	0.75	0.58
1g84	7	0.6	0.6	0.47	0.8	0.8	0.74
1equ	7	0.6	0.5	0.41	0.6	0.5	0.4
1egx	7	0.6	0.5	0.39	1.0	1.0	1.0
1e6v	7	0.6	0.6	0.47	1.0	1.0	1.0
1dy5	7	0.6	0.6	0.47	0.6	0.6	0.47
1d1n	7	0.6	0.6	0.47	0.8	0.8	0.74
1c01	7	0.6	0.6	0.47	0.8	0.8	0.74
1bxd	7	0.6	0.6	0.47	0.8	0.8	0.74
1aqz	7	0.6	0.6	0.48	0.8	0.8	0.74
2v3m	7	0.5	0.5	0.3	0.67	0.67	0.53
2ple	7	0.5	0.4	0.3	0.67	0.67	0.53
2ol5	7	0.5	0.5	0.3	0.83	0.83	0.77
2if4	7	0.5	0.5	0.3	0.67	0.67	0.53
2gax	7	0.5	0.5	0.38	0.6	0.75	0.59
1zk4	7	0.5	0.5	0.32	0.67	0.67	0.54
1yx0	7	0.5	0.6	0.41	0.6	0.6	0.49
1yhn	7	0.5	0.5	0.32	0.67	0.67	0.54
1yel	7	0.5	0.5	0.3	0.67	0.67	0.53
1ybj	7	0.5	0.5	0.3	0.67	0.67	0.53
1ut4	7	0.5	0.5	0.3	0.67	0.67	0.53
1u5d	7	0.5	0.5	0.3	0.8	0.67	0.64
1o8x	7	0.5	0.5	0.3	0.6	0.5	0.39
1flm	7	0.5	0.5	0.3	0.67	0.67	0.54

3ejg	7	0.4	0.33	0.14	0.6	0.5	0.39
2qvt	7	0.4	0.4	0.21	0.6	0.5	0.39
2pu3	7	0.4	0.5	0.31	0.4	0.5	0.31
2p28	7	0.4	0.5	0.3	0.5	0.5	0.38
2ouc	7	0.4	0.4	0.21	0.4	0.4	0.22
2nwu	7	0.4	0.5	0.31	0.4	0.5	0.31
2jgb	7	0.4	0.33	0.14	0.6	0.5	0.39
2cb9	7	0.4	0.33	0.15	0.6	0.5	0.39
2c71	7	0.4	0.4	0.23	0.6	0.6	0.48
1y93	7	0.4	0.4	0.23	0.4	0.4	0.23
1xiw	7	0.4	0.4	0.21	0.4	0.4	0.21
1xer	7	0.4	0.4	0.21	0.6	0.6	0.47
1wkt	7	0.4	0.4	0.21	0.6	0.6	0.47
1uc6	7	0.4	0.4	0.21	0.6	0.6	0.47
1rki	7	0.4	0.4	0.21	0.6	0.6	0.47
1ot4	7	0.4	0.4	0.21	0.4	0.4	0.21
1oa9	7	0.4	0.29	0.095	0.6	0.43	0.32
1mkb	7	0.4	0.33	0.14	0.4	0.33	0.14
1lo7	7	0.4	0.33	0.15	0.8	0.67	0.64
1jq7	7	0.4	0.33	0.14	0.6	0.5	0.39
1i1j	7	0.4	0.33	0.15	0.6	0.5	0.4
1gn1	7	0.4	0.4	0.21	0.6	0.6	0.47
1gmx	7	0.4	0.4	0.21	0.5	0.4	0.3
1egg	7	0.4	0.33	0.15	0.6	0.5	0.4
1ckv	7	0.4	0.4	0.21	0.6	0.6	0.47
3ceu	7	0.33	0.4	0.15	0.5	0.6	0.41

2ij9	7	0.33	0.33	0.098	0.33	0.33	0.083
2fvv	7	0.33	0.4	0.14	0.5	0.6	0.39
2c8t	7	0.33	0.33	0.098	0.33	0.33	0.083
1zce	7	0.33	0.33	0.083	0.33	0.33	0.083
1uex	7	0.33	0.4	0.14	0.5	0.6	0.39
1rku	7	0.33	0.33	0.098	0.33	0.33	0.12
3dl3	7	0.2	0.2	-0.05	0.4	0.4	0.21
2j8j	7	0.2	0.2	-0.05	0.4	0.4	0.21
2hgf	7	0.2	0.2	-0.05	0.4	0.4	0.21
3d4e	8	1.0	0.86	0.9	1.0	0.86	0.9
3c9q	8	1.0	1.0	1.0	1.0	1.0	1.0
3b4u	8	1.0	0.75	0.83	1.0	0.75	0.83
2r2y	8	1.0	0.86	0.9	1.0	0.86	0.9
2qcf	8	1.0	0.75	0.83	1.0	0.75	0.83
2okf	8	1.0	1.0	1.0	1.0	0.83	0.89
2nvn	8	1.0	1.0	1.0	1.0	1.0	1.0
2dch	8	1.0	1.0	1.0	1.0	1.0	1.0
1xxm	8	1.0	0.86	0.9	1.0	1.0	1.0
1wjm	8	1.0	0.86	0.9	1.0	0.86	0.9
1smp	8	1.0	0.75	0.83	1.0	0.75	0.83
1sfs	8	1.0	0.75	0.83	1.0	0.75	0.83
1npo	8	1.0	1.0	1.0	1.0	1.0	1.0
1hjq	8	1.0	0.75	0.83	1.0	0.75	0.83
3edp	8	0.83	0.83	0.79	1.0	1.0	1.0
3dcp	8	0.83	1.0	0.89	1.0	1.0	1.0
3cjp	8	0.83	0.71	0.71	0.83	0.71	0.7

3c70	8	0.83	0.83	0.79	0.83	0.83	0.79
3brn	8	0.83	0.62	0.63	0.83	0.62	0.63
2rgq	8	0.83	0.71	0.71	0.83	0.71	0.71
2r3e	8	0.83	0.71	0.7	0.83	0.71	0.7
2qid	8	0.83	1.0	0.89	0.83	1.0	0.89
2jw1	8	0.83	0.71	0.7	0.83	0.71	0.7
2jp2	8	0.83	0.71	0.7	0.83	0.71	0.7
2hq7	8	0.83	0.83	0.79	1.0	1.0	1.0
2f01	8	0.83	0.62	0.63	0.83	0.62	0.63
2esr	8	0.83	0.71	0.71	0.83	0.71	0.7
2cm9	8	0.83	0.62	0.63	1.0	1.0	1.0
2ciu	8	0.83	0.83	0.79	1.0	1.0	1.0
2bt9	8	0.83	0.83	0.79	0.83	0.83	0.79
2axw	8	0.83	0.62	0.63	0.83	0.62	0.63
1wfj	8	0.83	0.71	0.7	0.83	0.71	0.7
1vkk	8	0.83	0.83	0.79	1.0	1.0	1.0
1pb0	8	0.83	0.71	0.71	0.83	0.71	0.71
1p4l	8	0.83	0.83	0.79	0.83	0.83	0.79
1n55	8	0.83	0.62	0.64	0.83	0.62	0.64
1mm5	8	0.83	0.62	0.63	0.83	0.62	0.63
1l8j	8	0.83	0.71	0.7	1.0	1.0	1.0
1g90	8	0.83	0.62	0.63	1.0	0.75	0.83
1duj	8	0.83	0.83	0.79	0.83	0.83	0.79
1whk	8	0.8	0.67	0.67	0.8	0.67	0.67
1pp0	8	0.8	0.67	0.67	0.83	0.83	0.79
3duw	8	0.67	0.57	0.51	0.67	0.57	0.5

3d1m	8	0.67	0.67	0.58	0.67	0.67	0.58
3c8i	8	0.67	0.67	0.58	0.83	0.83	0.79
3ba3	8	0.67	0.57	0.5	0.67	0.57	0.5
2yw4	8	0.67	0.57	0.51	0.67	0.57	0.51
2vuv	8	0.67	0.67	0.58	0.67	0.67	0.58
2vpt	8	0.67	0.67	0.58	0.67	0.67	0.58
2vne	8	0.67	0.57	0.5	0.83	0.71	0.7
2vf2	8	0.67	0.57	0.51	0.67	0.57	0.5
2v36	8	0.67	0.8	0.67	0.67	0.8	0.67
2rk9	8	0.67	0.57	0.5	0.83	0.71	0.7
2rb8	8	0.67	0.67	0.58	0.67	0.67	0.58
2r6u	8	0.67	0.57	0.5	0.67	0.57	0.5
2qyz	8	0.67	0.67	0.58	0.83	0.83	0.79
2qnt	8	0.67	0.67	0.58	0.67	0.67	0.58
2ou4	8	0.67	0.5	0.45	0.67	0.5	0.45
2oq8	8	0.67	0.57	0.5	0.67	0.57	0.5
2isv	8	0.67	0.5	0.45	0.67	0.5	0.45
2iml	8	0.67	0.57	0.5	0.83	0.71	0.7
2gm2	8	0.67	0.67	0.58	0.83	0.83	0.79
2glu	8	0.67	0.57	0.5	0.67	0.57	0.5
2fk9	8	0.67	0.67	0.58	0.83	0.83	0.79
2fck	8	0.67	0.57	0.51	0.67	0.57	0.5
2f6x	8	0.67	0.5	0.45	0.67	0.5	0.45
2e3v	8	0.67	0.67	0.58	0.83	0.83	0.79
2c8j	8	0.67	0.67	0.58	0.83	0.83	0.79
2bhh	8	0.67	0.67	0.58	0.83	0.83	0.79

2ayt	8	0.67	0.57	0.5	0.83	0.71	0.7
1wmy	8	0.67	0.57	0.5	0.67	0.57	0.5
1wfu	8	0.67	0.67	0.58	0.67	0.67	0.58
1vrr	8	0.67	0.67	0.58	0.67	0.67	0.58
1vkw	8	0.67	0.67	0.58	0.67	0.67	0.58
1ufo	8	0.67	0.57	0.5	0.67	0.57	0.5
1ucd	8	0.67	0.67	0.58	0.67	0.67	0.58
1u8s	8	0.67	0.67	0.58	0.67	0.67	0.58
1tqj	8	0.67	0.57	0.51	0.67	0.57	0.5
1sj5	8	0.67	0.57	0.51	0.83	0.71	0.7
1s20	8	0.67	0.67	0.58	0.67	0.67	0.58
1rz2	8	0.67	0.5	0.45	0.83	0.62	0.63
1rtt	8	0.67	0.67	0.58	0.83	0.83	0.79
1r77	8	0.67	0.67	0.58	1.0	1.0	1.0
1ql0	8	0.67	0.67	0.58	1.0	1.0	1.0
1qdd	8	0.67	0.57	0.5	1.0	0.86	0.9
1q0z	8	0.67	0.57	0.51	0.67	0.57	0.5
1nki	8	0.67	0.67	0.58	0.83	0.83	0.79
1mj5	8	0.67	0.57	0.5	0.67	0.57	0.5
1im3	8	0.67	0.67	0.58	1.0	1.0	1.0
1hlg	8	0.67	0.57	0.51	0.67	0.57	0.5
1h56	8	0.67	0.8	0.67	0.67	0.8	0.67
1g5q	8	0.67	0.67	0.58	0.67	0.67	0.58
1fro	8	0.67	0.67	0.58	0.83	0.83	0.79
1ecs	8	0.67	0.67	0.58	0.67	0.67	0.58
1dqz	8	0.67	0.57	0.5	0.67	0.57	0.5

1ddw	8	0.67	0.57	0.5	0.83	0.71	0.7
1914	8	0.67	0.57	0.5	0.83	0.71	0.7
1s40	8	0.6	0.5	0.44	0.83	0.83	0.79
3d6j	8	0.5	0.5	0.38	0.5	0.5	0.37
3d1l	8	0.5	0.5	0.37	0.67	0.67	0.58
3bii	8	0.5	0.5	0.36	0.5	0.5	0.36
3bdw	8	0.5	0.5	0.36	0.67	0.67	0.58
2z6w	8	0.5	0.43	0.31	0.67	0.57	0.5
2z2s	8	0.5	0.5	0.36	0.83	0.83	0.79
2vg4	8	0.5	0.5	0.37	0.6	0.5	0.44
2sns	8	0.5	0.6	0.44	0.67	0.8	0.67
2rla	8	0.5	0.43	0.31	0.5	0.43	0.3
2qru	8	0.5	0.43	0.31	0.67	0.57	0.51
2qmo	8	0.5	0.5	0.37	0.5	0.5	0.37
2q5c	8	0.5	0.5	0.38	0.5	0.5	0.37
2q04	8	0.5	0.43	0.32	0.67	0.57	0.51
2pcs	8	0.5	0.43	0.3	0.67	0.57	0.5
2nv4	8	0.5	0.43	0.31	0.5	0.43	0.3
2jwy	8	0.5	0.5	0.36	0.67	0.67	0.58
2hth	8	0.5	0.43	0.31	0.67	0.57	0.51
2fbv	8	0.5	0.5	0.37	0.83	0.83	0.79
2dwx	8	0.5	0.5	0.36	0.67	0.67	0.58
2cov	8	0.5	0.5	0.36	0.67	0.67	0.58
2chh	8	0.5	0.43	0.3	0.67	0.57	0.5
2bwq	8	0.5	0.5	0.36	0.67	0.67	0.58
2aca	8	0.5	0.43	0.3	0.5	0.43	0.3

1zhv	8	0.5	0.43	0.3	0.5	0.43	0.3
1z6h	8	0.5	0.5	0.36	0.5	0.5	0.36
1z0n	8	0.5	0.5	0.36	0.67	0.67	0.58
1y12	8	0.5	0.43	0.3	0.83	0.71	0.7
1xvl	8	0.5	0.6	0.44	0.67	0.8	0.67
1xq4	8	0.5	0.5	0.36	0.67	0.67	0.58
1xm0	8	0.5	0.6	0.44	0.67	0.8	0.67
1x5x	8	0.5	0.5	0.36	0.67	0.67	0.58
1vyn	8	0.5	0.5	0.36	0.83	0.83	0.79
1vrl	8	0.5	0.5	0.36	0.5	0.5	0.36
1vhh	8	0.5	0.5	0.36	0.67	0.67	0.58
1uow	8	0.5	0.5	0.36	0.67	0.67	0.58
1u83	8	0.5	0.43	0.32	0.67	0.57	0.51
1u69	8	0.5	0.5	0.36	0.5	0.5	0.36
1tvg	8	0.5	0.43	0.3	0.67	0.57	0.5
1q3t	8	0.5	0.5	0.36	0.5	0.5	0.37
1q2y	8	0.5	0.43	0.32	0.67	0.57	0.51
1osy	8	0.5	0.5	0.36	0.83	0.83	0.79
1on4	8	0.5	0.5	0.37	0.83	0.83	0.79
1lg7	8	0.5	0.5	0.36	0.67	0.67	0.58
1jc5	8	0.5	0.43	0.31	0.67	0.57	0.5
1i4w	8	0.5	0.43	0.31	0.67	0.57	0.51
1i4v	8	0.5	0.6	0.44	0.5	0.6	0.44
1fm5	8	0.5	0.43	0.31	0.67	0.57	0.5
1f9y	8	0.5	0.5	0.37	0.5	0.5	0.37
1cl3	8	0.5	0.5	0.37	0.67	0.67	0.58

1cjk	8	0.5	0.43	0.3	0.5	0.43	0.3
1bow	8	0.5	0.43	0.3	0.67	0.57	0.5
1b98	8	0.5	0.6	0.44	0.83	0.71	0.7
1avq	8	0.5	0.5	0.36	0.5	0.5	0.36
3dn7	8	0.33	0.33	0.15	0.33	0.33	0.15
3bfq	8	0.33	0.33	0.15	0.33	0.33	0.15
2vsd	8	0.33	0.33	0.15	0.67	0.67	0.58
2v72	8	0.33	0.33	0.15	0.5	0.5	0.36
2v33	8	0.33	0.33	0.15	0.5	0.5	0.36
2tps	8	0.33	0.25	0.092	0.5	0.38	0.26
2r8e	8	0.33	0.33	0.17	0.5	0.5	0.38
2qaz	8	0.33	0.33	0.16	0.5	0.5	0.36
2p7o	8	0.33	0.33	0.15	0.5	0.5	0.36
2nnf	8	0.33	0.33	0.15	0.33	0.33	0.15
2c2i	8	0.33	0.33	0.15	0.33	0.33	0.15
1y7x	8	0.33	0.4	0.21	0.33	0.4	0.21
1xbd	8	0.33	0.33	0.15	0.5	0.5	0.36
1wb4	8	0.33	0.29	0.12	0.33	0.29	0.11
1vi3	8	0.33	0.33	0.15	0.5	0.5	0.36
1vgg	8	0.33	0.33	0.15	0.33	0.33	0.15
1u7e	8	0.33	0.33	0.15	0.5	0.5	0.36
1tvj	8	0.33	0.29	0.1	0.67	0.67	0.58
1tlj	8	0.33	0.33	0.15	0.5	0.5	0.36
1ro7	8	0.33	0.33	0.16	0.5	0.5	0.37
1rkj	8	0.33	0.33	0.15	0.5	0.5	0.36
1oh0	8	0.33	0.29	0.1	0.5	0.43	0.3

1m4l	8	0.33	0.29	0.11	0.67	0.57	0.5
1ls1	8	0.33	0.29	0.11	0.5	0.43	0.3
1g21	8	0.33	0.29	0.11	0.33	0.29	0.11
1adw	8	0.33	0.33	0.16	0.5	0.5	0.36
1a6x	8	0.33	0.33	0.15	0.5	0.5	0.36
2ret	9	1.0	1.0	1.0	1.0	1.0	1.0
2bo9	9	1.0	1.0	1.0	1.0	1.0	1.0
1vju	9	1.0	1.0	1.0	1.0	0.86	0.91
2v3g	9	0.86	0.75	0.75	0.86	0.75	0.75
2p0y	9	0.86	0.86	0.82	0.86	0.86	0.82
2ovi	9	0.86	0.86	0.82	1.0	1.0	1.0
2nn5	9	0.86	0.86	0.82	1.0	1.0	1.0
2jkf	9	0.86	0.86	0.82	0.86	0.86	0.82
1wk0	9	0.86	1.0	0.91	0.86	1.0	0.91
1w1h	9	0.86	0.75	0.75	1.0	0.88	0.92
1kt6	9	0.86	0.67	0.69	1.0	0.78	0.85
1jzu	9	0.86	0.67	0.69	1.0	1.0	1.0
1i1w	9	0.86	0.67	0.69	0.86	0.67	0.69
1epb	9	0.86	0.67	0.69	0.86	0.67	0.69
1njh	9	0.83	0.83	0.8	1.0	1.0	1.0
3e5d	9	0.71	0.71	0.65	0.71	0.71	0.65
3d81	9	0.71	0.71	0.65	0.71	0.71	0.65
3cyg	9	0.71	0.71	0.65	0.83	0.71	0.72
3by9	9	0.71	0.71	0.65	0.71	0.71	0.65
3bhy	9	0.71	0.83	0.72	0.86	0.86	0.82
3bgd	9	0.71	0.62	0.58	0.86	0.75	0.75

2zex	9	0.71	0.62	0.58	0.86	0.75	0.75
2z4d	9	0.71	0.83	0.72	0.71	0.83	0.72
2vdu	9	0.71	0.71	0.65	0.71	0.71	0.65
2r11	9	0.71	0.62	0.58	0.86	0.75	0.75
2qv8	9	0.71	0.71	0.65	0.86	0.86	0.82
2qol	9	0.71	0.83	0.72	0.71	0.83	0.72
2q3w	9	0.71	0.83	0.72	0.71	0.83	0.72
2q03	9	0.71	0.56	0.53	0.71	0.56	0.53
2otd	9	0.71	0.62	0.58	0.71	0.62	0.58
2o6m	9	0.71	1.0	0.82	0.71	1.0	0.82
2hj9	9	0.71	0.71	0.65	0.86	0.86	0.82
2hd9	9	0.71	0.71	0.65	0.86	0.86	0.82
2h1i	9	0.71	0.71	0.65	0.71	0.71	0.65
2gkp	9	0.71	0.71	0.65	0.71	0.71	0.65
2foo	9	0.71	0.71	0.65	0.71	0.71	0.65
2fbl	9	0.71	0.62	0.58	0.86	0.75	0.75
2ex4	9	0.71	0.71	0.65	0.71	0.71	0.65
2efy	9	0.71	0.71	0.65	0.71	0.71	0.65
2e9h	9	0.71	0.83	0.72	0.71	0.83	0.72
2cry	9	0.71	0.71	0.65	0.71	0.71	0.65
2au7	9	0.71	0.62	0.58	0.71	0.62	0.58
1wdw	9	0.71	0.62	0.58	0.71	0.62	0.58
1t9g	9	0.71	0.83	0.72	0.71	0.83	0.72
1q3i	9	0.71	0.62	0.58	0.86	0.75	0.75
1ngl	9	0.71	0.56	0.53	0.86	0.67	0.69
1m40	9	0.71	0.83	0.72	0.71	0.83	0.72

1hcf	9	0.71	0.71	0.65	0.71	0.71	0.65
1cl6	9	0.71	0.71	0.65	0.71	0.71	0.65
2dy0	9	0.67	0.67	0.6	0.71	0.83	0.72
1vae	9	0.6	0.6	0.54	0.6	0.6	0.54
2rlq	9	0.57	0.67	0.53	0.57	0.67	0.53
2rli	9	0.57	0.57	0.47	0.71	0.71	0.65
2q2i	9	0.57	0.67	0.53	0.71	0.83	0.72
2php	9	0.57	0.67	0.53	0.71	0.83	0.72
2ofq	9	0.57	0.57	0.47	0.57	0.57	0.47
2o6p	9	0.57	0.5	0.41	0.71	0.62	0.58
2jsy	9	0.57	0.67	0.54	0.57	0.67	0.54
2jqf	9	0.57	0.57	0.47	0.71	0.71	0.65
2jnz	9	0.57	0.57	0.47	0.57	0.57	0.47
2jeu	9	0.57	0.57	0.47	1.0	0.71	0.82
2hzs	9	0.57	0.44	0.37	0.71	0.56	0.53
2gov	9	0.57	0.5	0.41	0.57	0.5	0.41
2fgs	9	0.57	0.57	0.47	0.83	0.71	0.72
2cdm	9	0.57	0.67	0.53	0.71	0.83	0.72
2c47	9	0.57	0.67	0.53	0.71	0.83	0.72
1zof	9	0.57	0.57	0.47	0.67	0.57	0.54
1wc4	9	0.57	0.57	0.47	0.71	0.71	0.65
1vhw	9	0.57	0.44	0.36	0.71	0.56	0.53
1r0b	9	0.57	0.67	0.54	0.71	0.83	0.72
1qtw	9	0.57	0.57	0.47	0.57	0.57	0.47
1mwp	9	0.57	0.67	0.54	0.71	0.83	0.72
1kg0	9	0.57	0.67	0.53	0.67	0.67	0.6

1k5n	9	0.57	0.57	0.47	0.57	0.57	0.47
1k3b	9	0.57	0.4	0.33	0.86	0.6	0.64
1jsg	9	0.57	0.44	0.36	0.86	0.67	0.69
1hfh	9	0.57	0.67	0.53	0.57	0.67	0.53
1gci	9	0.57	0.57	0.47	0.57	0.57	0.47
1fx4	9	0.57	0.57	0.47	0.71	0.71	0.65
1eso	9	0.57	0.57	0.47	0.57	0.57	0.47
1cr4	9	0.57	0.5	0.41	0.71	0.62	0.58
1c4t	9	0.57	0.57	0.47	0.71	0.71	0.65
1byl	9	0.57	0.57	0.47	0.57	0.57	0.47
1ozv	9	0.5	0.6	0.47	0.5	0.6	0.47
1gxy	9	0.5	0.5	0.4	0.67	0.67	0.6
3d5n	9	0.43	0.43	0.29	0.43	0.43	0.29
3bf7	9	0.43	0.43	0.3	0.5	0.43	0.35
3b9g	9	0.43	0.38	0.25	0.57	0.5	0.42
2z0u	9	0.43	0.43	0.29	0.57	0.57	0.47
2yrb	9	0.43	0.43	0.29	0.43	0.43	0.29
2qwv	9	0.43	0.43	0.3	0.43	0.43	0.3
2qh9	9	0.43	0.43	0.29	0.71	0.71	0.65
2q6m	9	0.43	0.5	0.35	0.43	0.5	0.35
2puk	9	0.43	0.5	0.35	0.83	0.83	0.8
2pof	9	0.43	0.43	0.29	0.57	0.57	0.47
2pk9	9	0.43	0.5	0.35	0.67	0.67	0.6
2p2e	9	0.43	0.43	0.29	0.43	0.43	0.29
2p1d	9	0.43	0.43	0.29	0.57	0.57	0.47
2j9l	9	0.43	0.5	0.35	0.57	0.67	0.54

2izv	9	0.43	0.5	0.35	0.57	0.67	0.54
2isb	9	0.43	0.38	0.26	0.57	0.5	0.42
2ia5	9	0.43	0.43	0.3	0.57	0.57	0.47
2gzq	9	0.43	0.43	0.3	0.71	0.71	0.65
2gzo	9	0.43	0.5	0.35	0.57	0.67	0.53
2gu9	9	0.43	0.43	0.29	0.43	0.43	0.29
2fsd	9	0.43	0.38	0.25	0.57	0.5	0.42
2f7c	9	0.43	0.38	0.24	0.43	0.38	0.24
2f3i	9	0.43	0.38	0.24	0.57	0.5	0.41
2dvk	9	0.43	0.43	0.29	0.57	0.57	0.47
2djm	9	0.43	0.43	0.29	0.71	0.71	0.65
2d13	9	0.43	0.43	0.3	0.57	0.57	0.47
2cjt	9	0.43	0.43	0.29	0.57	0.57	0.47
1ztp	9	0.43	0.38	0.24	0.57	0.5	0.41
1yyc	9	0.43	0.5	0.35	0.57	0.67	0.53
1ypq	9	0.43	0.38	0.25	0.57	0.5	0.41
1y9q	9	0.43	0.43	0.3	0.57	0.57	0.47
1y30	9	0.43	0.43	0.29	0.43	0.43	0.29
1xhu	9	0.43	0.43	0.29	0.57	0.57	0.47
1wui	9	0.43	0.5	0.35	0.5	0.5	0.4
1wfm	9	0.43	0.43	0.29	0.57	0.57	0.47
1vsq	9	0.43	0.43	0.29	0.43	0.43	0.29
1vh3	9	0.43	0.43	0.29	0.57	0.57	0.47
1v70	9	0.43	0.43	0.29	0.43	0.43	0.29
1t9z	9	0.43	0.5	0.35	0.43	0.5	0.35
1n3j	9	0.43	0.5	0.35	0.43	0.5	0.35

1mfm	9	0.43	0.43	0.3	0.57	0.57	0.47
1l0a	9	0.43	0.43	0.3	0.57	0.57	0.47
1k5j	9	0.43	0.43	0.29	0.43	0.43	0.29
1edy	9	0.43	0.43	0.29	0.43	0.43	0.29
1cvj	9	0.43	0.5	0.35	0.5	0.5	0.4
1xdm	9	0.33	0.29	0.18	0.33	0.29	0.17
2vpv	9	0.29	0.29	0.11	0.43	0.43	0.29
2vp1	9	0.29	0.25	0.075	0.29	0.25	0.075
2q7o	9	0.29	0.22	0.041	0.43	0.43	0.29
2p73	9	0.29	0.29	0.11	0.43	0.43	0.29
2jyb	9	0.29	0.25	0.082	0.43	0.38	0.25
2dpl	9	0.29	0.29	0.13	0.29	0.29	0.13
2b82	9	0.29	0.29	0.12	0.43	0.43	0.3
1z3r	9	0.29	0.33	0.16	0.43	0.43	0.3
1r75	9	0.29	0.29	0.11	0.43	0.43	0.29
1qc9	9	0.29	0.33	0.17	0.43	0.5	0.35
1k7j	9	0.29	0.29	0.11	0.43	0.43	0.29
2jzj	10	1.0	1.0	1.0	1.0	1.0	1.0
1x8q	10	1.0	0.8	0.87	1.0	0.8	0.87
1vay	10	1.0	0.88	0.92	1.0	0.88	0.92
2q4n	10	0.88	0.7	0.73	1.0	0.8	0.87
2hhi	10	0.88	0.88	0.85	0.88	0.88	0.85
2g7b	10	0.88	0.78	0.79	1.0	0.89	0.93
2anu	10	0.88	0.78	0.78	0.88	0.78	0.78
1us0	10	0.88	0.88	0.85	0.88	0.88	0.85
1tnr	10	0.86	1.0	0.91	0.86	1.0	0.91

1tba	10	0.88	0.78	0.78	0.88	0.78	0.78
1rfv	10	0.88	0.78	0.79	1.0	0.89	0.93
1mdc	10	0.88	0.78	0.78	0.88	0.78	0.78
1jhs	10	0.88	0.88	0.85	0.88	0.88	0.85
1ifc	10	0.88	0.78	0.79	1.0	0.89	0.93
1gwe	10	0.88	0.7	0.73	0.88	0.7	0.73
3bs1	10	0.86	0.86	0.83	0.86	0.86	0.83
2rdk	10	0.86	1.0	0.91	0.86	1.0	0.91
2jzl	10	0.86	1	0.91	0.86	1.0	0.91
3bp1	10	0.75	0.75	0.7	0.75	0.75	0.7
2h9a	10	0.75	0.75	0.7	0.75	0.75	0.7
2g3r	10	0.75	0.75	0.7	0.88	0.88	0.85
2cg7	10	0.75	1.0	0.84	0.75	1.0	0.84
2bkd	10	0.75	0.75	0.7	0.75	0.75	0.7
2b7u	10	0.75	0.86	0.76	0.88	0.86	0.82
1ydo	10	0.75	0.67	0.64	0.75	0.67	0.64
1wv4	10	0.75	0.67	0.64	0.88	0.78	0.78
1lf7	10	0.75	0.6	0.59	0.88	0.7	0.73
1ag4	10	0.75	0.6	0.59	0.88	0.7	0.73
2a0m	10	0.71	0.62	0.6	0.71	0.62	0.6
1obo	10	0.71	0.62	0.6	0.71	0.62	0.6
7a3h	10	0.62	0.56	0.5	0.62	0.56	0.5
3c0y	10	0.62	0.62	0.55	0.75	0.86	0.76
3bwh	10	0.62	0.71	0.6	0.75	0.86	0.76
2z5c	10	0.62	0.62	0.54	0.62	0.62	0.54
2z5c	10	0.62	0.62	0.54	0.62	0.62	0.54

2z5c	10	0.62	0.62	0.54	0.62	0.62	0.54
1euw	10	0.62	0.62	0.54	0.62	0.62	0.54
2ra6	10	0.62	0.56	0.49	0.62	0.56	0.49
2ovs	10	0.62	0.5	0.45	0.62	0.5	0.45
2nwf	10	0.62	0.71	0.6	0.75	0.86	0.76
2nrh	10	0.62	0.62	0.55	0.62	0.62	0.55
2i3b	10	0.62	0.56	0.49	0.62	0.56	0.49
2g8o	10	0.62	0.71	0.61	0.75	0.86	0.76
2cwp	10	0.62	0.71	0.6	0.71	0.71	0.66
1zhi	10	0.62	0.56	0.5	0.62	0.56	0.5
1zcw	10	0.62	0.5	0.46	0.75	0.6	0.59
1x3w	10	0.62	0.71	0.6	0.62	0.71	0.6
1vqt	10	0.62	0.56	0.5	0.71	0.56	0.56
1vli	10	0.62	0.71	0.6	0.62	0.71	0.6
1v2b	10	0.62	0.62	0.54	0.88	0.88	0.85
1t15	10	0.62	0.71	0.6	0.88	0.88	0.85
1rzn	10	0.62	0.71	0.6	0.62	0.71	0.6
1rz0	10	0.62	0.71	0.6	0.62	0.71	0.6
1rg8	10	0.62	0.71	0.6	0.62	0.71	0.6
1qlw	10	0.62	0.62	0.55	0.62	0.62	0.54
1p1x	10	0.62	0.62	0.55	0.62	0.62	0.55
1m15	10	0.62	0.62	0.54	0.88	0.88	0.85
1jls	10	0.62	0.62	0.54	0.62	0.62	0.54
1jd2	10	0.62	0.62	0.54	0.88	0.88	0.85
1ix5	10	0.62	0.83	0.67	0.62	0.83	0.67
1hux	10	0.62	0.62	0.54	0.62	0.62	0.55

1dzk	10	0.62	0.56	0.49	0.62	0.56	0.49
1dv9	10	0.62	0.56	0.49	0.62	0.56	0.49
2opl	10	0.57	0.67	0.55	0.71	0.83	0.73
3djm	10	0.5	0.57	0.44	0.5	0.57	0.44
3bb1	10	0.5	0.57	0.44	0.71	0.71	0.66
2z64	10	0.5	0.5	0.39	0.5	0.5	0.39
2r5o	10	0.5	0.5	0.39	0.5	0.5	0.39
2qfd	10	0.5	0.57	0.44	0.75	0.86	0.76
2ite	10	0.5	0.5	0.39	0.62	0.62	0.54
2idc	10	0.5	0.5	0.39	0.62	0.62	0.54
2fj8	10	0.5	0.67	0.5	0.62	0.83	0.67
2e7d	10	0.5	0.5	0.39	0.88	0.88	0.85
2dkt	10	0.5	0.67	0.5	0.57	0.67	0.55
2ckk	10	0.5	0.5	0.39	0.62	0.62	0.54
2b06	10	0.5	0.5	0.39	0.5	0.5	0.39
1vch	10	0.5	0.57	0.45	0.5	0.57	0.44
1r8y	10	0.5	0.5	0.39	0.62	0.62	0.54
1dqn	10	0.5	0.5	0.39	0.62	0.62	0.54
1qfe	10	0.5	0.5	0.4	0.5	0.5	0.4
1ofq	10	0.5	0.44	0.36	0.5	0.44	0.36
1o4t	10	0.5	0.5	0.39	0.5	0.5	0.39
1ko6	10	0.5	0.57	0.44	0.62	0.62	0.54
1k3x	10	0.5	0.57	0.44	0.5	0.57	0.44
1hdo	10	0.5	0.5	0.39	0.5	0.5	0.39
1h9r	10	0.5	0.5	0.39	0.5	0.5	0.39
1c28	10	0.5	0.5	0.39	0.62	0.62	0.54

1byi	10	0.5	0.57	0.44	0.62	0.71	0.6
1bpr	10	0.5	0.67	0.5	0.5	0.67	0.5
1qwo	10	0.43	0.38	0.28	0.57	0.5	0.44
3cla	10	0.38	0.38	0.24	0.5	0.5	0.39
2zke	10	0.38	0.33	0.21	0.75	0.86	0.76
2zan	10	0.38	0.43	0.28	0.38	0.43	0.28
2qbu	10	0.38	0.38	0.24	0.38	0.38	0.24
2pqf	10	0.38	0.38	0.24	0.5	0.5	0.39
2p86	10	0.38	0.43	0.28	0.62	0.62	0.54
2p0r	10	0.38	0.43	0.28	0.5	0.57	0.44
2orw	10	0.38	0.43	0.28	0.38	0.43	0.28
2hew	10	0.38	0.38	0.24	0.5	0.5	0.39
2gml	10	0.38	0.38	0.24	0.62	0.62	0.54
2enq	10	0.38	0.38	0.24	0.5	0.5	0.39
2co7	10	0.38	0.38	0.24	0.38	0.38	0.24
2azw	10	0.38	0.38	0.24	0.38	0.38	0.24
2arc	10	0.38	0.38	0.24	0.62	0.62	0.54
1zuw	10	0.38	0.38	0.24	0.5	0.5	0.39
1zkk	10	0.38	0.5	0.33	0.38	0.5	0.33
1x5h	10	0.38	0.43	0.28	0.5	0.57	0.44
1wyz	10	0.38	0.43	0.28	0.38	0.43	0.28
1nhe	10	0.38	0.38	0.24	0.38	0.38	0.24
1muw	10	0.38	0.38	0.25	0.5	0.57	0.45
1ijg	10	0.38	0.43	0.29	0.5	0.57	0.45
2qzq	10	0.29	0.25	0.12	0.43	0.38	0.28
2izw	10	0.29	0.25	0.12	0.38	0.38	0.24

1rxt	10	0.29	0.29	0.16	0.29	0.29	0.16
1odf	10	0.29	0.29	0.16	0.43	0.43	0.33
3eqe	10	0.25	0.29	0.12	0.43	0.43	0.33
3cfu	10	0.25	0.29	0.13	0.38	0.43	0.29
2ozz	10	0.25	0.29	0.12	0.62	0.62	0.54
2j2z	10	0.25	0.33	0.16	0.5	0.67	0.5
2heu	10	0.25	0.29	0.12	0.38	0.43	0.28
2afj	10	0.25	0.29	0.12	0.38	0.43	0.28
1zrr	10	0.25	0.29	0.12	0.38	0.43	0.28
1utb	10	0.25	0.29	0.12	0.38	0.43	0.28
1tzp	10	0.25	0.33	0.16	0.38	0.5	0.34
1o67	10	0.25	0.25	0.088	0.25	0.25	0.092
2zf4	11	0.89	0.8	0.81	0.89	0.8	0.81
2aco	11	0.89	0.8	0.81	1.0	0.9	0.94
1lsh	11	0.89	0.8	0.81	0.89	0.8	0.81
2vdf	11	0.88	0.64	0.7	0.88	0.64	0.7
2dxu	11	0.78	0.78	0.74	0.89	0.89	0.87
1xj5	11	0.78	0.78	0.73	0.89	0.89	0.87
1dfm	11	0.78	0.78	0.73	0.78	0.78	0.73
3djc	11	0.75	0.67	0.66	0.89	0.89	0.87
1niv	11	0.75	0.75	0.71	0.75	0.75	0.71
1gg3	11	0.75	0.75	0.71	0.88	0.88	0.85
1h32	11	0.71	0.83	0.74	0.83	0.83	0.81
2p0l	11	0.67	0.67	0.6	0.67	0.67	0.6
1ynm	11	0.67	0.67	0.6	0.78	0.78	0.73
1usc	11	0.67	0.75	0.65	0.67	0.75	0.65

1t5y	11	0.67	0.67	0.6	0.78	0.78	0.73
1eys	11	0.67	0.75	0.65	0.67	0.75	0.65
1ee9	11	0.67	0.67	0.6	0.67	0.67	0.6
1aur	11	0.67	0.75	0.65	0.78	0.88	0.79
2rk3	11	0.62	0.62	0.56	0.62	0.62	0.56
3epp	11	0.56	0.56	0.47	0.78	0.78	0.74
2znr	11	0.56	0.56	0.47	0.78	0.78	0.74
2qsd	11	0.56	0.62	0.51	0.56	0.62	0.51
2o3o	11	0.56	0.56	0.47	0.67	0.67	0.6
2jg8	11	0.56	0.56	0.47	0.67	0.67	0.6
2jfr	11	0.56	0.56	0.47	0.56	0.56	0.47
2imq	11	0.56	0.56	0.47	0.67	0.67	0.6
1kmv	11	0.56	0.56	0.47	0.67	0.67	0.6
2fuc	11	0.56	0.56	0.47	0.62	0.56	0.52
2f9b	11	0.56	0.71	0.57	0.56	0.71	0.57
2aj7	11	0.56	0.56	0.47	0.56	0.56	0.47
1ys1	11	0.56	0.62	0.51	0.56	0.62	0.51
1xhh	11	0.56	0.71	0.57	0.78	0.86	0.78
1wl8	11	0.56	0.56	0.47	0.78	0.78	0.73
1uzk	11	0.56	0.83	0.63	0.62	0.83	0.68
1tp8	11	0.56	0.62	0.51	0.56	0.62	0.51
1t6y	11	0.56	0.5	0.43	0.67	0.6	0.56
1t0v	11	0.56	0.71	0.57	0.56	0.71	0.57
1sx8	11	0.56	0.62	0.51	0.56	0.62	0.51
1l6r	11	0.56	0.56	0.47	0.67	0.67	0.6
1h1c	11	0.56	0.56	0.47	0.56	0.56	0.47

1g55	11	0.56	0.56	0.47	0.56	0.56	0.47
1dct	11	0.56	0.62	0.52	0.56	0.62	0.52
2uvg	11	0.5	0.5	0.41	0.5	0.5	0.41
1noz	11	0.5	0.5	0.42	0.5	0.5	0.42
3b93	11	0.44	0.44	0.34	0.44	0.44	0.34
2vrb	11	0.44	0.5	0.38	0.56	0.62	0.51
2iee	11	0.44	0.5	0.38	0.5	0.5	0.41
2bvb	11	0.44	0.44	0.34	0.67	0.67	0.6
1y7m	11	0.44	0.5	0.38	0.44	0.5	0.38
1xkj	11	0.44	0.4	0.31	0.5	0.4	0.35
1w66	11	0.44	0.44	0.34	0.56	0.56	0.47
1qzt	11	0.44	0.44	0.34	0.44	0.44	0.34
1qr0	11	0.44	0.57	0.42	0.5	0.57	0.46
1kqg	11	0.44	0.57	0.42	0.56	0.71	0.57
3bcz	11	0.38	0.33	0.24	0.38	0.33	0.24
1j3g	11	0.38	0.43	0.31	0.38	0.43	0.31
1fsg	11	0.38	0.33	0.24	0.38	0.33	0.24
1eb0	11	0.38	0.38	0.27	0.38	0.38	0.27
3ebr	11	0.33	0.38	0.24	0.33	0.38	0.24
3dal	11	0.33	0.38	0.24	0.33	0.38	0.24
3cne	11	0.33	0.43	0.28	0.38	0.43	0.31
2ywi	11	0.33	0.38	0.24	0.44	0.5	0.38
2uzf	11	0.33	0.33	0.2	0.38	0.33	0.24
2o39	11	0.33	0.43	0.27	0.56	0.71	0.57
2i3c	11	0.33	0.33	0.2	0.44	0.44	0.34
2g6t	11	0.33	0.38	0.24	0.56	0.56	0.47

1znp	11	0.33	0.33	0.2	0.33	0.33	0.2
1zn6	11	0.33	0.38	0.24	0.38	0.38	0.27
1yco	11	0.33	0.33	0.2	0.33	0.33	0.2
1t4w	11	0.33	0.33	0.2	0.44	0.44	0.34
1qu5	11	0.33	0.43	0.28	0.44	0.57	0.42
1m1n	11	0.33	0.38	0.24	0.33	0.38	0.24
1k2n	11	0.33	0.38	0.24	0.33	0.38	0.24
1h6q	11	0.33	0.43	0.28	0.5	0.57	0.46
1gwm	11	0.33	0.33	0.2	0.5	0.57	0.46
1w0n	11	0.25	0.22	0.096	0.25	0.22	0.096
2o8q	11	0.22	0.25	0.096	0.33	0.38	0.24
2mev	11	0.22	0.25	0.096	0.33	0.38	0.24
1zoq	11	0.22	0.22	0.076	0.33	0.33	0.21
1h6x	11	0.22	0.22	0.07	0.56	0.56	0.47
2jep	12	1.0	0.9	0.94	1.0	0.9	0.94
1eal	12	0.9	0.9	0.88	0.9	0.9	0.88
3dha	12	0.8	0.8	0.76	0.8	0.8	0.76
3cbt	12	0.8	0.8	0.76	0.8	0.8	0.76
2r3u	12	0.8	0.67	0.68	0.9	0.75	0.79
1nt2	12	0.8	0.8	0.76	0.89	0.8	0.82
3bb7	12	0.7	0.7	0.65	0.78	0.7	0.69
2vh1	12	0.7	0.7	0.65	0.7	0.7	0.65
2agk	12	0.7	0.64	0.61	0.8	0.73	0.72
1iru	12	0.7	0.78	0.69	0.8	0.89	0.82
2yzs	12	0.67	0.75	0.66	0.67	0.75	0.66
2drz	12	0.67	0.86	0.72	0.67	0.86	0.72

1wbc	12	0.67	0.67	0.61	0.67	0.67	0.61
1knm	12	0.67	0.86	0.72	0.67	0.86	0.72
1hcd	12	0.67	0.86	0.72	0.67	0.86	0.72
1gvm	12	0.67	1.0	0.8	0.67	1.0	0.8
1ft4	12	0.67	1.0	0.8	0.67	1.0	0.8
3bop	12	0.6	0.55	0.49	0.6	0.55	0.49
2qf4	12	0.6	0.5	0.46	0.8	0.67	0.68
2ob3	12	0.6	0.75	0.62	0.6	0.75	0.62
2j5v	12	0.6	0.6	0.53	0.7	0.7	0.65
2e1b	12	0.6	0.67	0.57	0.7	0.78	0.69
2d5m	12	0.6	0.67	0.57	0.6	0.67	0.57
1z8r	12	0.6	0.75	0.62	0.6	0.75	0.62
1pv2	12	0.6	0.67	0.57	0.6	0.67	0.57
1juv	12	0.6	0.6	0.53	0.6	0.6	0.53
1jss	12	0.6	0.6	0.53	0.7	0.7	0.65
2v4m	12	0.56	0.56	0.49	0.56	0.56	0.49
2ckq	12	0.56	0.62	0.53	0.56	0.62	0.53
1vzi	12	0.56	0.62	0.53	0.67	0.75	0.66
1r8n	12	0.56	0.62	0.53	0.67	0.75	0.66
2nn8	12	0.5	0.5	0.41	0.6	0.6	0.53
2hy3	12	0.5	0.56	0.45	0.56	0.56	0.49
2fhh	12	0.5	0.56	0.45	0.5	0.56	0.45
2c37	12	0.5	0.56	0.45	0.5	0.56	0.45
1z0w	12	0.5	0.56	0.45	0.6	0.67	0.57
1xdq	12	0.5	0.5	0.41	0.5	0.5	0.41
1ulc	12	0.5	0.5	0.41	0.5	0.5	0.41

1qkq	12	0.5	0.5	0.41	0.6	0.6	0.53
1is3	12	0.5	0.5	0.41	0.6	0.6	0.53
1em2	12	0.5	0.5	0.41	0.5	0.5	0.41
1b26	12	0.5	0.5	0.41	0.6	0.6	0.53
1r9g	12	0.44	0.5	0.39	0.5	0.62	0.49
1l3k	12	0.44	0.5	0.39	0.44	0.5	0.39
3c26	12	0.4	0.4	0.3	0.4	0.4	0.29
3b7k	12	0.4	0.4	0.29	0.5	0.5	0.41
2j12	12	0.4	0.44	0.32	0.4	0.44	0.32
2fvy	12	0.4	0.44	0.32	0.4	0.44	0.32
2a85	12	0.4	0.5	0.36	0.4	0.5	0.36
1pff	12	0.4	0.4	0.3	0.4	0.4	0.3
1o3s	12	0.4	0.44	0.33	0.5	0.56	0.45
1h03	12	0.4	0.5	0.36	0.4	0.5	0.36
1cr5	12	0.4	0.4	0.29	0.4	0.4	0.29
1bo1	12	0.4	0.4	0.3	0.6	0.6	0.53
2aeg	12	0.33	0.3	0.2	0.44	0.4	0.32
8abp	12	0.3	0.3	0.18	0.33	0.3	0.21
3clk	12	0.3	0.3	0.18	0.4	0.4	0.3
2yuj	12	0.3	0.3	0.17	0.4	0.4	0.29
2vha	12	0.3	0.3	0.17	0.3	0.3	0.17
1vkj	12	0.3	0.38	0.23	0.44	0.5	0.39
1uww	12	0.3	0.38	0.23	0.4	0.5	0.36
1q9g	12	0.3	0.33	0.2	0.4	0.44	0.33
1nmo	12	0.3	0.3	0.18	0.4	0.4	0.3
1fcv	12	0.3	0.43	0.27	0.33	0.43	0.29

2pa7	12	0.22	0.22	0.099	0.33	0.33	0.23
2qlt	12	0.2	0.22	0.083	0.3	0.33	0.21
3znb	13	0.91	0.91	0.89	0.91	0.91	0.89
2z4h	13	0.91	0.91	0.89	1.0	1.0	1.0
1iwn	13	0.82	0.82	0.79	0.91	0.91	0.89
1d7b	13	0.82	0.82	0.79	0.82	0.82	0.79
1yqs	13	0.8	0.8	0.77	0.8	0.8	0.77
2asu	13	0.73	0.62	0.61	0.82	0.69	0.71
1lc7	13	0.73	0.8	0.73	0.8	0.8	0.77
1fuj	13	0.73	0.62	0.61	0.82	0.69	0.71
1xto	13	0.7	0.64	0.62	0.8	0.73	0.73
2vhh	13	0.64	0.7	0.62	0.73	0.8	0.73
2bsz	13	0.64	0.64	0.58	0.64	0.64	0.58
2amp	13	0.64	0.64	0.58	0.73	0.73	0.68
1tlt	13	0.64	0.64	0.58	0.64	0.64	0.58
1im0	13	0.64	0.54	0.51	0.7	0.54	0.55
3c5p	13	0.6	0.6	0.54	0.7	0.64	0.62
2vok	13	0.6	0.55	0.51	0.7	0.64	0.62
2o5n	13	0.6	0.6	0.54	0.7	0.7	0.66
1zwj	13	0.6	0.55	0.51	0.6	0.55	0.51
1j0s	13	0.6	0.67	0.58	0.6	0.67	0.58
1dt9	13	0.6	0.6	0.54	0.6	0.6	0.54
2uu7	13	0.55	0.55	0.47	0.64	0.64	0.58
2rbk	13	0.55	0.6	0.51	0.55	0.6	0.51
2qck	13	0.55	0.6	0.51	0.55	0.6	0.51
2pi1	13	0.55	0.55	0.47	0.55	0.55	0.47

2ezv	13	0.55	0.67	0.55	0.55	0.67	0.55
2a7r	13	0.55	0.55	0.47	0.55	0.55	0.47
1ug6	13	0.55	0.55	0.47	0.55	0.55	0.47
1t8i	13	0.55	0.67	0.55	0.55	0.67	0.55
1nxm	13	0.55	0.67	0.55	0.55	0.67	0.55
1nrx	13	0.55	0.67	0.55	0.6	0.67	0.58
1ehi	13	0.55	0.6	0.51	0.55	0.6	0.51
1e5u	13	0.55	0.67	0.55	0.55	0.67	0.55
1uwc	13	0.5	0.5	0.43	0.6	0.6	0.54
1d7p	13	0.5	0.5	0.43	0.5	0.5	0.43
2ux8	13	0.45	0.45	0.36	0.45	0.45	0.36
2ok7	13	0.45	0.5	0.4	0.55	0.6	0.51
2j0u	13	0.45	0.45	0.36	0.45	0.45	0.36
2e6g	13	0.45	0.5	0.4	0.5	0.5	0.43
2aam	13	0.45	0.56	0.43	0.45	0.56	0.43
1ty2	13	0.45	0.5	0.4	0.45	0.5	0.4
1lhv	13	0.45	0.45	0.36	0.55	0.55	0.47
1hys	13	0.45	0.56	0.43	0.5	0.56	0.46
1es3	13	0.45	0.62	0.47	0.45	0.62	0.47
7i1b	13	0.4	0.44	0.34	0.45	0.56	0.43
1x82	13	0.4	0.44	0.34	0.45	0.56	0.43
1ro2	13	0.4	0.4	0.31	0.5	0.5	0.43
1ef7	13	0.4	0.4	0.31	0.4	0.4	0.31
1b04	13	0.4	0.44	0.34	0.45	0.56	0.43
1mr9	13	0.38	0.33	0.27	0.38	0.33	0.27
3seb	13	0.36	0.44	0.31	0.36	0.44	0.32

2ic1	13	0.36	0.4	0.29	0.4	0.4	0.31
2c20	13	0.36	0.44	0.32	0.36	0.44	0.32
2a8i	13	0.36	0.36	0.26	0.45	0.45	0.37
1uz0	13	0.36	0.44	0.31	0.36	0.44	0.31
1g88	13	0.36	0.36	0.26	0.55	0.55	0.47
2r2j	13	0.3	0.33	0.22	0.3	0.33	0.22
2d3m	13	0.3	0.3	0.2	0.4	0.4	0.31
2c0z	13	0.3	0.3	0.2	0.3	0.3	0.2
1xe8	13	0.3	0.3	0.2	0.4	0.4	0.31
1n7g	13	0.3	0.33	0.22	0.4	0.44	0.34
3ckh	13	0.27	0.27	0.15	0.36	0.36	0.26
2o51	13	0.27	0.3	0.18	0.3	0.3	0.2
2cy7	13	0.27	0.3	0.18	0.36	0.4	0.29
1wck	13	0.27	0.3	0.18	0.36	0.4	0.29
1odm	13	0.27	0.27	0.16	0.36	0.36	0.26
2jxy	14	0.91	1.0	0.95	0.91	1.0	0.95
1ww1	14	0.73	0.67	0.65	0.73	0.67	0.65
1poi	14	0.73	0.73	0.69	0.73	0.73	0.69
1imd	14	0.73	0.73	0.69	0.73	0.73	0.69
3b5u	14	0.7	0.64	0.62	0.7	0.64	0.62
2hev	14	0.7	1.0	0.82	0.7	1.0	0.82
1i78	14	0.7	0.47	0.51	0.82	0.6	0.65
2okt	14	0.67	0.73	0.65	0.67	0.73	0.65
2ho5	14	0.67	0.67	0.62	0.67	0.67	0.62
2e2x	14	0.67	0.73	0.65	0.75	0.82	0.75
1s31	14	0.67	0.62	0.58	0.83	0.77	0.77

1pq7	14	0.67	0.62	0.58	0.83	0.77	0.77
1o7j	14	0.67	0.73	0.65	0.73	0.73	0.69
1kyf	14	0.67	0.73	0.65	0.67	0.73	0.65
2yug	14	0.64	0.78	0.67	0.64	0.78	0.67
1t2d	14	0.64	0.7	0.62	0.64	0.7	0.62
1okg	14	0.6	0.6	0.55	0.6	0.6	0.55
4mdh	14	0.58	0.7	0.59	0.64	0.7	0.62
3efh	14	0.58	0.64	0.55	0.64	0.64	0.59
2f1v	14	0.58	0.54	0.49	0.67	0.62	0.58
2bt8	14	0.58	0.58	0.52	0.58	0.58	0.52
1y8q	14	0.58	0.58	0.52	0.58	0.58	0.52
1vyr	14	0.58	0.64	0.55	0.64	0.64	0.59
1qa7	14	0.58	0.58	0.52	0.58	0.58	0.52
3bdz	14	0.55	0.67	0.55	0.55	0.67	0.55
2abs	14	0.55	0.5	0.45	0.55	0.5	0.45
1w23	14	0.55	0.6	0.52	0.55	0.6	0.52
1vmo	14	0.55	0.55	0.48	0.55	0.55	0.48
1fze	14	0.55	0.55	0.48	0.6	0.55	0.52
3eaf	14	0.5	0.5	0.42	0.5	0.5	0.43
3d02	14	0.5	0.55	0.46	0.55	0.55	0.49
2q6l	14	0.5	0.5	0.42	0.58	0.58	0.52
1qop	14	0.5	0.55	0.45	0.5	0.55	0.45
1qfc	14	0.5	0.5	0.42	0.55	0.5	0.45
1oh4	14	0.5	0.5	0.42	0.58	0.58	0.52
1mzj	14	0.5	0.6	0.49	0.67	0.8	0.69
1ka1	14	0.5	0.55	0.45	0.58	0.64	0.55

1f7v	14	0.5	0.5	0.42	0.58	0.58	0.52
1iah	14	0.5	0.55	0.45	0.5	0.55	0.45
1z70	14	0.45	0.56	0.44	0.5	0.56	0.47
1w97	14	0.45	0.5	0.41	0.45	0.5	0.41
1tjy	14	0.45	0.45	0.38	0.45	0.45	0.38
1rut	14	0.45	0.56	0.44	0.45	0.56	0.44
1pw5	14	0.45	0.56	0.44	0.5	0.56	0.47
2f5t	14	0.42	0.38	0.31	0.42	0.38	0.31
2bp7	14	0.42	0.45	0.35	0.5	0.55	0.45
1zl0	14	0.42	0.42	0.33	0.42	0.42	0.33
1z2n	14	0.42	0.45	0.35	0.42	0.45	0.35
1m4v	14	0.42	0.45	0.35	0.5	0.55	0.45
1fjm	14	0.42	0.45	0.35	0.45	0.45	0.38
1fg9	14	0.42	0.5	0.38	0.42	0.5	0.38
2gsh	14	0.36	0.36	0.28	0.36	0.36	0.28
2r84	14	0.33	0.4	0.28	0.42	0.5	0.38
2nxv	14	0.33	0.36	0.25	0.36	0.36	0.28
1t94	14	0.33	0.4	0.28	0.42	0.5	0.38
1hwh	14	0.33	0.4	0.28	0.33	0.4	0.28
1h4a	14	0.33	0.4	0.28	0.33	0.4	0.28
1g43	14	0.33	0.4	0.28	0.42	0.5	0.38
2q89	14	0.3	0.33	0.24	0.4	0.44	0.35
1pkh	14	0.25	0.3	0.18	0.42	0.5	0.38
1lkx	14	0.25	0.3	0.17	0.36	0.4	0.3
1i24	14	0.25	0.3	0.18	0.25	0.3	0.17
1pqz	15	0.77	0.83	0.77	0.85	0.92	0.86

1llf	15	0.75	0.75	0.72	0.75	0.75	0.72
3bsh	15	0.69	0.69	0.65	0.75	0.69	0.68
1g61	15	0.67	0.8	0.7	0.67	0.8	0.7
2vvg	15	0.62	0.67	0.59	0.62	0.67	0.59
2fdm	15	0.62	0.57	0.53	0.62	0.57	0.53
2ddx	15	0.6	0.46	0.47	0.64	0.54	0.53
3d2u	15	0.58	0.58	0.53	0.67	0.67	0.62
1u2d	15	0.58	0.58	0.53	0.67	0.67	0.62
1fl0	15	0.58	0.7	0.6	0.73	0.8	0.74
1cy0	15	0.58	0.58	0.53	0.58	0.58	0.53
3dlq	15	0.54	0.64	0.53	0.54	0.64	0.53
3db2	15	0.54	0.54	0.47	0.54	0.54	0.47
2erj	15	0.54	0.64	0.53	0.69	0.82	0.72
2eo5	15	0.54	0.58	0.5	0.58	0.58	0.53
2e48	15	0.54	0.54	0.47	0.54	0.54	0.47
2ci1	15	0.54	0.7	0.57	0.54	0.7	0.57
1zde	15	0.54	0.7	0.57	0.54	0.7	0.57
1vdw	15	0.54	0.58	0.5	0.62	0.67	0.59
1pb7	15	0.54	0.58	0.5	0.62	0.67	0.59
1nb8	15	0.54	0.58	0.5	0.58	0.58	0.53
1iar	15	0.54	0.64	0.53	0.58	0.64	0.56
1ern	15	0.54	0.64	0.53	0.62	0.73	0.63
2rir	15	0.5	0.5	0.44	0.5	0.5	0.44
2an6	15	0.5	0.55	0.46	0.5	0.55	0.46
1r5y	15	0.5	0.55	0.47	0.5	0.55	0.47
3cea	15	0.46	0.5	0.41	0.46	0.5	0.41

2r16	15	0.46	0.46	0.39	0.5	0.46	0.41
2hqy	15	0.46	0.46	0.39	0.46	0.46	0.39
2ba0	15	0.46	0.5	0.41	0.46	0.5	0.41
1un1	15	0.46	0.46	0.39	0.46	0.46	0.39
1gnh	15	0.46	0.46	0.39	0.46	0.46	0.39
1at0	15	0.46	0.67	0.5	0.46	0.67	0.5
1mdz	15	0.45	0.5	0.42	0.5	0.5	0.45
2z20	15	0.42	0.5	0.39	0.5	0.6	0.5
2epk	15	0.42	0.45	0.37	0.5	0.55	0.46
2cnq	15	0.42	0.45	0.37	0.42	0.45	0.37
1g5x	15	0.42	0.42	0.34	0.42	0.42	0.34
2iw1	15	0.38	0.42	0.32	0.38	0.42	0.32
2a6z	15	0.38	0.38	0.3	0.46	0.46	0.39
1n8b	15	0.38	0.45	0.34	0.38	0.45	0.34
2qik	15	0.31	0.36	0.25	0.38	0.45	0.34
1nkq	15	0.31	0.33	0.23	0.31	0.33	0.23
1dcs	15	0.31	0.33	0.23	0.38	0.42	0.32
2d7s	15	0.23	0.3	0.17	0.23	0.3	0.17
3buu	16	0.79	0.85	0.79	0.86	0.92	0.88
1jl0	16	0.75	0.64	0.66	0.75	0.64	0.66
1yln	16	0.71	0.67	0.65	0.71	0.67	0.65
2ri2	16	0.69	0.69	0.65	0.69	0.69	0.65
3co8	16	0.64	0.75	0.66	0.69	0.75	0.69
1xmx	16	0.64	0.69	0.63	0.69	0.69	0.66
2ffy	16	0.57	0.67	0.57	0.64	0.75	0.66
1hl9	16	0.57	0.57	0.52	0.57	0.57	0.52

1hf2	16	0.54	0.54	0.48	0.62	0.62	0.57
2e6f	16	0.5	0.5	0.45	0.5	0.5	0.45
1pfv	16	0.5	0.64	0.51	0.5	0.64	0.51
1su8	16	0.46	0.5	0.42	0.46	0.5	0.42
2uu8	16	0.43	0.43	0.35	0.5	0.5	0.43
2nv9	16	0.43	0.4	0.34	0.46	0.4	0.36
2cws	16	0.43	0.43	0.35	0.5	0.5	0.43
1vho	16	0.43	0.46	0.37	0.46	0.46	0.4
1uwk	16	0.43	0.46	0.38	0.43	0.46	0.38
1orh	16	0.43	0.5	0.4	0.5	0.58	0.49
1jey	16	0.43	0.46	0.38	0.43	0.46	0.38
1gv9	16	0.43	0.43	0.35	0.43	0.43	0.35
2ggc	16	0.38	0.45	0.35	0.38	0.45	0.36
1pmh	16	0.36	0.42	0.31	0.36	0.42	0.31
1ds1	16	0.31	0.33	0.24	0.31	0.33	0.24
2bhv	16	0.29	0.33	0.23	0.29	0.33	0.23
2odi	16	0.23	0.27	0.17	0.31	0.36	0.26
1k5n	17	0.79	0.79	0.76	0.79	0.79	0.76
1itv	17	0.79	0.85	0.8	0.79	0.85	0.8
2jdx	17	0.71	0.91	0.79	0.71	0.91	0.79
1zt4	17	0.71	0.77	0.71	0.71	0.77	0.71
1p3e	17	0.64	0.69	0.63	0.71	0.77	0.71
2yx1	17	0.6	0.69	0.6	0.6	0.69	0.6
2osx	17	0.6	0.64	0.58	0.6	0.64	0.58
2ewo	17	0.6	0.82	0.67	0.6	0.82	0.67
2eiy	17	0.6	0.6	0.55	0.6	0.6	0.55

1nof	17	0.6	0.56	0.53	0.6	0.56	0.53
2jhf	17	0.57	0.57	0.52	0.57	0.57	0.52
2jgt	17	0.54	0.54	0.49	0.62	0.62	0.58
2ehz	17	0.53	0.53	0.48	0.6	0.6	0.55
1mpy	17	0.53	0.53	0.48	0.53	0.53	0.48
2ozk	17	0.5	0.64	0.52	0.5	0.64	0.52
1xtz	17	0.5	0.54	0.47	0.64	0.69	0.63
2fa3	17	0.47	0.54	0.44	0.47	0.54	0.44
1i1a	17	0.47	0.54	0.44	0.47	0.54	0.44
2vhf	17	0.43	0.46	0.38	0.5	0.54	0.47
1vld	17	0.4	0.55	0.41	0.47	0.64	0.5
2dq3	17	0.38	0.42	0.34	0.46	0.5	0.43
1t5e	17	0.33	0.36	0.27	0.33	0.36	0.27
2hh9	17	0.27	0.29	0.19	0.36	0.36	0.28
1ymf	17	0.27	0.31	0.21	0.27	0.31	0.21
2gcj	18	0.73	0.79	0.73	0.73	0.79	0.73
1s7j	18	0.62	0.62	0.58	0.62	0.62	0.58
2hez	18	0.56	0.64	0.56	0.56	0.64	0.56
3e1k	18	0.53	0.53	0.48	0.6	0.6	0.56
2hii	18	0.53	0.53	0.48	0.53	0.53	0.48
1plr	18	0.53	0.53	0.48	0.53	0.53	0.48
2nua	18	0.5	0.62	0.51	0.5	0.62	0.51
2abr	18	0.44	0.54	0.43	0.53	0.62	0.53
2g7k	18	0.4	0.46	0.37	0.4	0.46	0.37
2uwy	18	0.38	0.5	0.38	0.38	0.5	0.38
1ra0	18	0.38	0.43	0.34	0.38	0.43	0.34

1q6z	18	0.38	0.4	0.32	0.44	0.47	0.39
1n4w	18	0.38	0.43	0.34	0.38	0.43	0.34
1ga6	18	0.38	0.5	0.38	0.44	0.58	0.46
1t5r	19	0.56	0.6	0.54	0.6	0.6	0.56
2ep7	19	0.53	0.56	0.5	0.59	0.62	0.56
2p0a	19	0.5	0.57	0.49	0.56	0.64	0.56
1h79	19	0.47	0.54	0.46	0.56	0.69	0.59
1lqt	19	0.44	0.5	0.42	0.44	0.5	0.42
1wvf	19	0.41	0.47	0.38	0.44	0.47	0.4
1sez	19	0.38	0.43	0.34	0.38	0.43	0.34
2jbk	19	0.33	0.36	0.28	0.33	0.36	0.28
2nqm	19	0.31	0.36	0.27	0.31	0.36	0.27
1d5t	19	0.29	0.36	0.26	0.29	0.36	0.26
1uv4	20	0.76	0.87	0.8	0.82	0.93	0.87
1tl2	20	0.75	0.8	0.75	0.75	0.8	0.75
2hkl	20	0.47	0.62	0.5	0.5	0.62	0.52
1xov	20	0.41	0.5	0.41	0.41	0.5	0.41
2gso	20	0.25	0.29	0.2	0.25	0.29	0.2
2i5v	21	0.88	0.83	0.84	0.94	0.89	0.91
2hox	21	0.56	0.64	0.57	0.62	0.71	0.64
2d5w	21	0.56	0.67	0.58	0.56	0.67	0.58
1e9g	21	0.56	0.67	0.58	0.59	0.67	0.6
1jov	21	0.5	0.5	0.45	0.5	0.5	0.45
1yew	21	0.42	0.5	0.41	0.42	0.5	0.41
2iho	21	0.39	0.37	0.32	0.39	0.37	0.32
1wth	21	0.39	0.37	0.32	0.39	0.37	0.32

1lpb	21	0.39	0.39	0.33	0.5	0.5	0.45
1kjq	21	0.33	0.38	0.3	0.33	0.38	0.3
1j5q	22	0.26	0.31	0.23	0.26	0.31	0.23
2d5h	22	0.24	0.22	0.17	0.35	0.33	0.29
2idb	23	0.58	0.65	0.58	0.58	0.65	0.58
3dk9	23	0.5	0.59	0.51	0.5	0.59	0.51
3c9f	23	0.35	0.41	0.33	0.42	0.47	0.4
1ulj	23	0.35	0.37	0.31	0.4	0.42	0.36
2vrk	24	0.62	0.65	0.61	0.62	0.65	0.61
1hx0	24	0.58	0.65	0.59	0.63	0.71	0.64
3dcd	24	0.5	0.56	0.49	0.5	0.56	0.49
2d1s	24	0.45	0.53	0.45	0.45	0.53	0.45
1gle	24	0.43	0.53	0.44	0.43	0.53	0.44
2qjv	24	0.3	0.33	0.27	0.35	0.39	0.32
1tfm	25	0.62	0.76	0.67	0.62	0.76	0.67
1jlx	25	0.6	0.63	0.59	0.6	0.63	0.59
2ex3	25	0.41	0.45	0.39	0.41	0.45	0.39
1ipk	25	0.3	0.35	0.27	0.36	0.4	0.34
1z50	26	0.77	0.85	0.8	0.77	0.85	0.8
1hyo	26	0.41	0.47	0.4	0.41	0.47	0.4
2e4t	26	0.35	0.36	0.31	0.45	0.45	0.42
1xhb	26	0.3	0.39	0.3	0.3	0.39	0.3
2qns	27	0.82	0.9	0.85	0.82	0.9	0.85
2qe8	27	0.76	0.84	0.79	0.76	0.84	0.79
2hj0	27	0.55	0.57	0.53	0.55	0.57	0.53
1gk9	27	0.55	0.6	0.54	0.55	0.6	0.54

2pxz	27	0.42	0.48	0.41	0.43	0.48	0.42
3eg9	27	0.33	0.38	0.31	0.33	0.38	0.31
2z2p	28	0.83	0.9	0.86	0.83	0.9	0.86
1yfq	28	0.78	0.86	0.81	0.78	0.86	0.81
1c9i	28	0.7	0.76	0.71	0.74	0.81	0.76
2jf3	28	0.29	0.2	0.2	0.29	0.2	0.2
2ce9	30	0.75	0.78	0.75	0.75	0.78	0.75
2bhu	30	0.43	0.52	0.44	0.44	0.52	0.45
2j3l	31	0.46	0.54	0.47	0.5	0.58	0.51
1nex	32	0.73	0.79	0.75	0.77	0.83	0.79
1hmw	33	0.54	0.58	0.53	0.54	0.58	0.53
1w2z	33	0.5	0.58	0.51	0.5	0.58	0.51
1qks	35	0.82	0.88	0.85	0.82	0.88	0.85
2vpm	35	0.5	0.64	0.54	0.5	0.64	0.54
2exj	42	0.51	0.55	0.51	0.51	0.55	0.51
