# Supplementary material for: β-sheet Topology Prediction with High Precision and Recall for β and Mixed α/β Proteins
Source: PLoS One. 2012 Mar 9;7(3):e32461. doi: 10.1371/journal.pone.0032461 (PMC3302896; doi:10.1371/journal.pone.0032461)
Supplement: Text S1 — Analysis of number of possible -sheets, elimination of circular paths and constraint statistics. (PDF) [file pone.0032461.s007.pdf]

1 Methods

1.1 Computational Complexity of β-sheet topologies

The model presented below only deals with flat β-sheets, and avoids the prediction of β-barrels. A flat β-sheet can be described as one a circular path cannot be drawn from any β-strand back to itself by tracing the path of the hydrogen bonding network. In order to make sure that the same β-sheet topology is not counted twice by rotating our view of the sheet, the following pair of restrictions are introduced:

1. The position of the first strand in the sequence, starting from the N terminus is within the first $[(n+1)/2]$, where n is the total number of strands. This would ensure that the rotation of the image would not result in the counting of a new β-sheet topology.

2. The orientation of the first strand is taken to be fixed to, without loss of generality, "up". This way, the rotation of the protein on the other axis does not result in the duplication of count of the β-sheet.

Using the aforementioned rules, for any β-sheet of n strands, there are $n!$ factorial ways of arranging them. By including the 2^n ways of arranging the β-sheets, and including the elimination of the axes of symmetry, we would have

$$1/4 X n! X 2^n = n! X 2^{n-2}$$

possibilities for an n-stranded β-sheet. Table S1 thus provides the number of possible arrangements of β-strands in proteins with number of strands between 2 and 11.

1.2 Elimination of circular paths

Since the objective is to maximize the contact potential between strands, most solutions would be cyclic in nature. Since the fraction of proteins which form β barrels is much smaller than proteins which don't, we choose to eliminate the possibility of all barrel-like

structures. On the other hand, if barrel structures were permitted, it was observed that barrel-like results were obtained for 96.4% of proteins. It is not possible to eliminate all circular solutions in the form of constraints. This is because we look to eliminate cyclic, as well as sub-cyclic solutions. For a protein with 20 strands, the number of possibilities exceed 10^5. The addition of such a large number of constraints would be extremely detrimental to the speed and performance of the algorithm. Hence, each solution obtained is checked for circular nature. If it is so, we do not increase the solution counter, and instead directly add an integer cut to eliminate this solution. The algorithm used for this purpose is described below.

We first acknowledge the fact that the arrangement of β-strands, obtained as a solution to the optimization model previously presented, is an undirected graph. This means that if one represents the nodes of the graph as the strands, and the edge of the graph as the presence of a contact, then the contact is commutative in nature. A depth-first search (DFS) algorithm can create a tree structure to the solution, starting from the root (say strand 1). We define a node edge structure for each strand with the following parameters: boolean $Visited$ to represent if we have traversed through this node in the graph, integer $parent$ determining the predecessor of the current node, an array of contacts $Contacts$ representing the set of contacts for the strand, along with the boolean array $ContactVisited$, representing if we reached the contact through the current strand, and finally the type of edge between the current strand and the one we move onto from here (*tree* or *back*). The description below explains these types of edges.

A DFS iteration can be represented by the following steps:

1. If this is the first step, take strand 1 as current strand. Otherwise select the current active strand

2. If one or more strands of $ContactVisited(i)$ are 0, let strand j be the first of such strands.

- If $Visited(j) = 1$, mark edge as *back* and return true for cyclic graph.
- Otherwise set $parent(j) = i$, $ContactVisited(i, j) = true$, $ContactVisited(j, i) = true$. Set current strand as j.

3. Otherwise set strand k as current strand, where $parent(i) = k$.
4. If current strand is strand 1, and it is not the first iteration, terminate and return false for subcycle.

If there are multiple sheets in the final prediction, we then repeat the above algorithm with a strand from the second sheet as the starting strand.

2 Analysis of Constraint Statistics

This section presents statistical evidence to support the incorporation of a number of constraints in the β-sheet topology prediction model. Each constraint is described, followed by the instances of success and failure of the constraint when applied to the PDBSelect25 data set.

A strand residue can have a maximum of two contacts. However, this does not mean that the strand itself can only have two contacts. It is possible for a long strand to pair up with more than one strand on one side. Hence, the maximum number of contacts a strand can make is taken as 3. In the entire set of proteins that were tested, only four proteins had one strand with four contacts. At the same time, it is required that each strand have atleast one contact. These constraints can be represented as:

$$\sum_{j \neq i} y(i,j) \leq 2 \quad \forall\, i, Strand(i) \neq Strand(j) \tag{1}$$

$$\sum_{sj \neq si} w_{AP}(si,sj) + \sum_{sj \neq si} w_P(si,sj) \leq 3 \quad \forall\, si \tag{2}$$

$$\sum_{sj \neq si} w_{AP}(si,sj) + \sum_{sj \neq si} w_P(si,sj) \geq 1 \quad \forall\, si \tag{3}$$

PDBSelect25 Statistics: No amino acid was seen to have more than two contacts for any protein. A minimum of one contact is true for all strands in PDBSelect25. Only four proteins were seen to have one strand each with four contacts. No protein was seen to have any strand with more than four contacts.

For a non barrel structure, the total number of contacts would not exceed $N_{str} - 1$, where N_{str} is the total number of strands in the protein

$$\sum_{si} \sum_{sj} w_{AP}(si,sj) + \sum_{si} \sum_{sj} w_P(si,sj) \leq N_{str} - 1 \tag{4}$$

PDBSelect25 Statistics: This constraint is very general, and is satisfied for all proteins which do not have any strand with four contacts. Further, the upper bound is reached only

for proteins with one β-sheet.

Since hydrogen bonding and hydrophobic collapse are believed to be the driving force for the coming together of β-strands to form sheets, the strands aim to minimize exposed area. Moreover, since β sheets typically form the core of the protein, the possibility of unsatisfied side chains forming hydrogen bonds with the solvent reduces. This exposed area comes about when two unequal strands form a contact, or when a contact is off-centre. In order to ensure that strands with similar lengths bind, and that the hydrogen bonding requirements of the strand are satisfied, we enforce that the total residues contacting a given strand should lie between $len_{si} - 2$ and $2len_{si} + 3$, where len_{si} is the length of the strand si. For this, we introduce parameters $NcontactAP(si, sj)$ and $NcontactP(si, sj)$, which can be defined as:

$$NcontactAP(si, sj) = \sum_{i \in si} \sum_{j \in sj} ResidueContactAP(i, j) \quad \forall sj > si \tag{5}$$

$$NcontactP(si, sj) = \sum_{i \in si} \sum_{j \in sj} ResidueContactP(i, j) \quad \forall sj > si \tag{6}$$

The constraint can hence be written as:

$$\sum_{sj \neq si} w_{AP}(si, sj) * NcontactAP(si, sj) + \sum_{sj \neq si} w_P(si, sj) * NcontactP(si, sj) \geq len_{si} - 2 \tag{7}$$

$$\sum_{sj \neq si} w_{AP}(si, sj) * NcontactAP(si, sj) + \sum_{sj \neq si} w_P(si, sj) * NcontactP(si, sj) \leq 2 * len_{si} + 3 \tag{8}$$

PDBSelect25 Statistics: For all β-strands in the PDBSelect25 data set (including all β and mixed α/β proteins), we find a success rate of 99.71% for the lower bounding expression shown above. Even among the remaining 0.29% of β-strands, 0.22% fall within an error of one amino acid of the allowed lower bound. For the upper bound, we find a success rate of 99.92% among all β-strands in the PDBSelect25 data set. By reducing the upper bound to $2len_{si} + 1$, the success rate goes down to 99.85%, and can be used as a means to tighten the upper bounding constraint.

Similar constraints can be written involving parallel contacts. Further, it was observed that for strands making three antiparallel contacts, at least one contact was made with its neighbors, or one of the edge strands. A number of strands forming 3 contacts made their third contact with a very small strand, which was typically either its own neighbor (by merely proving to be a small extended region following a β-turn) or at either end of the protein sequence, thus resulting in a much smaller impact on entropy loss. This constraint can be written as

$$\begin{aligned}\sum_{sj \neq si} w_{AP}(si, sj) &\leq w_{AP}(1, si) + w_{AP}(si-1, si) \\ &+ w_{AP}(si, si+1) + w_{AP}(si, N) + 2 \end{aligned} \tag{9}$$

PDBSelect25 Statistics: In the PDBSelect25 data set, 396 β-strands were seen to have three contacts. Among these, 374 (94.44%) β-strands were seen to satisfy the constraint mentioned above.

Past and recent work in literature have aimed to predict the total number of hydrogen bonds in a protein, given the number of amino acids of the protein. Using a small set of β proteins to derive a linear expression for the total number of hydrogen bonds, N_H, a linear relationship is given as:

$$N_H = 0.714 * N - 6.8 \tag{10}$$

where N is the number of amino acids of the protein. More recently, a much larger data set of proteins was used to derive a modified linear expression of the form:

$$N_H = 0.678 * N - 3.35 \tag{11}$$

Both of these equations predict the total number of hydrogen bonds in a globular protein. For the β-sheet prediction algorithm presented in this article, primary interest lies among the backbone hydrogen bonds formed between amino acids in the β-strands of the protein.

In a work studying hydrogen bonding patterns in globular proteins, the absolute number of hydrogen bonds (N_{HB}) formed in a protein could be expressed mathematically as:

$$N_{HB} = 1.49 f_\alpha * N + 0.65 f_\beta * N + 0.5 * (1 - f_\alpha - f_\beta) * N \tag{12}$$

where f_α and f_β are the fraction of α-helical and β-strand residues in the protein, respectively. From the expression, we can see that the three terms on the right hand side represent the expected contributions of the helical, extended and coil regions, respectively, to the total number of hydrogen bonds in the protein. Using this expression, restrictions are introduced on the total number of hydrogen bonds (or "contacts") between amino acids in β-strands, by allowing a 15% error range around the predicted value. Mathematically, this can be written as:

$$N_{HB,min} \leq \sum_i \sum_j y(i,j) \leq N_{HB,max} \; \forall i \in si; j \in sj, sj > si \tag{13}$$

PDBSelect25 Statistics: The success rate for the lower and upper bounds in the constraint shown above are 93.21% and 95.14%, respectively. The main aim of this set of constraints is to reduce erroneous over-prediction of contacts, thus reducing the number of false positives predicted.

One of the arrangements of β-strands conspicuous by its absence is commonly referred to as the "pretzel". For any quartet of β-strands (si, sj, sk, sl) which lie in the same β-sheet, such that $si < sj < sk < sl$, this constraint prevents the possibility of arrangements which result in the four strands lining up as (sk, si, sl, sj) or (sj, sl, si, sk). Mathematically, this restriction can be written as:

$$w_{AP}(si, sk) \leq 2 - w_{AP}(si, sl) + w_{AP}(sj, sl) \tag{14}$$

PDBSelect25 Statistics: The success rate for this constraint is observed to be 99.21% among all proteins in the PDBSelect25 data set.

A similar equation can be written for parallel contact between strands, and for combinations of the two kinds of contacts. Another class of contacts which have been observed to be constantly avoided is known as the layer crossover. According to this rule, for two pairs of sequentially consecutive β-strands starting at strand positions si and sj, the strand combination $(si+1, sj+1, si, sj)$ is avoided. This condition is an indirect consequence of the loop crossing conditions presented by Richardson and Gelfand and co-workers. This can be mathematically presented as:

$$w_{AP}(si+1, sj+1) \leq 2 - w_{AP}(si, sj+1) + w_{AP}(si, sj) \quad (15)$$

PDBSelect25 Statistics: The success rate for this constraint is observed to be 97.14% among all proteins in the PDBSelect25 data set.

As before, a circular nature for the identification of strands is used, wherein the first strand is assumed to be "following" the last strand of the primary sequence. Recent work has shown specific patterns that have emerged out of the analysis of β-sandwich proteins. As the name suggests, these proteins are characterized by a pair of β-sheets packed against each other like a sandwich. The first observation was the absence of parallel contacts between strands. Further, it was observed that for any non-local strand pairing (si, sj) in one sheet, a counter-balancing non-local contact between $si+1$ and $sj+1$ is observed in the opposite sheet, thus forming an "interlock". These constraints cannot be directly applied to our model, since the aim is to able to develop a prediction algorithm for any kind of β or mixed α/β protein. Hence, we generalize this condition to include any quartet of strands (si, sj, sk, sl) such that $si < sk < sj < sl$ and claim that an interlock is formed between strand pairs (si, sj) and (sk, sl), given by the following constraint:

$$\sum_{sk}\sum_{sl} w_{AP}(sk, sl) \geq \sum_{si}\sum_{sl} w_A P(si, sj) \quad (16)$$

PDBSelect25 Statistics: The success rate for this constraint is observed to be 91.81% among all proteins in the PDBSelect25 data set. The highest number of instances when the

constraint is not satisfied involves β-strands which are contacting three other β-strands of the protein.

3 β-sandwich proteins

A number of rules pertaining to β-strand arrangements in β-sandwich proteins have been presented previously in literature (see main text). Here, we carried out a detailed study of the proposed constraints for β-sandwich proteins. In all the β and mixed α/β proteins considered, it was seen that the β-sandwich proteins, as defined by SCOP, consisted of proteins which had between six and twenty strands. For proteins with a large number of strands, two β-sheets represent the formation of the β-sandwich, even though a large number of additional β-sheets may be present in the protein. The folding rules proposed for the formation of β-sandwich structures were tested on the pair of β-sheets that formed the sandwich. While carrying out this analysis, all β-strands which lie outside the β-sandwich were ignored.

The first rule for β-sandwich proteins describes the arrangement of structures known as a "strandon". As described in the original article, a strandon is defined as "a set of sequentially consecutive strands that are connected by hydrogen bonds in a β-sheet." Based on a description of the strandon structure, we carried out an analysis of the presence of strandon structures in β-sandwich segments of proteins in the PDBSelect25 data set. It was seen that the arrangement of strandons described in the main article (Chiang *et al.*, 2007) is satisfied for 94.1% of all sandwich structures in the PDBSelect25 data set.

The second rule for β-sandwich proteins describes the arrangement of β-strands within a strandon. The authors propose that within a strandon, the strand with the highest number (the numbering for strands starts from the N-terminus) is located at the end of the strandon. Further, for any consecutive strandons on the same β-sheet, or consecutive in sequence, strand numbers increase in opposite directions. For the PDBSelect25 data set, 79.4% of sandwich structures satisfy these conditions.
